# Quantum processor-inspired machine learning in the biomedical sciences

**DOI:** 10.1016/j.patter.2021.100246

**Published:** 2021-04-28

**Authors:** Richard Y. Li, Sharvari Gujja, Sweta R. Bajaj, Omar E. Gamel, Nicholas Cilfone, Jeffrey R. Gulcher, Daniel A. Lidar, Thomas W. Chittenden

**Affiliations:** 1 Department of Chemistry, University of Southern California, 920 Bloom Walk, Los Angeles, CA 90089, USA; 2 Computational Biology and Bioinformatics Program, Department of Biological Sciences, University of Southern California, Los Angeles, CA, USA; 3 Center for Quantum Information Science & Technology, University of Southern California, Los Angeles, Boston, CA, USA; 4 Computational Statistics and Bioinformatics Group, Genuity AI Research Institute, Genuity Science, 90 Canal Street, Suite 120, Boston, MA 02114, USA; 5 Complex Biological Systems Alliance, Medford, MA, USA; 6 Cancer Genetics Group, Genuity Science, Boston, MA, USA; 7 Department of Electrical Engineering, University of Southern California, Los Angeles, CA, USA; 8 Department of Physics and Astronomy, University of Southern California, Los Angeles, CA, USA; 9 Division of Genetics and Genomics, Boston Children's Hospital, Harvard Medical School, Boston, MA, USA

**Keywords:** machine learning, cancer genomics, The Cancer Genome Atlas

## Abstract

Recent advances in high-throughput genomic technologies coupled with exponential increases in computer processing and memory have allowed us to interrogate the complex molecular underpinnings of human disease from a genome-wide perspective. While the deluge of genomic information is expected to increase, a bottleneck in conventional high-performance computing is rapidly approaching. Inspired by recent advances in physical quantum processors, we evaluated several unconventional machine-learning (ML) strategies on actual human tumor data, namely “Ising-type” methods, whose objective function is formulated identical to simulated annealing and quantum annealing. We show the efficacy of multiple Ising-type ML algorithms for classification of multi-omics human cancer data from The Cancer Genome Atlas, comparing these classifiers to a variety of standard ML methods. Our results indicate that Ising-type ML offers superior classification performance with smaller training datasets, thus providing compelling empirical evidence for the potential future application of unconventional computing approaches in the biomedical sciences.

## Highlights

• Ising-type algorithms perform better with small datasets
• There is less overfitting with Ising-type algorithms
• Ising-type algorithms are amenable for drug discovery
• Ising-type algorithms are useful for assessment of drug efficacy

## The bigger picture

Advances in sequencing technology, leading to an ever-increasing volume and variety of data, present an opportunity to probe the molecular underpinnings of disease. Inspired in part by recent developments in physical quantum processors, we evaluated several Ising-type algorithms, which are relatively unused in the biomedical sciences, on actual human tumor data from The Cancer Genome Atlas. Our results show performance competitive with conventional machine-learning algorithms in classifying human cancer types and associated molecular subtypes when training with all available data; but perhaps more strikingly, the Ising-type algorithms demonstrate superior performance with smaller training datasets. This gain in performance suggests a tantalizing application for rare diseases or other clinical applications where the number of training samples may be quite small. In addition, the features extracted from the Ising-type algorithms have biological relevance.

## Teaser

Inspired by quantum process, Ising-type algorithms demonstrate better classification performance than standard machine learning approaches when training with small amounts of data. This suggests a potential application for rare diseases or other clinical data where the amount of training data may be limited.

## Introduction

With the rapid expansion of high-throughput genomic technologies there exists a multitude of “omics” data, which allows researchers to now investigate the causal molecular drivers of complex human disease with a systems biology approach. Over the past 2 decades, numerous studies have shown the utility of statistical machine-learning (ML) strategies to classify human malignancies, hypothesize unknown clinical subtypes, and make prognostic predictions based on omics datasets.^1^^,^^2^ Moreover, integrated “multi-omics” approaches have proved effective in deriving meaningful biological insights into the etiological and prognostic complexity of human cancers.^3^, ^4^, ^5^, ^6^ While these studies highlight the potential of omics-based analytics to drive innovative new therapies based on unique molecular signatures, several well-documented issues, including correlation bias, feature dependency, and multicollinearity, still hamper statistical optimization for the analysis and robust classification of high-dimensional complex biological datasets.^7^

To address some of these statistical computing limitations, we present a class of unconventional “Ising-type” ML algorithms, inspired by quantum computing. As a rapidly emerging technology, quantum computing promises to enhance the performance of certain classes of statistical computing and ML tasks, such as classification, regression, generation, and resampling. In this nascent discipline, proposals for several quantum ML algorithms have been developed, including quantum principal-component analysis (PCA)^8^ and quantum support vector machines^9^ and Boltzmann machines.^10^ These proposals have generated interest in the scientific community and in the general public for their potential to address computationally intractable tasks and to model more complicated data distributions. One of the unconventional ML approaches used in this study, quantum annealing with processors made by D-Wave Systems,^11^, ^12^, ^13^ features more than 2,000 qubits, becoming large enough to solve real-world problems,^14^ perform quantum simulation,^15^ and compete with classical optimization algorithms.^16^ While the computational role of quantum effects in these processors remains controversial and the subject of intensive study, quantum annealing is currently one of the few paradigms of quantum computing that are approaching a scale useful for practical applications.

Using high-dimensional multi-omics human cancer data from The Cancer Genome Atlas (TCGA), we framed a classification problem in such a way that it was amenable to solving with Ising-type approaches. The Ising-type methods must be formulated as a quadratic unconstrained binary optimization (QUBO) or, equivalently, an Ising Hamiltonian $H\left(w\right)=w^{⊺}h+w^{⊺}Jw$, where w is a vector of weights, and h and J represent a vector and a matrix, respectively. We compared Ising-type approaches to standard ML approaches for both binomial and multiclass experimental designs. Although previous studies have applied quantum annealing and other Ising models to model protein folding,^17^ transcription factor DNA binding,^18^ and classification of lung cancer data with microarray data,^19^ our analysis is the first of integrated, genome-wide multi-omics human cancer data. In the course of our study, we found that the Ising models all perform similarly to each other. Our results further show that, in most cases, when using relatively large amounts of high-dimensional multi-omics training data, the Ising-type methods are comparable to standard supervised ML approaches. However, for smaller training datasets of equivalent dimensionality, Ising models statistically outperform established classification strategies. We also assessed the weights returned by the Ising models and found reasonable interpretability and generalizability of biological information. Overall, our results demonstrate the current utility and limitations of Ising models applied to the analysis of high-dimensional omics data and point to a general class of algorithms that may be useful when training data are limited.

## Results

**Figure 1:**
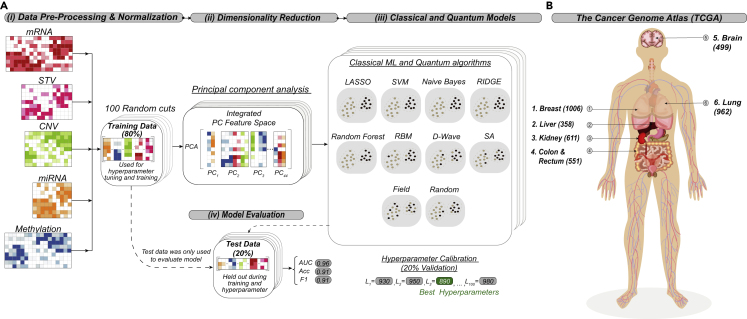
Overview of strategy and cancer types used in this study (A) Overview of classification strategy. (i) Whole-exome sequencing, RNA-seq, miRNA-seq, DNA methylation array, and genotyping array (for CNVs) data were retrieved from The Cancer Genome Atlas for human cancer type and molecular subtype classification. Data were concatenated and transformed into a single scaled omics data matrix. The matrix was then repeatedly split into 100 unique training and independent test sets representing 80% and 20% of the total data, respectively. After the data were split, each training split was scaled to have zero mean and unit standard deviation. The same scaling was then applied to the corresponding test split. (ii) Principal-component analysis (PCA) was performed separately on each individual training set, and a subsequent matched test set was projected using training-set-specific PCA loadings. (iii) Several standard classical machine-learning (ML) algorithms were compared with quantum annealing and several classical algorithms that have the same objective function as quantum annealing. The standard classical ML methods assessed included least absolute shrinkage and selection operator (LASSO), ridge regression (RIDGE), random forest, naive Bayes, and support vector machine (SVM). Quantum annealing (D-Wave) was performed on D-Wave hardware by formulating the classification problem as an Ising problem (see experimental procedures). These classical Ising-type approaches include simulated annealing (SA), candidate solutions randomly generated and sorted according to the Ising energy (Random), and an approach that considers only local fields of the Ising problem (Field). Hyperparameters were tuned on the train data using a 10-fold cross-validation (see supplemental experimental procedures for a description of the ranges of hyperparameters used). (iv) After training, classification performance was validated with each corresponding test set (unseen during the tuning of hyperparameters and the training) for a variety of statistical metrics, including balanced accuracy, area under the ROC curve (AUC), and F1 score. Classification performance metrics were averaged for the 100 test sets for each model to provide statistics on the mean performance. (B) The six human cancer types used for the multiclass classification models. Patient sample sizes are indicated in parentheses.

We assessed the performance of annealing-based Ising ML algorithms on several TCGA datasets to identify comparative advantages for the Ising approaches. In this ML survey, we compared the performance of Ising models to that of the following commonly used ML algorithms: least absolute shrinkage and selection operator (LASSO),^20^ ridge regression (Ridge),^21^^,^^22^ random forest (RF),^23^^,^^24^ naive Bayes (NB),^25^^,^^26^ and a support vector machine (SVM).^27^^,^^28^ TCGA data, including exome DNA variation, RNA sequencing (RNA-seq), DNA methylation, microRNA (miRNA), and copy number variations (CNVs), were retrieved, preprocessed, and normalized, resulting in an average of 70,504 gene features for five binomial and one multiclass six-cancer TCGA dataset comparison. We performed dimensionality reduction with PCA (see supplemental experimental procedures), retaining the top 44 principal components (PCs) for the binomial datasets and 13 PCs for the six-cancer dataset. The number of PCs was chosen based on the largest number of features that could be accommodated on existing quantum annealing hardware. An overview of our data analysis strategy is presented in Figure 1.

Quantum annealing was implemented on D-Wave physical quantum processors (see supplemental experimental procedures). As mentioned, D-Wave admits problems only when formulated as a QUBO problem or, equivalently, an Ising Hamiltonian, generically written as $H\left(w\right)=w^{⊺}h+w^{⊺}Jw$, where w is a vector of weights, J is the matrix of interactions, and h is the local fields. The goal of the learning procedure is to find an optimal set of weights that minimizes the energy of the Ising Hamiltonian, i.e., find $w^{*}=argmin_{w}H\left(w\right)$. The global optimum of the Ising problem is in general difficult to determine.^29^ Classification by using quantum annealing to solve Ising problems has been formulated before.^30^ In the present work, we developed a novel approach that can be used to solve classification problems directly. Our strategy stems from multinomial regression, which reduces to logistic regression when there are two classes (see supplemental experimental procedures for the mapping to an Ising problem). We compared the performance with several other Ising models that use the same objective function as D-Wave, i.e., problems formulated as an Ising Hamiltonian: simulated annealing (SA), Random, and Field. SA^31^ is a well-known heuristic optimization algorithm that uses Metropolis updates and a (fictitious) temperature schedule to optimize a target objective function. For Random, we randomly generated candidate weights, sorted them by their Ising energy, and selected the best performing weights. For Field, we disregarded J, the coupling terms, and performed an optimization only over h, the local fields (see supplemental experimental procedures for more details of all classical, quantum, and quantum-inspired algorithms). Both Random and Field were introduced and used as simple benchmarks against which we tested the SA and quantum annealing approaches.

Last, we compared the performance of the annealing-based models to a restricted Boltzmann machine (RBM), which is also based on an Ising model. Note, however, that the annealing-based Ising models described above are purely supervised learning approaches, i.e., formulated explicitly with a response variable to be predicted (in this case, the class of cancer). Boltzmann machines generally seek to explicitly model a data distribution over the inputs as a Boltzmann distribution by incrementally adjusting the h’s and J’s, whereas in our formulation the h’s and J’s are fixed given the input training data, and the mechanism of learning is to obtain solutions that minimize the Ising Hamiltonian, rather than to accurately reproduce a distribution. Accordingly, we will use “all Ising models” to refer to both the annealing-based Ising approaches and the RBMs, and “annealing-based” Ising approaches to refer exclusively to the annealing-based approaches.

### Binomial and multinomial classification

In this section, we present classification results for five binomial TCGA cancer dataset comparisons: kidney renal clear cell carcinoma (KIRC) versus kidney renal papillary cell carcinoma (KIRP), lung adenocarcinoma (LUAD) versus lung squamous cell carcinoma (LUSC), breast invasive carcinoma (BRCA) versus matched normal breast tissue (normal), estrogen receptor positive (ERpos) versus estrogen receptor negative (ERneg) breast cancers, and luminal A (LumA) versus luminal B (LumB) breast cancers. We also present findings relative to a six-cancer multiclass classification strategy for human brain, breast, kidney, lung, liver, and colorectal cancer types (see Table S1 for the sample sizes of each dataset). We assessed the relative classification performance of the five standard ML models (LASSO, Ridge, RF, NB, and SVM), one quantum algorithm (D-Wave), three annealing-based Ising algorithms (SA, Random, and Field), and one Ising model (RBMs) for all binomial and multiclass TCGA comparisons.

**Figure 2:**
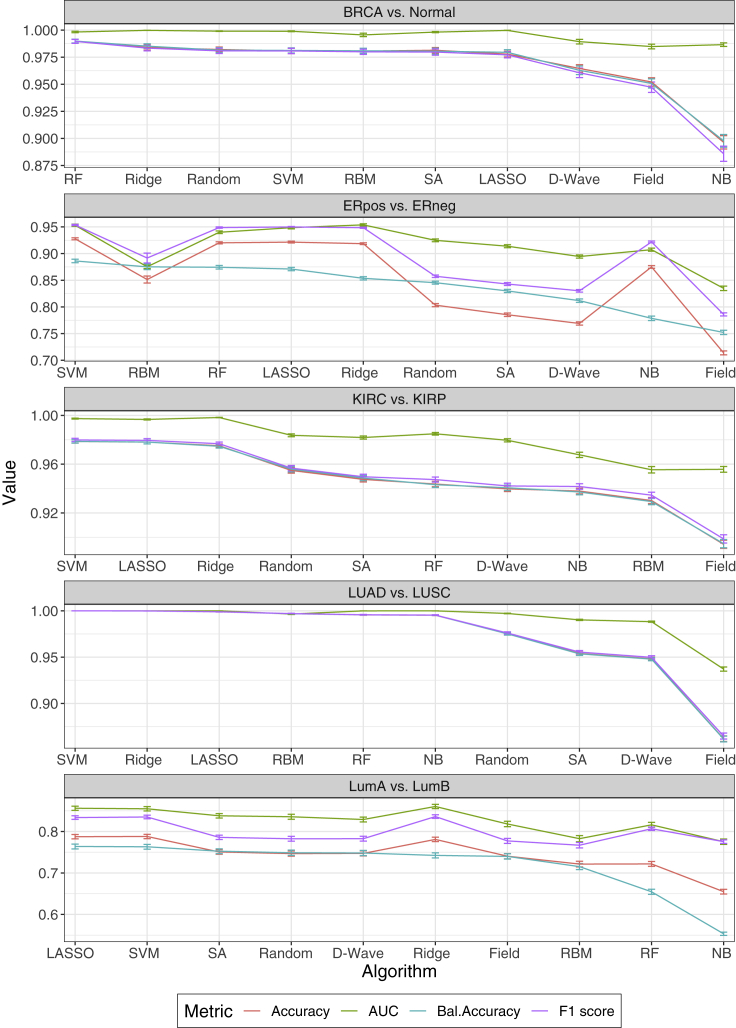
Comparison of classification algorithms for five TCGA cancer datasets Human cancer datasets assessed: breast invasive carcinoma (BRCA) versus matched normal tissue (normal), estrogen receptor positive (ERpos) versus estrogen receptor negative (ERneg) breast cancers, kidney renal clear cell carcinoma (KIRC) versus kidney renal papillary cell carcinoma (KIRP), lung adenocarcinoma (LUAD) versus lung squamous cell carcinoma (LUSC), and luminal A (LumA) versus luminal B (LumB) breast cancers. To address class imbalance for each comparison, algorithm performance is ranked by mean balanced accuracy on the x axis. By and large, the other metrics indicate the same performance ranking. Classification performance metrics were averaged for the 100 unique training and test sets for each model (see experimental procedures). Performance metrics: accuracy (red), AUC (green), balanced accuracy (blue), and F1 score (purple). Data are presented as the mean ± SEM.

Figure 2 presents comparisons of all 10 classifiers for the five binomial datasets. We used four statistical metrics to assess classification performance: accuracy, balanced accuracy, receiver operating characteristic (ROC) area under the curve (AUC), and F1 score. The four metrics were independently averaged over 100 unique training and test sets for each classifier (see supplemental experimental procedures). The mean ± SEM for each metric are presented on the y axis of each figure inset. See Table 1 for the values of the balanced accuracy and Tables S2–S4 for accuracy, AUC, and F1 score. Relative classification performance was determined by mean balanced accuracy and presented in ranked order on the x axis of each figure inset. Nonparametric Wilcoxon signed-rank tests were used to assess statistical significance among the 1010 classifiers relative to the four performance metrics. Bonferroni correction was used to adjust for multiple testing error. For each comparison, we found that a standard ML approach outperformed both quantum and classical annealing across all four metrics of performance. However, for several comparisons, at least one of the Ising-type algorithms performed nearly as well as the best classical method. For example, while RF statistically outperformed (0.99 ± 0.002) all other methods for the BRCA versus normal comparison, Random, SVM, RBM, SA, and LASSO showed no statistical differences in performance (0.98 ± 0.002; 0.98 ± 0.002; 0.98 ± 0.002; 0.98 ± 0.003; 0.98 ± 0.002). Similarly, for the LumA versus LumB comparison, we found that LASSO performed best (0.76 ± 0.006); however, Random, D-Wave, SA, Ridge, and Field were nearly identical in terms of balanced accuracy (0.75 ± 0.006; 0.75 ± 0.006; 0.75 ± 0.006; 0.74 ± 0.006; 0.74 ± 0.006). For the three other comparisons (ERpos versus ERneg, KIRC versus KIRP, LUAD versus LUSC), the annealing-based Ising algorithms statistically underperformed versus the best standard ML algorithm in each comparison, although RBM performed well. While D-Wave performed similar to RF and NB in the KIRC versus KIRP comparison (0.94 ± 0.002 versus 0.94 ± 0.002; 0.94 ± 0.002; corrected p = 1), it was statistically inferior to SVM (0.94 ± 0.002 versus 0.98 ± 0.001; corrected p = 5.96 × 10^−24^). Overall, Field was one of the poorest performing methods relative to the four metrics assessed; however, it performed relatively well on the LumA versus LumB dataset (0.74 ± 0.006). The quantum and classical Ising-type classification results indicate the utility of framing an overall classification strategy as an Ising problem.

**Table 1:** Balanced accuracies for five binomial comparisons and the one six-class cancer dataset used in this study

Dataset	LASSO	Ridge	SVM	RF	NB	D-Wave	SA	Random	Field	RBM
BRCA versus normal	0.982 ± 0.002	0.984 ± 0.002	0.981 ± 0.002	0.990 ± 0.002	0.897 ± 0.006	0.974 ± 0.003	0.981 ± 0.003	0.982 ± 0.002	0.951 ± 0.004	0.981 ± 0.002
ERpos versus ERneg	0.871 ± 0.003	0.854 ± 0.003	0.886 ± 0.003	0.874 ± 0.003	0.779 ± 0.004	0.812 ± 0.003	0.830 ± 0.003	0.845 ± 0.003	0.752 ± 0.004	0.875 ± 0.005
KIRC versus KIRP	0.978 ± 0.002	0.975 ± 0.002	0.979 ± 0.001	0.944 ± 0.002	0.937 ± 0.002	0.947 ± 0.002	0.949 ± 0.002	0.956 ± 0.002	0.895 ± 0.003	0.929 ± 0.002
LUAD versus LUSC	0.9988 ± 0.0002	0.9999 ± 0.0001	1.0000 ± 0.0000	0.9957 ± 0.0004	0.9953 ± 0.0004	0.9478 ± 0.0017	0.9536 ± 0.0017	0.9751 ± 0.0013	0.8616 ± 0.0031	0.997 ± 0.0004
LumA versus LumB	0.764 ± 0.006	0.742 ± 0.006	0.763 ± 0.006	0.655 ± 0.006	0.553 ± 0.004	0.748 ± 0.006	0.752 ± 0.006	0.749 ± 0.006	0.740 ± 0.006	0.715 ± 0.007
Six cancer	0.9896 ± 0.0002	0.9845 ± 0.0003	0.9891 ± 0.0002	0.9863 ± 0.0003	0.9735 ± 0.0004	0.9122 ± 0.0012	0.9170 ± 0.0010	0.9083 ± 0.0015	0.8876 ± 0.0005	0.9534 ± 0.0013

Data are reported as the mean ± SEM.

**Table 2:** Balanced accuracies when incrementally decreasing the amount of training for the LumA versus LumB comparisons

Fraction	LASSO	Ridge	SVM	RF	NB	D-Wave	SA	Random	Field	RBM
0.25	0.633 ± 0.012	0.630 ± 0.013	0.696 ± 0.007	0.560 ± 0.008	0.508 ± 0.002	0.737 ± 0.007	0.739 ± 0.007	0.732 ± 0.007	0.720 ± 0.009	0.737 ± 0.005
0.55	0.682 ± 0.011	0.718 ± 0.005	0.728 ± 0.006	0.566 ± 0.008	0.509 ± 0.003	0.740 ± 0.006	0.749 ± 0.004	0.752 ± 0.005	0.740 ± 0.007	0.739 ± 0.005
0.95	0.752 ± 0.002	0.749 ± 0.003	0.756 ± 0.003	0.609 ± 0.005	0.572 ± 0.004	0.755 ± 0.004	0.761 ± 0.004	0.757 ± 0.004	0.739 ± 0.005	0.740 ± 0.004

The fraction represents the amount of training data used, and results are reported as the mean ± SEM.

Although the Ising-type algorithms generally underperformed versus the standard ML methods assessed for these comparisons, the Ising-type classifiers performed well on the LumA versus LumB comparison. Moreover, as with all the standard ML methods used in this work, the most informative feature for classification predicted by the annealing-based Ising models was the first PC, indicating that the Ising models also assigned the greatest weight to the features that account for the most variation within the data. This is consistent with previous results where D-Wave was able to extract a motif for protein-DNA binding that agreed with classical results.^18^

Finally, to determine the utility of Ising-type methods on a larger, multiclass classification experimental design, we evaluated classification performance of the standard and Ising-type ML algorithms on a six-cancer, multiclass TCGA dataset. The six TCGA cancer types included brain, breast, kidney, lung, liver, and colorectal cancers (see Table S1 for the sample size of this six-cancer dataset). With the exception of multiclass AUC (0.99 ± 0.0), performance metrics for standard ML approaches were superior to those of all Ising models for this larger, multiclass dataset (see also Figure S1). We, therefore, focused our efforts on further evaluating the efficacy of all the Ising models on the five binomial comparisons described above.

### Performance dependence on training set size

**Figure 3:**
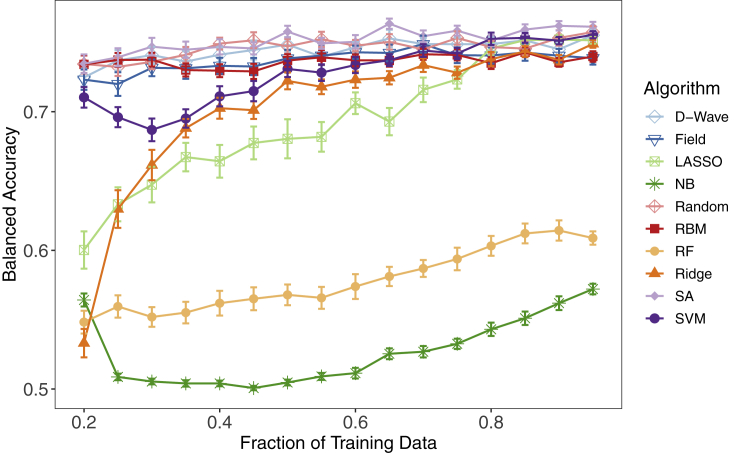
Test set balanced accuracy for LumA versus LumB binomial classification with incremental decreases from 95% to 20% of the original training set The algorithms evaluated are indicated in the legend. Averaged balanced accuracies were calculated for 50 independent training sets at each designated fraction of the original training data. Data are presented as the mean ± SEM.

Based on previous work indicating that quantum and classical Ising-type approaches are superior to standard ML classifiers on small training set sizes,^18^^,^^32^^,^^33^ we systematically reduced the training set data for the LumA versus LumB human breast cancer comparison into 16 separate partition sizes to evaluate classifier performance (see supplemental information). We first divided the entire LumA versus LumB breast cancer dataset (311 breast tumor samples) into a training set representing 80% of the initial dataset (250 breast tumor samples) and a testing set equal to 20% of the initial dataset (61 breast tumor samples). From this, we randomly selected incrementally smaller, class-balanced data partitions from 95% to 20% of the original training set data. Due to the complexity and computational expense of this experimental design, we trained each of the 10 classifiers described above over only 50 unique training sets randomly drawn from the 250 breast tumor samples of the initial training data, for each training set partition. We then validated the performance of each classifier on the original, held-out test set of 61 breast tumor samples. As above, nonparametric Wilcoxon signed-rank tests were used to assess statistical significance among the 10 classifiers relative to the four performance metrics, and Bonferroni correction was used to adjust for multiple testing error. The results in Figure 3 are presented as the mean ± SEM for averaged balanced accuracies across the entire training set size spectrum; see Table 2 for the balanced accuracies of all the algorithms at some representative training fractions.

At 25%–40% of the original training data (63 to 100 breast tumor samples), the mean balanced accuracies of the five Ising models (D-Wave, SA, Random, Field, and RBM) were statistically superior to the mean balanced accuracies of the five standard ML algorithms (LASSO, NB, RF, Ridge, SVM). For example, at 25% of the initial training data D-Wave statistically outperformed SVM, the top standard ML method (0.74 ± 0.007 versus 0.70 ± 0.007; corrected p = 1.94 × 10^−3^), as did the other Ising-type methods. Classification performance for all standard ML methods (SVM, LASSO, NB, RF, Ridge) steadily decreased after a reduction to 50% of the original training data (125 breast tumor samples), whereas we found significantly less reduction in mean balanced accuracies for the five quantum and classical Ising models across the entire training set size spectrum. Furthermore, the Ising models showed a relatively minimal reduction in performance at 95% versus 20% of the original training data (0.76 ± 0.004 versus 0.73 ± 0.007; corrected p = 0.092 for SA's performance) compared with LASSO (0.75 ± 0.002 versus 0.60 ± 0.01; corrected p = 3.32 × 10^−11^). Moreover, all five standard ML methods were associated with a significantly higher degree of overfitting than the Ising model classification approaches, an issue that has also historically plagued the analysis of genomic data. Figure S2A indicates significantly less statistical shrinkage relative to test data for the Ising model algorithms across all fractions of training data for the LumA versus LumB comparison. As an example, with 20% of the training data, although RBM and SVM perform fairly similarly in terms of the balanced accuracy on the test set (0.73 ± 0.007 versus 0.71 ± 0.007, p = 0.016), the overfitting, as measured as the difference between the training and the test balanced accuracy, is significantly higher for SVM than for RBM (0.29 ± 0.007 versus 0.17 ± 0.007, p = 9.37 × 10^−14^).

To assess the generality of this finding that Ising-type methods may perform better than standard ML approaches with a small amount of training data, we performed the same analysis on the ERpos versus ERneg breast cancer and the six-cancer, multiclass datasets. As both datasets were significantly larger than the LumA versus LumB comparison, we reduced each to a much smaller percentage of the initial training set size. Figure S3A presents mean balanced accuracies from 95% to 10% of the original training data (730 to 77 breast tumor samples) for the ERpos versus ERneg comparison. We found the same result in classification performance for all 10 classifiers; namely, a decrease in performance for the small training data for the standard ML methods, but little or no change for the Ising models. Unlike the LumA versus LumB comparison, the Ising models showed no statistical loss in performance from 95% to 10% of the original training data (0.84 ± 0.004 versus 0.84 ± 0.002; corrected p = 1 for SA); whereas RF dropped from 0.86 ± 0.002 to 0.81 ± 0.008 (corrected p = 1.13 × 10^−5^). Similar to the LumA versus LumB comparison, Figure S2B indicates that the Ising models generally have less overfitting across many of the training fractions; SVM had a higher degree of overfitting compared with SA (0.14 ± 0.006 versus 0.02 ± 0.005, p = 8.48 × 10^−17^).

Analysis of the six-cancer, multiclass dataset further confirmed the ERpos versus ERneg findings. While Figure S3B shows that the standard ML methods significantly outperformed the Ising-type methods, here again we found no statistical reduction in D-Wave performance (0.92 ± 0.001 versus 0.91 ± 0.002; corrected p = 1) from 95% (3,035 tumor samples) to 5% (163 tumor samples) of the initial training set size, although RBMs did better than the annealing-based Ising approaches. Comparatively, we again found a significant reduction in classification performance for LASSO (0.992 ± 0.0001 versus 0.978 ± 0.001; corrected p = 9.89 × 10^−16^) on this multiclass cancer dataset. In addition, SA also exhibited a significant performance drop relative to D-Wave at the low end of the training data fraction, although this feature is temperature dependent: by modifying SA's final temperature it can be made to perform as well as D-Wave. This is concordant with previous binomial quantum ML studies.^30^^,^^31^

In summary, all methods (with the exception of NB) converged to roughly the same balanced accuracy at a high training data fraction, but at a low fraction all Ising models performed better on three distinct datasets. These findings go beyond previous work^30^^,^^31^ and further bolster the case for the utility of framing an overall classification strategy as an Ising problem. Moreover, robust classification of small, high-dimensional omics datasets with Ising models provides a potential new avenue to evaluate patient response in the early phase of clinical drug trials or in other genome-wide datasets with relatively small numbers of patients or animal models.

### Gene-level classification

**Figure 4:**
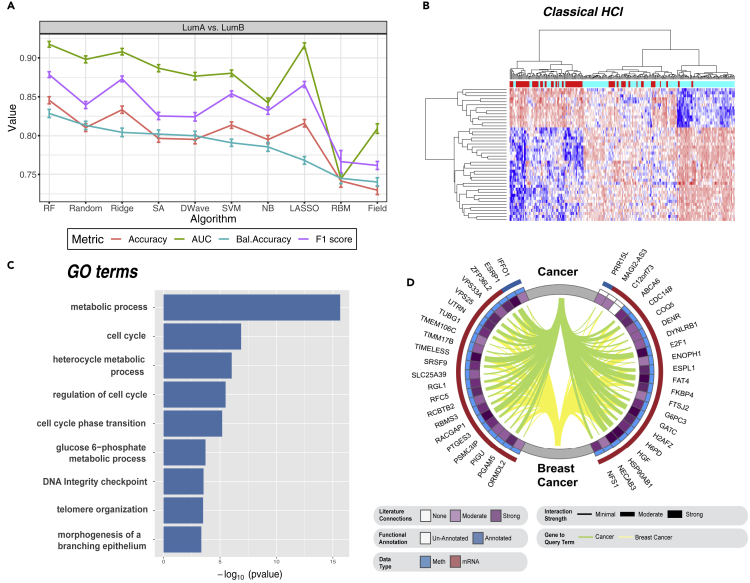
Classification, hierarchical clustering, functional enrichment, and natural language processing of the top 44 genes of PC1 for LumA versus LumB binomial comparison (A) Gene-level classification of LumA versus LumB human breast cancers based on the top 44 genes of PC1. Data are presented as the mean ± SEM. (B) Classical hierarchical clustering algorithm (see experimental procedures). Note: genes are presented in rows and samples in columns. (C) GOseq functional enrichment analysis of the top 44 genes for PC1 shows enriched GO terms ordered by p value. (D) Circos plot representing semantic search of full-text articles within the PubMed Central database identifying published associations of the top 44 genes for PC1 to the query terms *cancer* and *breast cancer*. The red and blue outer bands represent “mRNA” and “methylation” data types, respectively. The inner blue band represents genes with known functional annotation. The intensity of the inner purple ring indicates the total number of publications on cancer and breast cancer for the top 44 genes of PC1. This band has six colored bins, where white is the lowest and dark purple the highest number of publications at the time of analysis. The thickness and color of the Circos plot ribbons indicate the number of published gene-to-query term associations: green represents cancer and yellow designates breast cancer.

To assess the performance of the Ising-type methods at the gene level, we used the 44 most informative genes, by PCA loading of the first PC (PC1), from the original training set described in the previous sections for the LumA versus LumB breast cancer dataset. Results are presented in Figure 4A. The four metrics were independently averaged over 100 unique training and test sets for each of the 10 classifiers. Nonparametric Wilcoxon signed-rank tests were again used to assess statistical significance for the four metrics relative to the 10 classifiers. As above, Bonferroni correction was used to adjust for multiple testing error. Here we found a significant increase in mean balanced accuracies for all 10 classifiers at the gene level compared with PCA feature-based classification. For example, RF performed significantly better at the gene level compared with the PC level (0.83 ± 0.007 versus 0.65 ± 0.008; corrected p = 3.02 × 10^−31^). We also found that Random (0.81 ± 0.005), SA (0.80 ± 0.005), and D-Wave (0.80 ± 0.006) slightly outperformed three of the five standard ML approaches: SVM (0.79 ± 0.005), NB (0.79 ± 0.006), and LASSO (0.77 ± 0.005). To confirm the multi-omics PCA-derived gene-level classification findings, we performed a simple dual-dimensionality reduction and differential analysis approach on the LumA versus LumB comparison with edgeR.^34^ Briefly, edgeR fits a negative binomial distribution to assess whole-transcriptome gene expression. In this second analysis, the top 44 differentially expressed mRNAs were used for gene-level classification in the same manner as described above. Given that edgeR gene-level classification was comparable to PCA gene-level findings (Figure S4), we used the features from PC1 to take advantage of the enhanced molecular information content of our multi-omics approach.

Close inspection of the top 44 genes from PC1 used as molecular features for the LumA versus LumB comparison indicated that *RACGAP1* was the most informative feature, as averaged across 9 of the 10 classifiers (see Table S5; RBMs were not included because of the difficulty in assessing feature importance). This finding was further supported via an independent edgeR^34^ analysis, which showed that *RACGAP1* was the strongest differentially expressed gene (false discovery rate [FDR] = 2.57 × 10^−36^; logFC = −1.11) of the top 41 mRNA genes. Figure S5 presents a rank-ordered heatmap of the averaged state for each of the 44 genes (41 mRNA and 3 methylated genes) across the 100 unique training sets for the LumA versus LumB comparison. Conversely, *RACGAP1* was ranked only 22 of 44 by PC1 loading. These findings indicate the importance of combined dimensionality reduction/feature learning and classification of high-throughput biological data. From a biological perspective, *RACGAP1* is a putative oncogene, which promotes the growth of triple-negative/basal-like breast cancers. Experimental depletion of this gene inhibits cancer cell proliferation by the combined effects of cytokinesis failure, *CDKN1A/p21*-mediated *RB1* inhibition, and the onset of senescence.^35^ Given the significant increased expression of *RACGAP1* in LumB tumors, the more aggressive breast cancer subtype, our gene-level classification results also support our previous findings indicating that Ising-type models are capable of robustly assigning the greatest weight to the most biologically relevant information in a given model. Figure 4B shows hierarchical clustering of the 44 most informative genes for the LumA versus LumB breast cancer comparison and indicates significant discrimination between LumA and LumB based on these 44 genes.

Finally, we used GOseq analysis^36^ and a PubMed Central (PMC) comprehensive semantic search engine to determine the known biological relevance of the top 44 genes in the LumA versus LumB breast cancer comparison. Our GOseq analysis produced 244 functionally enriched gene ontology (GO) terms (see Table S6). Of these, Figure 4C presents nine statistically significant (Wallenius approximation; FDR ≤ 0.05) GO terms related to cancer: metabolic process, cell cycle, heterocycle metabolic process, regulation of the cell cycle, glucose 6-phosphate metabolic process, DNA integrity checkpoint, telomere organization, and morphogenesis of a branching epithelium. We then used a semantic search engine to query full-text records available in PMC database for published relationships between these 44 genes and the query terms *cancer* and *breast cancer* (see supplemental experimental procedures). Briefly, we used the entrez search function of the rentrez R package, which provides an NCBI EUtils application programming interface,^37^ to retrieve results for each of the 44 genes from the PMC database. Search terms were defined by combining each gene symbol with either *cancer* or *breast cancer* fields, along with all related MeSH terms using the Boolean operators AND/OR.

We found that all but *C12orf73* have been previously indicated in breast cancer (Figure 4D). Of the remaining 43 genes, *PRR15L* and *MAGI2-AS3* are the only genes with no current functional annotation; however, both *PRR15L* and *MAGI2-AS3* associate with a high averaged information ranking for the LumA versus LumB comparison (see Table S5). At the time of our semantic search of the PMC database, hepatocyte growth factor (*HGF*) and retinoblastoma-associated protein 1 (*E2F1*) were implicated in the greatest number of published breast cancer papers (6,356 and 5,925, respectively) among all of the 44 genes queried (see Table S7). *E2F1* yielded higher PC1 loading (4 versus 15) and averaged information (8.6 versus 33) rankings than *HGF*. *E2F1* is a well-studied transcription factor involved in cell proliferation, differentiation, and apoptosis. It is a member of the E2F protein family, which has been implicated in cell-cycle control and regulation of tumor suppressor proteins. Low *E2F1* gene expression is predictive of metastasis-free survival in breast cancer patients.^38^ As with our *RACGAP1* finding, we determined significantly higher differential mRNA expression of *EF2F1* in LumB versus LumA breast cancers via edgeR analysis (FDR = 2.59 × 10^−27^; logFC = −1.34). Taken together, our gene-level classification results support known breast cancer etiology.

## Discussion

We have presented the first successful demonstration of annealing-based Ising models applied to integrated genome-wide multi-omics human cancer data. We have shown that classification with Ising models is comparable to standard ML strategies on multiple partitions of data of multiple large human cancer datasets. However, it is important to note that the benefit of using quantum annealing cannot be attributed solely to inherent quantum behavior, as SA and our Random control classifier performed similar to, if not better than, quantum annealing as implemented by a D-Wave device on two of the three fractional training dataset comparisons. By randomly generating bit strings and sorting them by their Ising energy, we achieved classification accuracies nearly equal to standard ML and, in some cases, better than both quantum and SA. The comparable performances of our random control strategy and D-Wave and SA are due to a distinction between the objective function for the Ising-type approaches, which is an approximation for the negative log likelihood, and the performance metrics presented (accuracy, balanced accuracy, F1 score, AUC). While we describe this discrepancy in more detail in Figure S6 and Note S1, we found the overall classification performance of the random classifier to be a direct indication of the utility of formulating a classification problem as an Ising Hamiltonian. In this study, the advantage of using an Ising problem became even more apparent by training classifiers on a relatively smaller amount of training data, as we witnessed with the LumA versus LumB and ERpos versus ERneg breast cancer comparisons. For example, Field, which is an almost trivial algorithm after formulating the Ising problem, performed extremely well from 95% to 20% of the original training data for this breast cancer comparison.

The relative advantage of annealing-based Ising approaches over standard ML approaches when trained with relatively small amounts of data may be attributed to the discrete weights returned for the Ising-type methods. On one hand, discrete weights rendered with Ising-type methods control for statistical shrinkage better than statistical optimization parameters of standard ML approaches. This generalizability issue has plagued the ML field for decades. On the other hand, binary weights limit the informativeness of the standard classifiers; with larger amounts of training data, the Ising-type methods slightly underperformed standard ML approaches. These findings point to the potential application of a new class of algorithms, as simple heuristic models with discrete weights may perform better in situations of limited training data, which is often the case in clinical trials and drug efficacy studies. The relative advantageous trend of enhanced classification performance for Ising-type methods on small amounts of training data is true even when using gene-level features; Figure S7 shows balanced accuracies for LumA versus LumB and ERpos versus ERneg breast cancer comparisons relative to the top 44 genes from PC1 on incrementally smaller amounts of training data. Interestingly, the gene-level RBM underperforms all other Ising-type methods at all but the smallest fraction of training data. Moreover, Figure S8 shows statistically enhanced control of overfitting for Ising-type methods, especially at low fractions, on both LumA versus LumB and ERpos versus ERneg comparisons.

Although RBMs are formulated quite different from the annealing-based Ising models, in that RBMs are unsupervised and iteratively update model parameters, whereas the annealing-based approaches explored here are supervised with fixed h’s and J’s, the overall trends in classification performance for all these Ising models are quite similar. When using very small amounts of training data, RBMs also seem to perform much better than the standard ML methods, although their performance is noticeably better on the PCs than on the gene-level features. This may be attributed, in part, to distinct underlying data distributions; different algorithms do better on certain types of data. For example, Field, which performed quite well for the LumA versus LumB comparison on the PC-level data, performed poorly on the gene-level data, while RF performed better at the gene-level than the PC-level data. Differences in data type notwithstanding, the reason RBMs are performing well with small amounts of data may also be the binary nature of the hidden units. Although there are no “weights” to be learned, as there are with the annealing-based approaches, the hidden units for the RBMs were binarized during training; it is possible that this intentional sacrifice of precision leads to less overfitting with relatively smaller amounts of data.

Inherent to all useful biological classifiers, we showed that all the Ising-type algorithms identified relevant molecular features in each cancer comparison. Like the standard ML approaches, these algorithms determined PC1 as the most informative feature for each dataset, from which we then proceeded to perform gene-level classification. Analysis of feature importance of the trained classifiers on the top 44 genes of PC1 for the LumA versus LumB comparison determined *RACGAP1*, a putative oncogene in breast cancer, to be associated with the highest averaged information ranking. This finding was supported via independent differential gene expression analysis, indicating that LumB tumors, a more aggressive molecular subtype of breast cancer, were associated with statistically significant, higher mRNA levels of *RACGAP1* than LumA tumors. Moreover, our semantic search of full-text records available in the PMC database found that 43 of these top 44 genes have been previously implicated in breast cancer. While our results support previously published findings, it is possible that more sophisticated dimensionality-reduction techniques, such as multi-omics factor analysis,^39^ could be used to provide fresh insights into the mechanisms of disease. The effect of such techniques on the relative performance of both standard and Ising-type ML methods is worthy of further study.

While we achieved comparable classification performance on all binomial comparisons assessed in this study, it is important to note that our Ising-type approaches did not perform as well as standard ML on a large multiclass, six-cancer dataset. This observation is most likely related to the relatively larger training dataset used for this multiclass comparison, as the six-cancer dataset comprised approximately 12 times the amount of data relative to the LumA versus LumB dataset. As we showed by reducing the amount of training data for the LumA versus LumB, the ERpos versus ERneg, and the six-cancer multiclass comparisons, the Ising-type approaches performed well with relatively smaller amounts of data but did not statistically improve with incremental increases. Another explanation for the decreased performance of the Ising-type approaches may be related to the fact that the number of approximations used to formulate the classification problem as an Ising Hamiltonian depends on the number of classes (see supplemental experimental procedures, Equation 15). The approximation may be valid for binomial comparisons but could break down with multiclass experimental designs. In contrast, the RBMs, which are not formulated based on the same approximations, perform significantly better than the annealing-based Ising models.

Although practical quantum computing architectures are still in development, the demonstration of classification performances of Ising-type approaches that are comparable to standard ML methods on high-dimensional, multi-omics human cancer datasets is encouraging. Our survey of ML classifiers has uncovered a class of algorithms that perform better than standard methods on limited biomedical data: Ising-type methods with discrete weights. This advantage for small experimental designs is particularly useful in medicine, where large datasets may be prohibitively expensive to obtain, or in the study of rare diseases. As technology improves and new algorithms are introduced, we are cautiously optimistic that these unconventional classification algorithms will afford unique insights and drive the discovery of novel approaches for solving complex biological problems.

## Experimental procedures

### Resource availability

#### Lead contact

Tom Chittenden may be contacted for additional information (email:tom.chittenden@genuitysci.com).

#### Materials availability

No new materials were generated in this study.

#### Data and code availability

The processed data that supports the findings of the study is available at Mendeley Data: https://doi.org/10.17632/thjjpv3df3. The code that supports the findings of this study is available via https://github.com/Genuity-Science/unconventionalML.

### Methods

#### Dataset and dimensionality reduction

Genomic data from TCGA were retrieved, preprocessed, and normalized. An overview of our data pipeline is depicted in Figure 1. Briefly, we retrieved whole-exome sequencing, RNA-seq, miRNA sequencing, DNA methylation array, and genotyping array data for five human cancer binomial classifications (breast cancer versus normal, ERpos versus ERneg breast cancers, LumA versus LumB breast cancers, kidney renal clear cell versus papillary cell carcinoma, and LUAD versus squamous cell carcinoma), as well as a six-cancer multiclass classification, which included breast, colorectal, lung, kidney, brain, and liver cancer types. Data were retrieved from either the Genome Data Commons data portal (https://portal.gdc.cancer.gov/, data release 4.0) or cBioportal (http://www.cbioportal.org/).^40^^,^^41^ All five data types (mRNA, miRNA, CNV, DNA methylation, and somatic tumor variants) were preprocessed independently (see supplemental experimental procedures) and then concatenated into a single data matrix.

We derived classification performance via 100 random, approximately class-balanced partitions of training (80%) and test/validation (20%) data. Each feature was standardized to zero mean and unit variance (*Z* score) based on the training data. The same training mean and standard deviation was then applied to the corresponding test data. Furthermore, given that the data comprised more than 79,000 molecular features, dimensionality reduction was conducted in order to make comparisons with existing quantum hardware. As such, we performed PCA on each random, balanced partition of the training data, retaining the top 44 PCs for the binomial datasets and 13 PCs for the six-cancer dataset. The test data were then projected onto the PCs defined by the corresponding training data. The number of PCs was chosen based on the largest number of features that could be accommodated on existing quantum annealing hardware (see the section below on formulating the classification problem as an Ising model). Hyperparameters were selected using cross-validation on the training data (see supplemental experimental procedures for more information about which hyperparameters were chosen).

#### Quantum annealing

We explored the use of quantum annealing with processors made by D-Wave Systems, Inc.^11^^,^^12^ (see the supplemental experimental procedures for a brief review of quantum annealing). Results for the binomial datasets were obtained by running the D-Wave 2X (DW2X) processor installed at the Information Sciences Institute (ISI) of the University of Southern California, and results for the six-cancer dataset were run on the DW2000Q located in Burnaby, British Columbia, Canada. The problem Hamiltonians that are used for D-Wave can be described as Ising spin models with tunable parameters.^11^ The Ising model assumes a graph G=(V,E) composed of a set of vertices, V, and edges, E. Each of the spins is a binary variable located at a unique vertex. The spins are represented by N superconducting flux qubits, and G is the so-called Chimera graph (see Figure S9). For the DW2X, *N =* 1,098, and for the DW2000Q, *N =* 2,038. The problem (or Ising) Hamiltonian for this system can be written as:

(Equation 1) $$H_{P}=\underset{i\in V}{\sum }h_{i}{\text{σ}}_{i}^{z}+\underset{\left(i,j\right)\in E}{\sum }J_{i,j}{\text{σ}}_{i}^{z}{\text{σ}}_{j}^{z}\text{,}$$

where the local fields $\left\{h_{i}\right\}$ and couplings $\left\{J_{ij}\right\}$ define a problem instance and are programmable on the DW2X to within a few percent Gaussian distributed error. The $\left\{{\text{σ}}_{i}^{z}\right\}$ represent both binary variables taking on values ±1 and the Pauli *z* matrices. Given a spin configuration $\left\{{\text{σ}}_{i}^{z}\right\},\;H_{p}$ is the total energy of the system. Problems submitted to D-Wave are automatically scaled so that all $h_{i}$ and $J_{ij}$ values lie between −1 and 1. The initial Hamiltonian $H_{B}=\underset{i}{\sum }{\text{σ}}_{i}^{x}$ is a transverse magnetic field where ${\text{σ}}_{i}^{x}$ is the Pauli *x* matrix acting on qubit i. During an anneal, the magnitude of $H_{B}$ is gradually reduced to zero, while the magnitude of $H_{p}$ is slowly increased from zero. After each anneal D-Wave returns a set of spin values $\left\{{\text{σ}}_{i}^{z}=\pm 1\right\}$ that attempts to minimize the energy given by Equation 1 (a lower energy indicates better optimization). Note, however, that for our purposes we are not strictly using D-Wave as an optimizer. In the supplemental experimental procedures, we describe our procedure to make use of the fact that higher-energy solutions may still contain some meaningful information and use them to improve performance.

For the results in the main text, we set the annealing time at 5 μs and repeated the anneal 1,000 times, which returns 1,000 spin configurations. We selected the 20 spin configurations with the lowest Ising energy and ran some quick classical postprocessing to average the lowest Ising energy spin configurations if they improved the objective function on the training data. See the supplemental experimental procedures for a more detailed description of other hyperparameters and Figures S10–S12 and Note S2 for the effect of using a larger number of spin configurations.

#### Simulating annealing

Similar to quantum annealing, SA accepts problems formulated as an Ising problem, as defined in Equation 1, and returns binary variables. For this work we used the implementation of Isakov et al.^42^ There are several important parameters that affect SA's overall performance: the number of sweeps, the type of schedule (linear or exponential in the inverse temperature), and the initial and final temperatures. For our purposes, we fixed the number of sweeps (which is analogous to the annealing time of quantum annealing) to 1,000 and selected a linear schedule with an inverse initial temperature of 0.01. We treated the final inverse temperature as a tunable hyperparameter with values in the set {0.03,0.1,0.3,1,3} and repeated the anneal 1,000 times. Results in the main text are given for the final inverse temperature that yielded the best performance during cross-validation. We used the same classical postprocessing procedure that was used with D-Wave to combine 20 spin configurations with the lowest energy, not just the one that returned the lowest Ising energy.

#### Field

As another approach to explore the usefulness of the formulating the classification task as an Ising problem, and to check the role played by the couplings (the J’s), we implemented a very simple algorithm that takes into account only the values of the local fields (the h’s) in Equation 1. Once h has been determined based on the training data, we chose the weights to be the opposite sign of the fields, i.e., ${\text{σ}}_{i}^{\text{field}}=-h_{i}$. This amounts to a (trivial) analytical solution of the optimization of Equation 1 without any J’s.

#### Random

As a sanity check, we generated random solutions to Equation 1. For each spin we picked a random number uniformly distributed in the interval [0,1). Values below 0.5 were set to −1 and those above 0.5 were set to 1, thereby generating spin configurations the same as those returned by D-Wave and SA. We then sorted the spin configurations according to their Ising energy, given by Equation 1. As with D-Wave and SA, we generated 1,000 such random spin configurations and used the same classical postprocessing procedure to combine the 20 spin configurations with the lowest energy to a final set of weights.

#### RBM

RBMs are a class of bipartite unsupervised energy-based models that consist of a visible layer (the data one would like to model) and a hidden layer. Introducing a hidden layer allows one to model more complicated probability distributions. An RBM defines a probability distribution through an energy function as Pr(x)=exp[−H(x)]/Z, where $Z=\underset{x}{\sum }\left[-H\left(x\right)\right]$. The energy function for RBMs can be written as an Ising Hamiltonian, although the goal of learning is to update the h’s and J’s such that the probability distribution most closely resembles the input data distribution; in contrast, the h’s and J’s for the models above are fixed, given the training data.

To use RBMs for classification, we used the approach described in Larochelle et al.,^43^ where class labels were added to the visible layer and the RBMs learned to model a distribution over both the “data” units and the “label” units, to adapt the R deepnet package. After training, the probability of each class was calculated by setting the corresponding label unit to 1 and all other label units to 0. The RBMs were trained with contrastive divergence using a k=1 and a batch size of 32.

#### Formulating a multiclass classification problem on a quantum annealer

We show how to arrive at a simple Ising formulation to model a multiclass classification problem with K unique class labels. Assume we have a dataset of N training examples, $D={\left\{\left(x_{i},y_{i}\right)\right\}}_{i=1}^{N}$, where $x_{i}$ is the ith data vector of M features and $y_{i}$ is the corresponding class of the ith data vector (i.e., $y_{i}$ can take one of the K class labels). A simple way to arrive at probabilities for a multiclass classification problem is to use the softmax function. We can define the probability of each class as:

(Equation 2) $$Pr\left(y_{i}=k\right)=\frac{\exp\;w_{k}^{⊺}x_{i}}{\sum_{k=1}^{K}\exp\;w_{k}^{⊺}x_{i}}\text{,}$$

where $w_{k}$ are the weights corresponding to the kth class that we would like to learn (in other words, we define a set of weights for each class). However, since we are generating a probability of each class, we can reduce the set of weights we have to train from K to K−1 and define the first K−1 probabilities as:

(Equation 3) $$Pr\left(y_{i}=k\right)=\frac{\exp\;w_{k}^{⊺}x_{i}}{1+\sum_{k=1}^{K-1}\exp\;w_{k}^{⊺}x_{i}}\text{,}$$

with the probability of the Kth class as:

(Equation 4) $$Pr\left(y_{i}=K\right)=\frac{1}{1+\sum_{k=1}^{K-1}\;\exp\;w_{k}^{⊺}x_{i}}\text{.}$$

The goal of training is to find the weights that maximize the probability given the classes in the dataset or, equivalently, to minimize the negative log likelihood. Once the weights are found, inference is straightforward; probabilities for each class are generated and we assign the predicted label based on the class with the highest predicted probability. We can express the negative log likelihood as follows:

(Equation 5) $$L=-\log\underset{i}{\prod }Pr\left(y_{i}\right)$$

(Equation 6) $$=-\sum_{i}\log\;Pr\left(y_{i}\right)\text{,}$$

where the probability selected corresponds to the actual class of the label. If the actual class has the highest predicted probability for all data samples, the negative log likelihood will be minimized. In other words, the further away from 1 the predicted probability of the real class is, the greater the contribution to the negative log likelihood; if the algorithm were able to correctly assign a class to each training example with probability 1, the negative log likelihood would be 0.

Taking a second-order Taylor approximation around the argument of the exponential equal to 0, we eventually arrive at the following expression for the negative log likelihood (see supplemental experimental procedures for a more complete derivation and additional technical concerns):

(Equation 7) $$L\approx \sum_{k=1}^{K-1}w_{k}^{⊺}\left(b_{k}+h\right)+\sum_{k=1}^{K-1}w_{k}^{⊺}J^{\prime }w_{k}-\sum_{k=1}^{K-1}\sum_{j\neq k}w_{j}^{⊺}J^{\prime \prime }w_{k},$$

where

(Equation 8) $$b_{k}=\sum_{i:y_{i}=k}-x_{i},h=\frac{1}{K}\sum_{i}x_{i},$$

(Equation 9) $$J^{\prime }=\frac{K-1}{2K^{2}}\sum_{i}x_{i}x_{i}^{⊺},J^{\prime \prime }=\frac{1}{2K^{2}}\sum_{i}x_{i}x_{i}^{⊺}.$$

In general, this formulation requires arbitrary interweight couplings (i.e., $J^{\prime \prime }$-- couplings between $w_{k}$ and $w_{j}$, where $w_{k}$ and $w_{j}$ represent the vector of weights for classes k and j) and intraweight couplings ($J^{\prime }$-- couplings between $w_{k,n}$ and $w_{k,m}$, where n and m are the indices of the weights assigned to the nth and mth features for the vector of weights for the kth class). This imposes constraints on the number of classes and number of features that can be run on a particular hardware graph. For a dataset with M features and K classes, this approach requires M×(K−1) logical variables, and in general there must be. Due to restrictions on the number of qubits and the limited graph connectivity (see Figure S9), an *embedding*, by which edges in the graph are contracted to give a graph with fewer vertices but a higher degree, is generated (see supplemental experimental procedures for more details). For the D-Wave 2000Q, the largest complete graph that can be embedded^44^ consists of 66 logical variables; i.e., M×(K−1) must be at most 66. For our purposes, we chose K=6 cancer types, which limits the number of features we can use to 13. The largest complete graph that can be embedded onto the DW2X processor at ISI consists of 45 logical variables, so for the binomial datasets we chose a total of 44 features.

## Supplemental information

Document S1. Supplemental experimental procedures, Figures S1–S12, Tables S1–S4 and S7, and Notes S1 and S2

Table S5 LumAB gene ranking: Ranking of algorithms of genes from PC1 for luminal A versus luminal B comparison

Table S6 LumAB GOseq: GOseq analysis of top 44 genes from PC1 for luminal A versus luminal B comparison

Document S2. Article plus supplemental information

## Footnotes

Supplemental information can be found online at https://doi.org/10.1016/j.patter.2021.100246.
