# Supplementary material for: Quantum processor-inspired machine learning in the biomedical sciences
Source: Patterns (N Y). 2021 Apr 28;2(6):100246. doi: 10.1016/j.patter.2021.100246 (PMC8212142; doi:10.1016/j.patter.2021.100246)
Supplement: Document S1. Supplemental experimental procedures, Figures S1–S12, Tables S1–S4 and S7, and Notes S1 and S2 [file mmc1.pdf]

Patterns, Volume *2*

Supplemental information

Quantum processor-inspired machine learning in the biomedical sciences

Richard Y. Li, Sharvari Gujja, Sweta R. Bajaj, Omar E. Gamel, Nicholas Cilfone, Jeffrey R. Gulcher, Daniel A. Lidar, and Thomas W. Chittenden

Supplemental Items

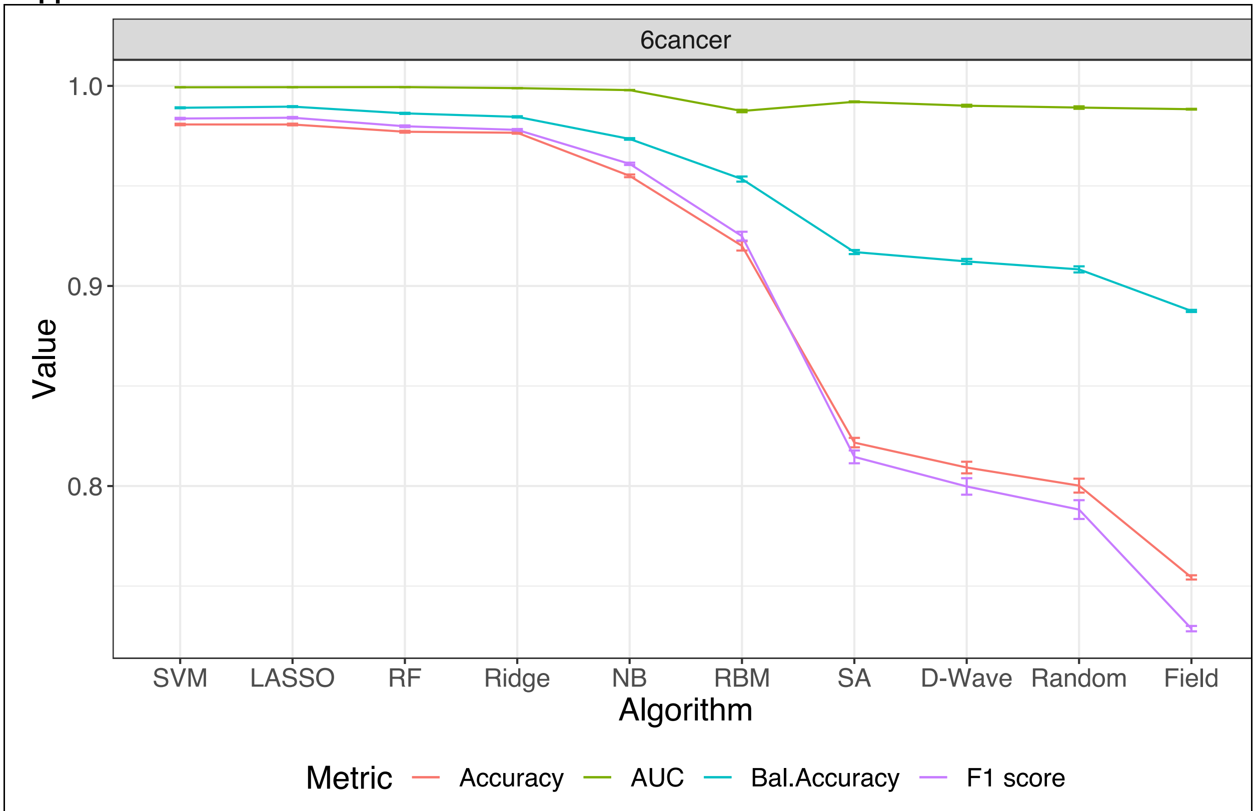

Figure S1: Related to Figure 1. Assessment of algorithm performance for multi-class classification of six TCGA human cancer types: breast, colorectal, lung, kidney, brain, and liver. Classification performance metrics were averaged for the 100 unique test sets for each model (see methods). Performance metrics: Accuracy (red), AUC (green), Balanced Accuracy (blue), F1 score (purple). Data are presented as mean ± SEM.

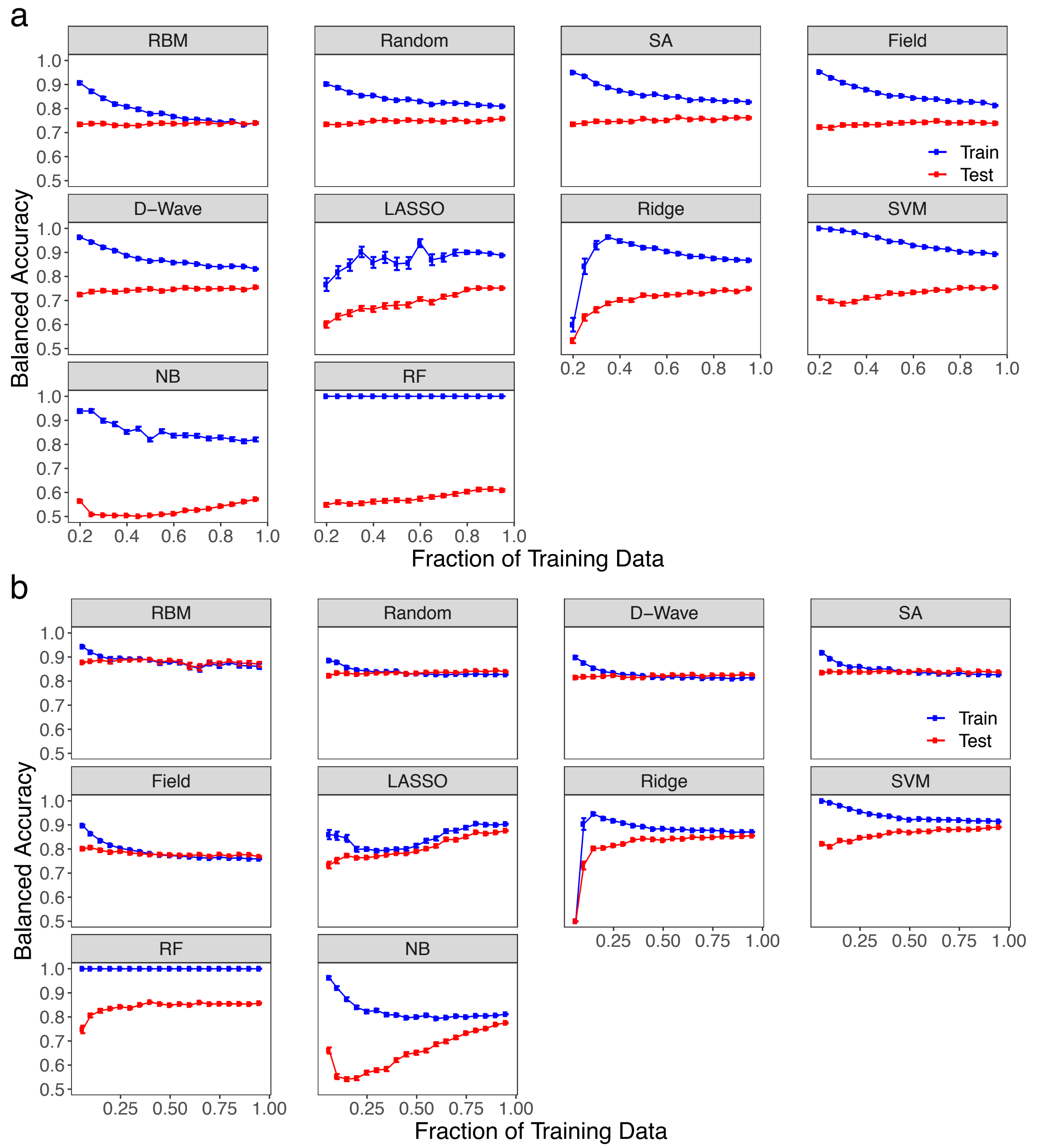

Figure S2: Related to Figure 3. (a) Train and test balanced accuracies for the LumA vs. LumB binomial classification dataset. Order of the panels (left to right, top to bottom) is based on the average difference between performance on the train and the test across the range of data fractions shown for all nine classifiers assessed. (b) Train and test balanced accuracies for the ERpos vs. ERneg binomial classification dataset. Ordering of panels is the same as is used for (a).

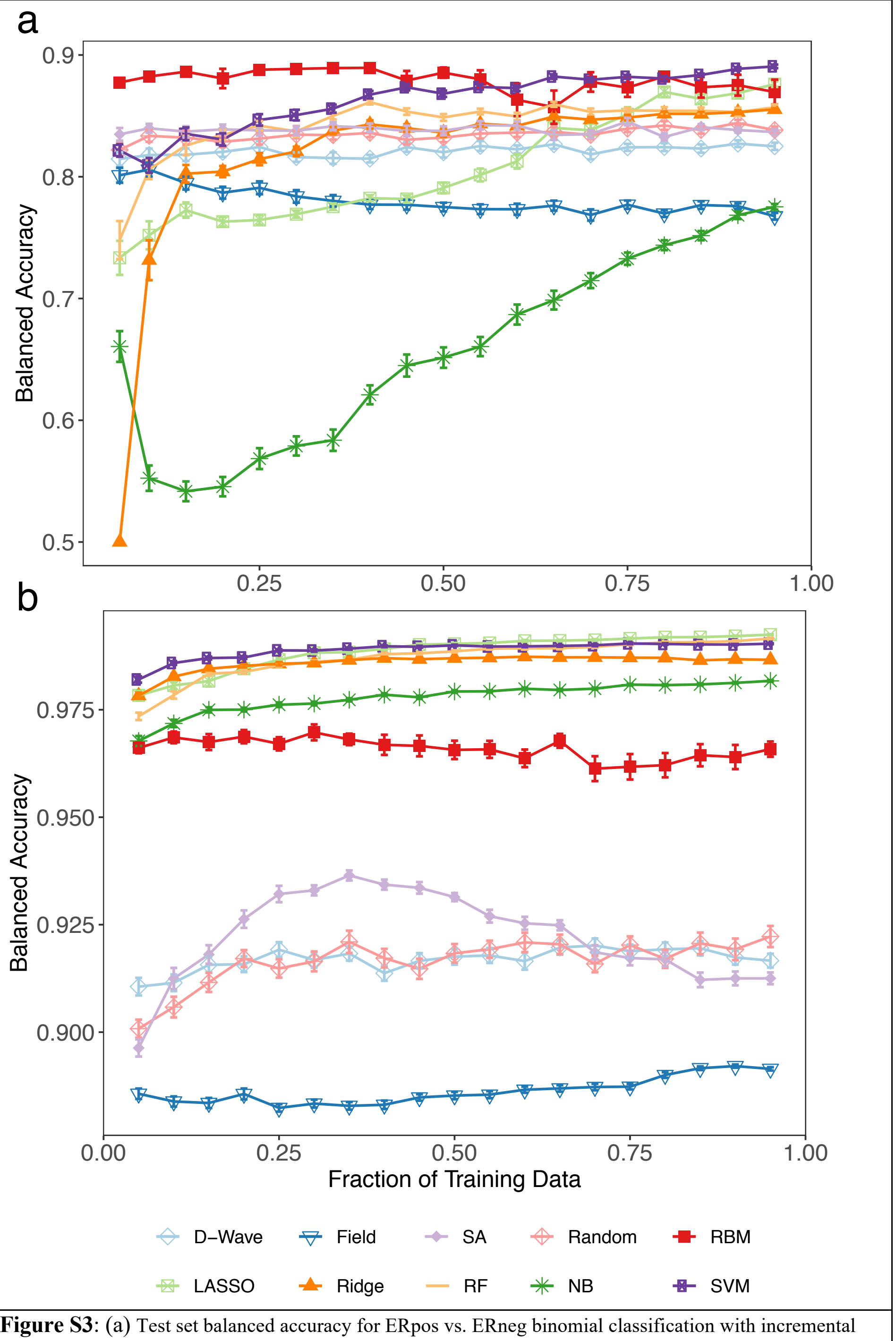

Figure S3: (a) Test set balanced accuracy for ERpos vs. ERneg binomial classification with incremental decreases from 95% to 10% of original training set. (b) Test set balanced accuracy for the six-cancer classification with incremental decreases from 95% to 5% of original training set. The six TCGA human cancer types assessed: breast, colorectal, lung, kidney, brain, and liver. Averaged balanced accuracies were calculated for 50 independent training sets at each designated fraction of original training data. Data are presented as mean ± SEM.

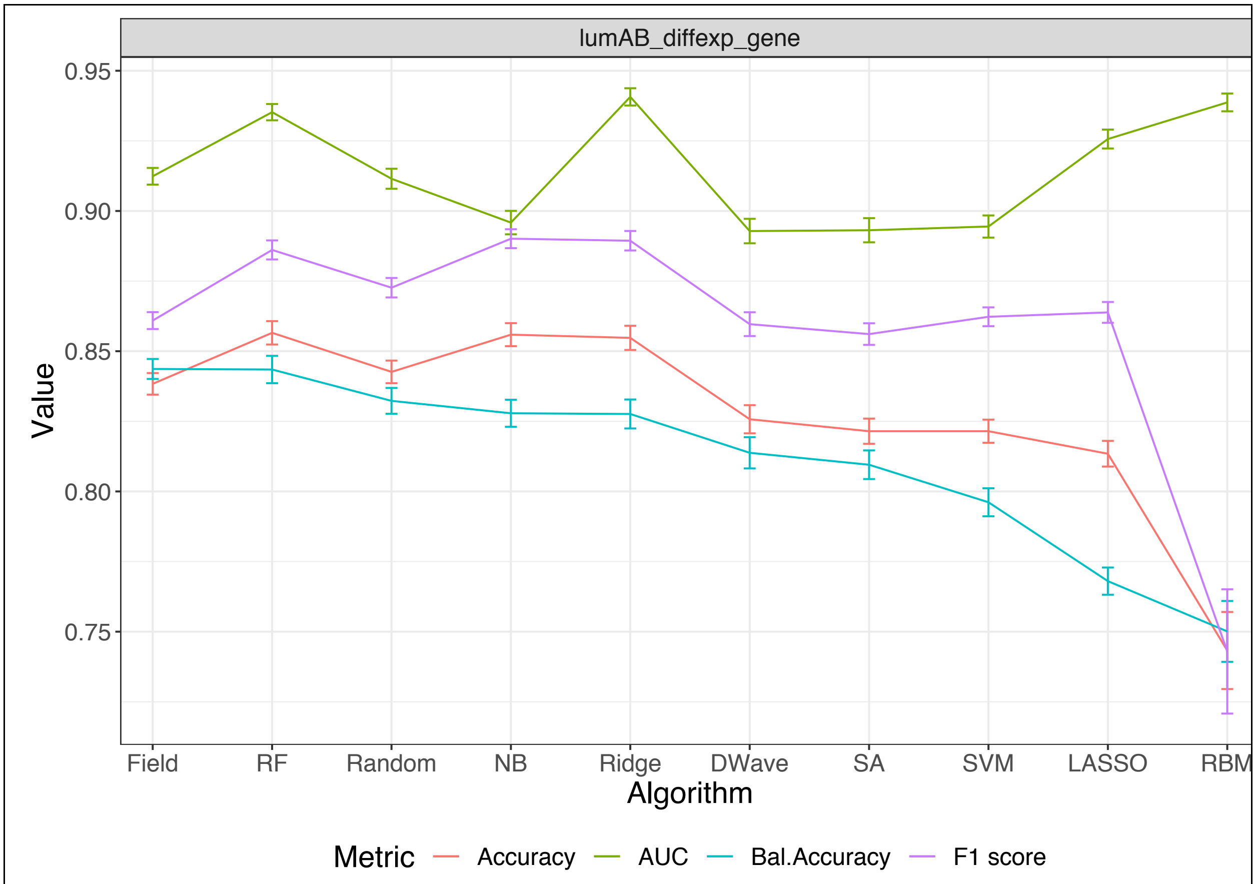

Figure S4: Related to Figure 4. Assessment of algorithm performance for Luminal A versus Luminal B breast cancers based on most differentially expressed genes. Classification performance metrics were averaged for the 100 unique test sets for each model (see methods). Performance metrics: Accuracy (red), AUC (green), Balanced Accuracy (blue), F1 score (purple). Data are presented as mean ± SEM.

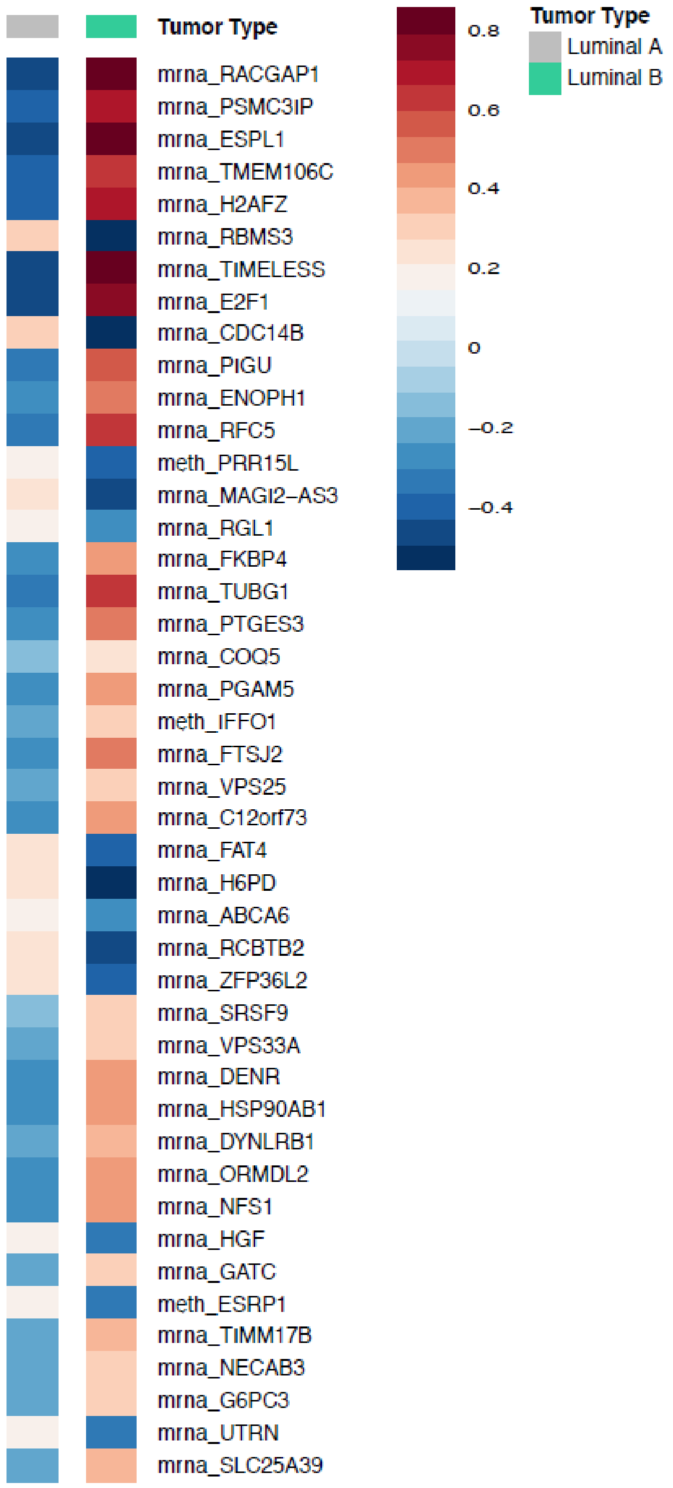

Figure S5: Related to Figure 4. Rank-ordered **h**eatmap of the averaged state for each of the top 44 genes for the LumA vs. LumB comparison.

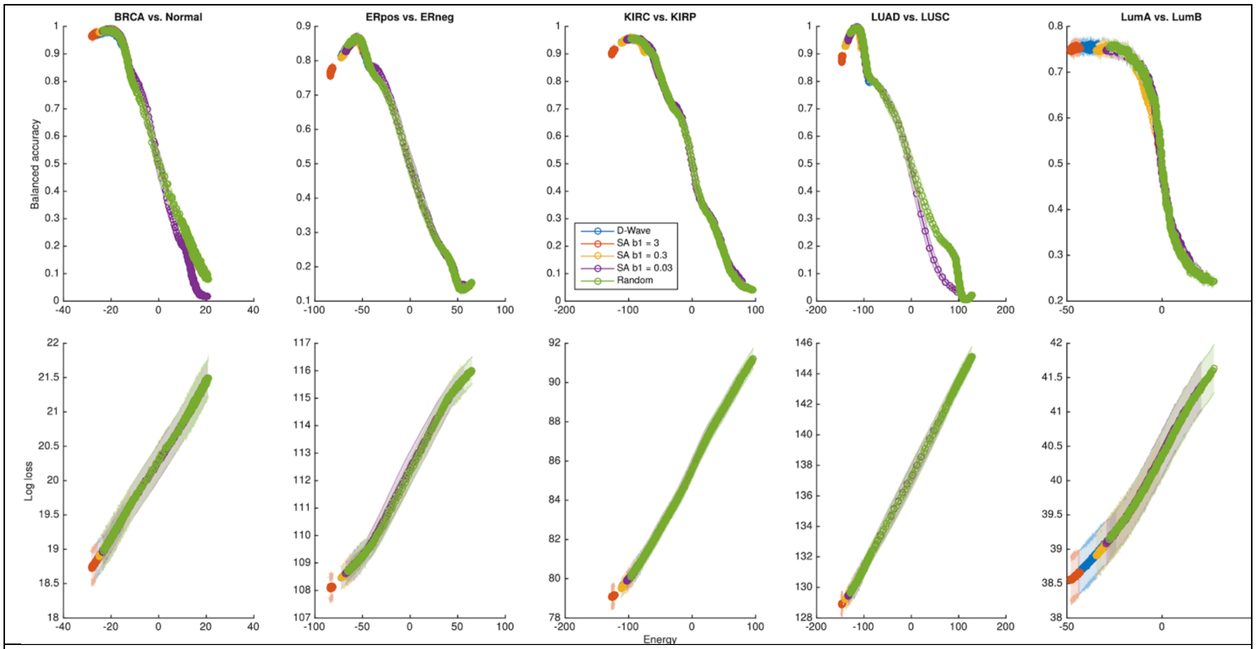

Figure S6: Evaluation of the performance of D-Wave, SA at various final inverse temperatures (b1 in the Legend), and Random, when using a sliding window of energies. Average balanced accuracy (top) and the average log loss (bottom) across the 100 cuts on the test dataset versus the average Ising energy of the post-processed weights. The shaded region represents 2 standard deviations.

Note S1: Related to Figure S6.
As a way to gain some insight into the machine learning performance with respect to Ising energy, we considered the effect of using higher-energy excited-state solutions; i.e., the solutions that have a higher energy according to the Ising Hamiltonian defined in Eq. (19). To do so, we first generated 1000 sets of weights for each method (D-Wave, SA, and Random). The weights were then sorted according to their Ising energy. Next, we used a sliding window of energies; i.e., we applied our post-processing averaging procedure to 20 solutions at a time starting from the 20 set of weights with the lowest Ising energy, then applying the averaging procedure to the 20 set of weights with the next lowest Ising energy and so on. This procedure was repeated for all 100 cuts of the data for each dataset, and the results for the balanced accuracy and negative log-likelihood are presented in Supplemental Figure S6.

The top row of the Supplemental Figure S6 shows a maximum in the balanced accuracy versus the Ising energy for most of the datasets. This maximum indicates the presence of a mismatch between the optimized objective function (the Ising energy) and the performance metric (the balanced accuracy); i.e., some solutions that perform worse in terms of the Ising energy perform better in terms of the balanced accuracy. This is in part due to the nature of the logistic loss, which is somewhat sensitive to outliers. To calculate the balanced accuracy, we must first threshold the predicted probabilities, assigning classes based on whether the probability for that class is greater than 0.5. Further, we note that by tuning the final temperature of SA, we are able to control the weights found to lie within a particular energy range; higher final temperature (smaller b1) shift the energies to the right. Note that while it is possible to bring SA to find higher energy solutions by increasing the final temperature, there is no single parameter we can use to decrease the temperature of the weights found by the random method, other than increasing the number of weights we randomly generate (for D-Wave we can essentially control the temperature by scaling the h's and J's). It seems reasonable to expect that with a growing number of features, Random will become less and less likely to find solutions low enough in energy to be useful; i.e., we may expect the solutions to lie to the right of the maximum. D-Wave and SA are generally to the left of the maximum and we can effectively "raise the temperature" such that they find solutions that are near the maximum.

The bottom row of Supplemental Figure S6 shows the averaged negative log-likelihood across the 100 cuts of the data versus the average Ising energy. Though not always linear, there is a clear correlation between the Ising energy and the log-loss. This indicates that the approximations we used to generate the Ising problem from the log-loss, though perhaps not perfect, are good enough that we see excellent correlations between the two. Had the approximation not been valid, we would expect to see very poor correlation, or no correlation at all between the two.

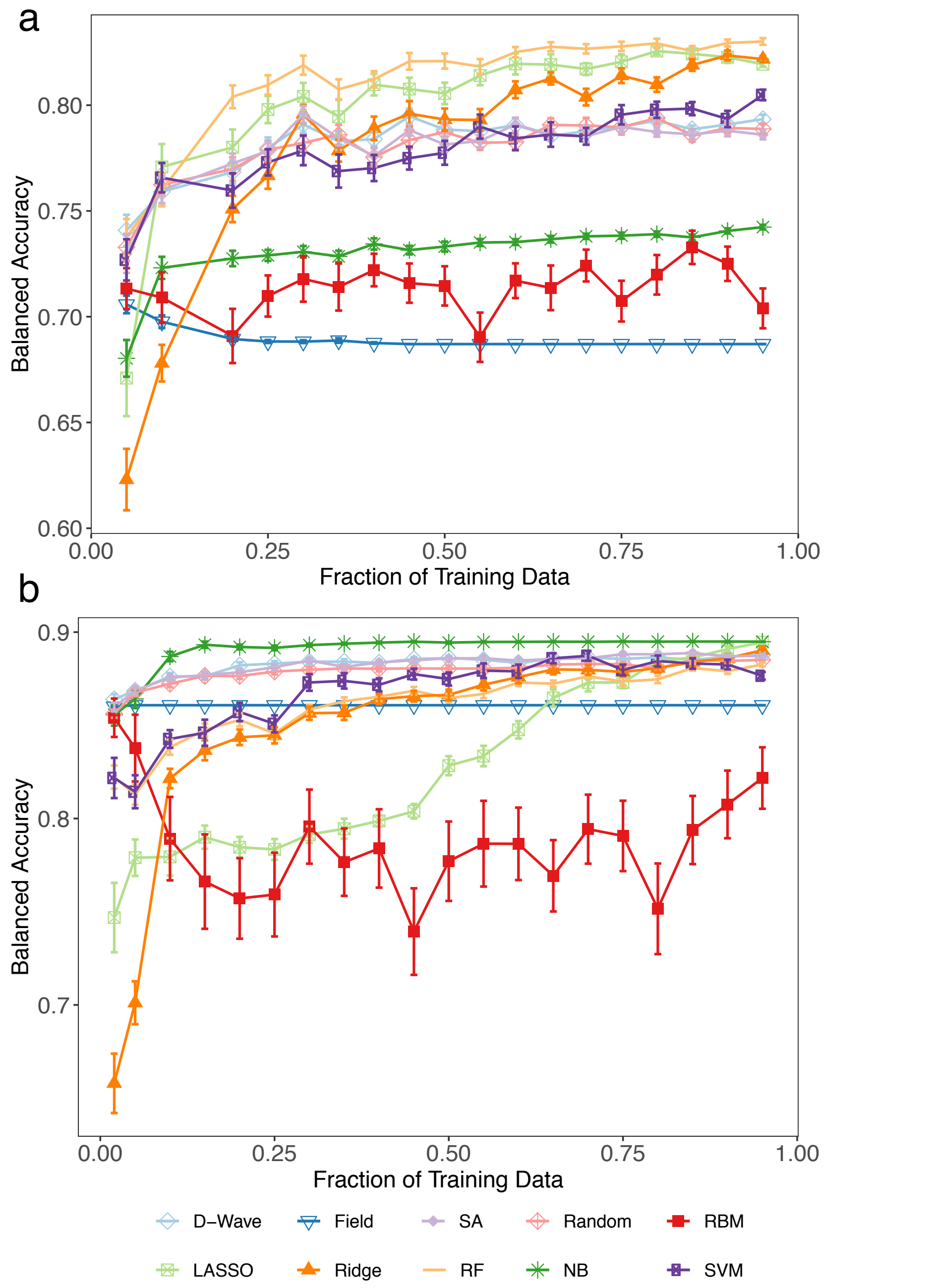

Figure S7: (a) Test set balanced accuracy for LumA vs. LumB binomial classification on top 44 genes from PC1 with incremental decreases from 95% to 5% of original training set. (b) Test set balanced accuracy for ERpos vs. ERneg binomial classification on top 44 genes from PC1 with incremental decreases from 95% to 2% of original training set. Averaged balanced accuracies were calculated for 50 independent training sets at each designated fraction of original training data. Data are presented as mean ± SEM.

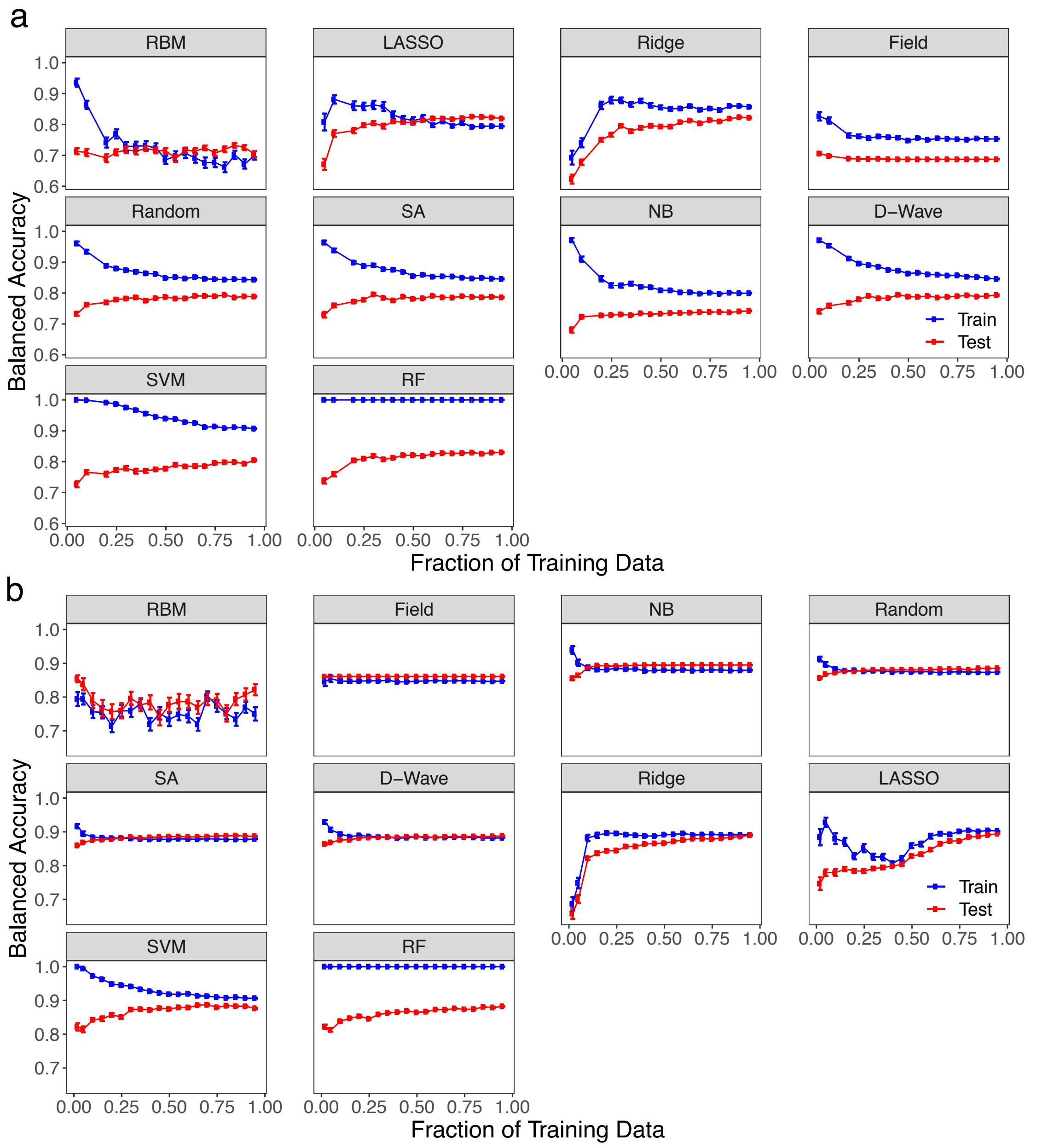

Figure S8: (a) Train and test balanced accuracies for the LumA vs. LumB binomial classification dataset with genes from PC1. Order of the panels (left to right, top to bottom) is based on the average difference between performance on the train and the test across the range of data fractions shown for all nine classifiers assessed. (b) Train and test balanced accuracies for the ERpos vs. ERneg binomial classification dataset with genes from PC1. Ordering of panels is the same as is used for (a).

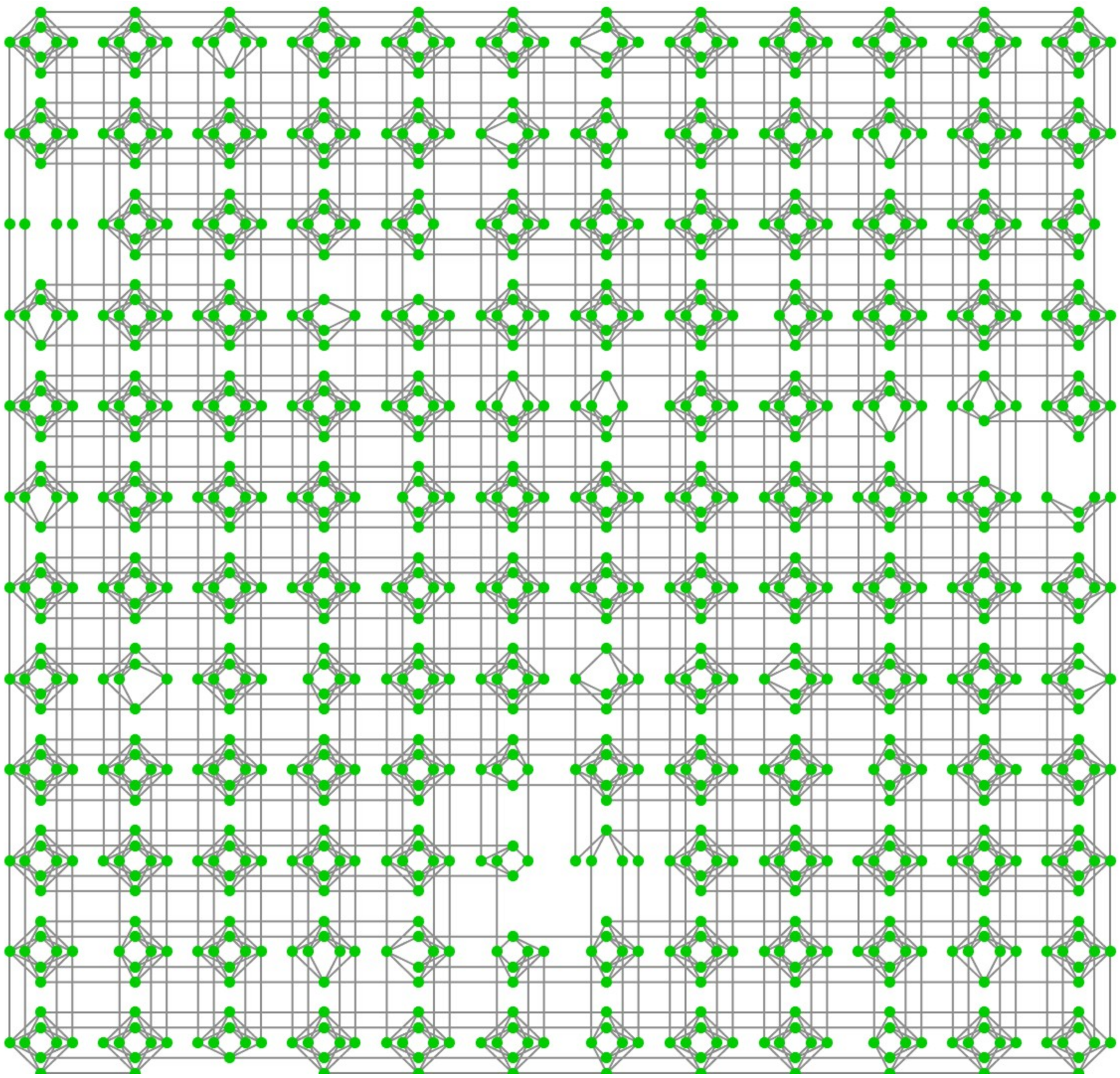

Figure S9: Schematic representation of the “Chimera” hardware graph of the DW2X housed at the Information Sciences Institute at USC, used in this work. Green circles represent active qubits, inactive qubits are omitted, lines represent couplings between qubits. Each qubit can be coupled to a maximum of six other qubits.

Note S2: Related to Figures S10-S12

Because D-Wave, SA, and Random are all probabilistic, returning a distribution of solutions, finding weights with low Ising energy is somewhat dependent on the total number of solutions that are found. We note that the ability of the respective methods in finding low energy solutions differs; as seen in Supplemental Figure S6, with a 1000 solutions, Random does not find solutions that are as low in energy as D-Wave and SA; we might expect the need to randomly generate on the order of 2^{44} (recall that we used 44 PCs for the binomial datasets) solutions in order for Random to find the solutions that match the Ising energy of the solutions returned by D-Wave and SA.

In addition to the total number of solutions, performance is also dependent on the number of solutions we include in our post-processing procedure. Our post-processing procedure is designed to monotonically improve performance on the training datasets, and thus we might expect including more solutions to improve performance. We did not consider using all solutions for several reasons: first, doing so increases the amount of time needed to generate final candidate solutions; second, by including many of

the solutions, we begin to somewhat lose the discrete nature of the weights and therefore some of the robustness associated with them; finally, monotonically improving performance on the training dataset may lead to overfitting, and therefore using a smaller number of solutions is somewhat analogous to an early-stopping regularization scheme.

In the main text and in Supplemental Figure S6, we selected 20 best performing solutions to include in our post-processing procedure. The choice of 1000 and 20 are somewhat arbitrary, so to systematically determine the effect of this number of solutions, we vary the total number of solutions from 1 to 1000 and the number of best performing solutions from 1 to 1000 for each of the five binomial datasets. As before, we used the same 100 cuts of the data described in the main text for each of the datasets. The average test balanced accuracy, average test logistic loss, and the average training negative Ising energy are shown in Supplemental Figures S10, S11, and S12, respectively. For each performance metric (i.e., balanced accuracy, logistic loss, and Ising energy), we chose the final inverse temperature for SA and the J_c for D-Wave (see Section on additional technical details of D-Wave) that gave the best performance of that measure.

Supplemental Figures S10-S12 all show that when using a very small number of total solutions, Random does worse than D-Wave and SA on all metrics. However, with as few as 50 total solutions and 5 best performing solutions, Random performs nearly the same as D-Wave and SA in terms of the balanced accuracy. Further increasing the total number of solutions and the number of solutions used in the post-processing procedure improves the balanced accuracy for D-Wave, SA, and Random. For estrogen receptor positive (ERpos) vs. estrogen receptor negative (ERneg) and lung adenocarcinoma (LUAD) vs. lung squamous cell carcinoma (LUSC), using all 1000 out of 1000 solutions gives the best performance, however for the other datasets, using a smaller number (around 20 or 50) of the total solutions gives nearly equal performance as using all 1000. For Random, using all 1000 solutions for the luminal A (LumA) vs. luminal B (LumB) breast cancers dataset is worse than using 50 solutions, indicating that for this dataset early stopping may help improve performance in terms of the balanced accuracy. Supplemental Figures S11 and S12 show that Random does not find solutions that are as low in Ising energy or with as low of a logistic loss as D-Wave and SA, confirming what was shown in Supplemental Figure S6.

These additional results show that even by exploring only a small subset of the total search space (1000 out of 2^{44} possible solutions), Random is able to give very good machine learning performance. Because the logistic loss is somewhat sensitive to outliers, finding “good” solutions with low (but not the lowest) Ising energy seem to give the best machine learning performance in terms of the balanced accuracy.

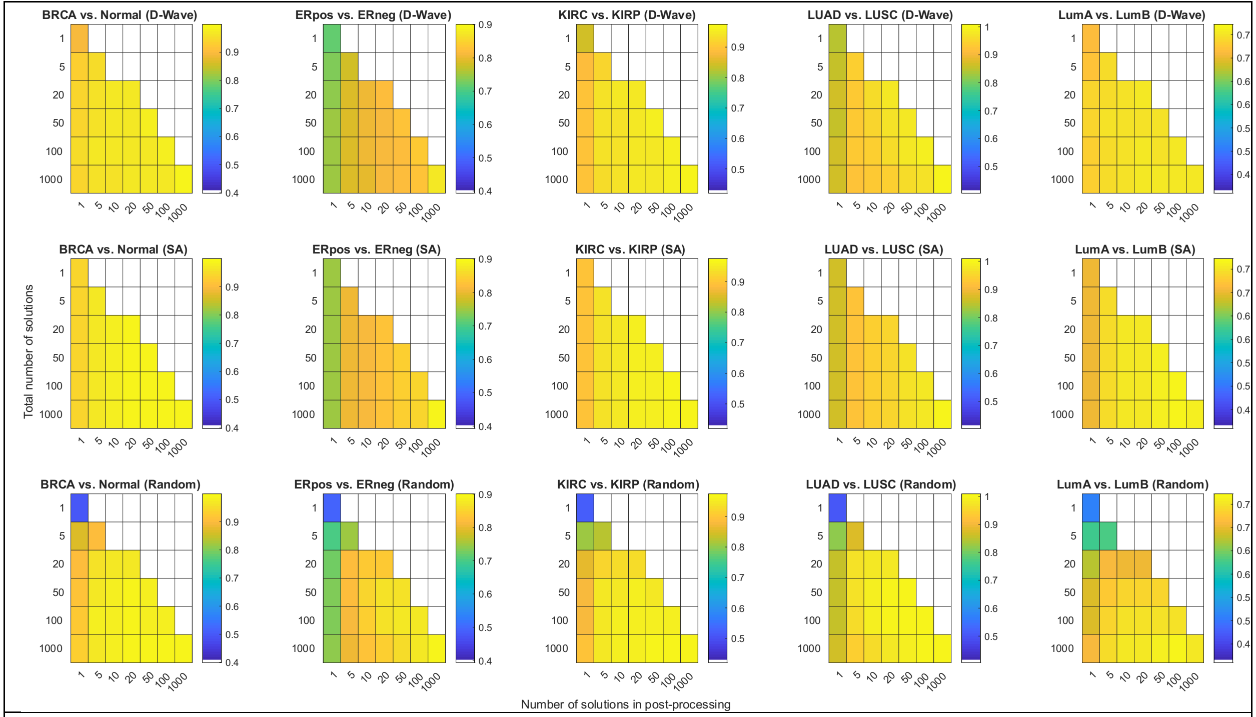

Figure S10: Heatmaps showing the effect of changing the total number of solutions (y-axis) and the number of solutions used in the post-processing procedure (x-axis) on the average balanced accuracy on the test datasets for the five binomial datasets. SA was run with a final inverse temperature of b1 = 0.03 and D-Wave was run with a J_c of 8.0. A higher balanced accuracy indicates better performance.

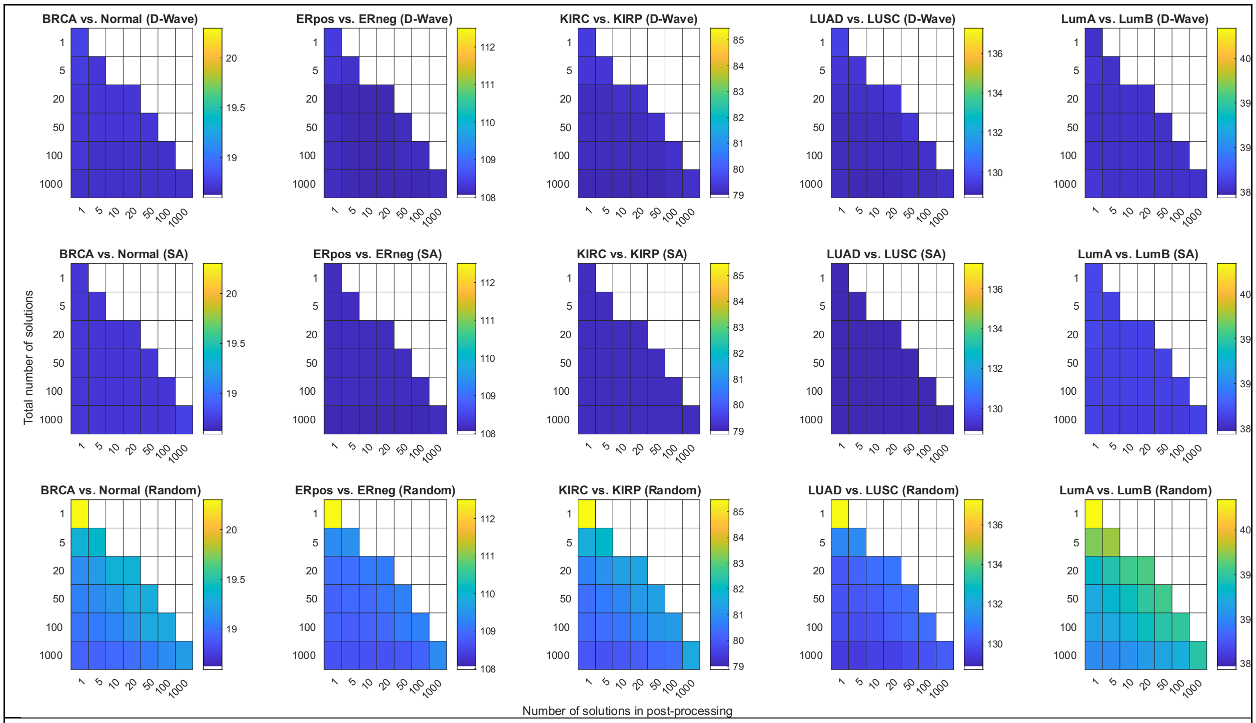

Figure S11: Heatmaps showing the effect of changing the total number of solutions (y-axis) and the number of solutions used in the post-processing procedure (x-axis) on the average logistic loss on the test datasets for the five binomial datasets. SA was run with a final inverse temperature of b1 = 3 and D-Wave was run with a J_c of 1.0. A lower logistic loss indicates better performance.

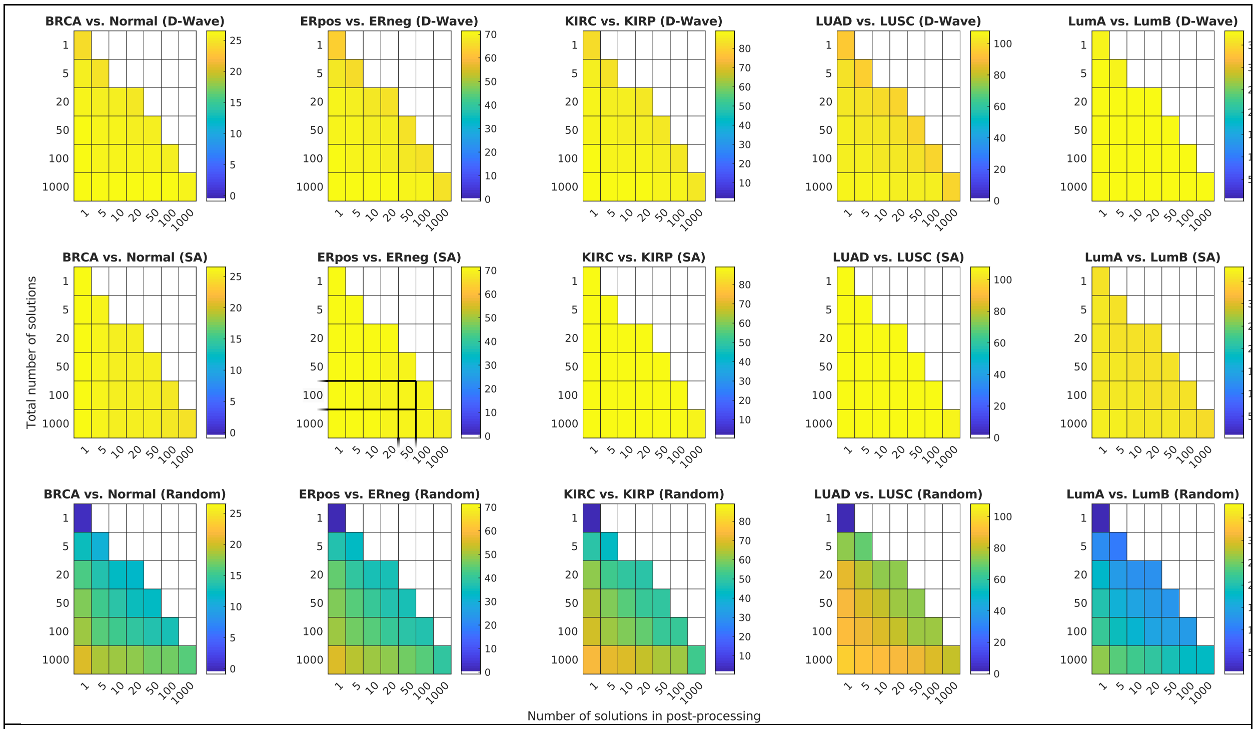

Figure S12: Heatmaps showing the effect of changing the total number of solutions (y-axis) and the number of solutions used in the post-processing procedure (x-axis) on the average negative Ising energy on the training datasets for the five binomial datasets. SA was run with a final inverse temperature of b1 = 3 and D-Wave was run with a J_c of 1.0. A lower Ising energy (higher negative Ising energy) indicates better performance.

Dataset	Training Set	Test Set	Ratio of classes
KIRC vs. KIRP	490	121	53:47
LUAD vs. LUSC	770	192	52:48
BRCA vs. Normal	118	28	50:50
ERpos vs. ERneg	768	191	77:23
LumA vs. LumB	250	61	64:36
Six Cancer Types (brca, coad, kidn, lgg, lihc, lung)	3192	795	25:14:15:13:9:24

Table S1: Datasets with the number of human tumor samples in the training and test sets. Kidney Renal Clear Cell Carcinoma (KIRC) vs. Kidney Renal Papillary Cell Carcinoma (KIRP); Lung Adenocarcinoma (LUAD) vs. Lung Squamous Cell Carcinoma (LUSC); Breast Invasive Carcinoma (BRCA) vs. matched normal tissue (normal); estrogen receptor positive (ERpos) vs. estrogen receptor negative (ERneg) breast cancers; and luminal A (LumA) vs. luminal B (LumB) breast cancers. Six human cancer types: breast, colorectal, lung, kidney, brain, and liver.

Dataset	LASSO	Ridge	SVM	RF	NB	DW	SA	Rand	Field	RBM
BRCA vs Normal	0.981 ± 0.002	0.982 ± 0.002	0.980 ± 0.003	0.989 ± 0.002	0.895 ± 0.006	0.974 ± 0.003	0.981 ± 0.003	0.982 ± 0.002	0.952 ± 0.004	0.981 ± 0.002
ER pos vs ER neg	0.921 ± 0.002	0.919 ± 0.002	0.928 ± 0.002	0.920 ± 0.002	0.875 ± 0.002	0.769 ± 0.003	0.785 ± 0.003	0.803 ± 0.003	0.714 ± 0.004	0.851 ± 0.007
KIRC vs KIRP	0.978 ± 0.001	0.976 ± 0.001	0.979 ± 0.001	0.945 ± 0.002	0.938 ± 0.002	0.946 ± 0.002	0.948 ± 0.002	0.955 ± 0.002	0.894 ± 0.003	0.930 ± 0.002
LUAD vs LUSC	0.9989 ± 0.0002	0.9999 ± 0.0001	1.0000 ± 0.0000	0.9956 ± 0.0003	0.9952 ± 0.0004	0.9482 ± 0.0017	0.9539 ± 0.0017	0.9752 ± 0.0013	0.8619 ± 0.0031	0.9969 ± 0.0003
Lum A vs Lum B	0.788 ± 0.005	0.781 ± 0.005	0.788 ± 0.005	0.722 ± 0.006	0.655 ± 0.006	0.747 ± 0.006	0.750 ± 0.006	0.747 ± 0.006	0.740 ± 0.006	0.721 ± 0.007
6 cancer	0.9807 ± 0.0004	0.9766 ± 0.0004	0.9807 ± 0.0004	0.9771 ± 0.0005	0.9550 ± 0.0007	0.8092 ± 0.0029	0.8217 ± 0.0024	0.8002 ± 0.0035	0.7543 ± 0.0011	0.9200 ± 0.0023

Table S2: Accuracies for five binary classification datasets and the one six-class cancer dataset used in this study. Data reported as mean ± SEM

Dataset	LASSO	Ridge	SVM	RF	NB	DW	SA	Rand	Field	RBM
BRCA vs Normal	0.9998 ± 0.0001	0.9998 ± 0.0001	0.9991 ± 0.0003	0.9977 ± 0.0007	0.9869 ± 0.0016	0.9951 ± 0.0014	0.9982 ± 0.0005	0.9991 ± 0.0003	0.9848 ± 0.0021	0.9956 ± 0.0015
ER pos vs ER neg	0.949 ± 0.002	0.954 ± 0.002	0.953 ± 0.002	0.940 ± 0.002	0.908 ± 0.003	0.894 ± 0.003	0.914 ± 0.003	0.925 ± 0.003	0.835 ± 0.004	0.875 ± 0.003
KIRC vs KIRP	0.9967 ± 0.0004	0.9983 ± 0.0002	0.9974 ± 0.0004	0.9851 ± 0.0011	0.9682 ± 0.0022	0.9787 ± 0.0015	0.9819 ± 0.0013	0.9837 ± 0.0012	0.9558 ± 0.0024	0.9554 ± 0.0026
LUAD vs LUSC	1.0000 ± 0.0000	1.0000 ± 0.0000	1.0000 ± 0.0000	0.9999 ± 0.0000	0.9999 ± 0.0000	0.9883 ± 0.0006	0.9902 ± 0.0006	0.9972 ± 0.0003	0.9371 ± 0.0023	0.9965 ± 0.0004
Lum A vs Lum B	0.856 ± 0.005	0.860 ± 0.005	0.855 ± 0.005	0.816 ± 0.006	0.775 ± 0.007	0.829 ± 0.006	0.838 ± 0.005	0.836 ± 0.006	0.820 ± 0.007	0.783 ± 0.007
6 cancer	0.9994 ± 0.0000	0.9989 ± 0.0001	0.9993 ± 0.0000	0.9994 ± 0.0000	0.9979 ± 0.0000	0.9901 ± 0.0005	0.9920 ± 0.0002	0.9892 ± 0.0006	0.9883 ± 0.0002	0.9875 ± 0.0006

Table S3: AUC for five binary classification datasets and the one six-class cancer dataset used in this study. Data reported as mean ± SEM.

Dataset	LASSO	Ridge	SVM	RF	NB	DW	SA	Rand	Field	RBM
BRCA vs Normal	0.981 ± 0.002	0.982 ± 0.003	0.980 ± 0.002	0.989 ± 0.002	0.885 ± 0.007	0.973 ± 0.003	0.979 ± 0.003	0.981 ± 0.002	0.947 ± 0.005	0.980 ± 0.002
ER pos vs ER neg	0.950 ± 0.001	0.948 ± 0.001	0.954 ± 0.001	0.949 ± 0.001	0.922 ± 0.002	0.830 ± 0.002	0.843 ± 0.003	0.857 ± 0.002	0.786 ± 0.003	0.892 ± 0.009
KIRC vs KIRP	0.979 ± 0.001	0.977 ± 0.001	0.980 ± 0.001	0.948 ± 0.002	0.942 ± 0.002	0.948 ± 0.002	0.950 ± 0.002	0.957 ± 0.002	0.899 ± 0.003	0.935 ± 0.002
LUAD vs LUSC	0.9988 ± 0.0002	0.9999 ± 0.0001	1.0000 ± 0.0000	0.9958 ± 0.0004	0.9954 ± 0.0004	0.9498 ± 0.0017	0.9554 ± 0.0017	0.9760 ± 0.0013	0.8648 ± 0.0032	0.9970 ± 0.0003
Lum A vs Lum B	0.834 ± 0.004	0.836 ± 0.004	0.835 ± 0.005	0.807 ± 0.004	0.776 ± 0.004	0.783 ± 0.006	0.786 ± 0.005	0.783 ± 0.006	0.777 ± 0.006	0.767 ± 0.007
6 cancer	0.9841 ± 0.0003	0.9780 ± 0.0004	0.9837 ± 0.0004	0.9798 ± 0.0004	0.9610 ± 0.0006	0.7998 ± 0.0041	0.8146 ± 0.0032	0.7882 ± 0.0047	0.7287 ± 0.0013	0.9249 ± 0.0021

Table S4: F1 score for five binary classification datasets and the one six-class cancer dataset used in this study. Data reported as mean ± SEM.

			**PMC**
**Gene Name**	**Functionally Annotated**	**Cancer Hits**	**Breast Cancer Hits**
HGF	1	15967	6356
E2F1	1	13733	5925
GATC	1	1808	212
TIMELESS	1	969	263
HSP90AB1	1	864	299
ESRP1	1	520	322
FAT4	1	437	153
RACGAP1	1	412	221
CDC14B	1	376	118
PGAM5	1	348	89
FKBP4	1	305	127

RFC5	1	303	111
ESPL1	1	260	112
UTRN	1	257	76
ZFP36L2	1	257	65
H2AFZ	1	225	84
VPS25	1	214	36
TUBG1	1	189	81
PTGES3	1	187	50
SRSF9	1	161	59
G6PC3	1	154	37
H6PD	1	149	59
NFS1	1	148	40
RBMS3	1	136	55
RGL1	1	134	47
ABCA6	1	105	47
DENR	1	87	30
VPS33A	1	84	10
PSMC3IP	1	66	28
COQ5	1	61	13
DYNLRB1	1	57	19
PIGU	1	56	24
RCBTB2	1	54	19
TIMM17B	1	51	12
SLC25A39	1	42	11
FTSJ2	1	34	14
MAGI2-AS3	0	25	17
ORMDL2	1	25	10
ENOPH1	1	23	11
NECAB3	1	23	7
TMEM106C	1	23	6
PRR15L	0	21	10
IFFO1	1	17	5
C12orf73	0	0	0

Table S7: Output of semantic search of PubMed Central to assess biological relevance of top 44 genes from PC1 for Luminal A versus Luminal comparison.

Supplemental Experimental Procedures

In this section we provide more details for the methods used in the main text.

Data Sources and Preprocessing

We first describe the sources of the data, then how each data type was preprocessed and scaled, before presenting our dimensionality reduction approaches.

The Cancer Genome Atlas (TCGA) Data

Whole Exome Sequencing, RNA-Seq, miRNA-Seq, DNA Methylation Array, and Genotyping Array data were retrieved from the Genome Data Commons (GDC) data portal (https://portal.gdc.cancer.gov/ - Data Release 4.0) or cBioportal (http://www.cbioportal.org/)[1].

Cancer types with samples having all five data types (messenger-RNA, micro-RNA, copy number variation, single nucleotide polymorphism, and DNA methylation) were chosen for further analysis (Figure 5 and Table S1). The cancer types for the five binomial comparisons were kidney renal clear cell carcinoma (KIRC) vs. kidney renal papillary cell carcinoma (KIRP); lung adenocarcinoma (LUAD) vs. lung squamous cell carcinoma (LUSC); breast invasive carcinoma (BRCA) vs. matched normal breast tissue (normal); estrogen receptor positive (ERpos) vs. estrogen receptor negative (ERneg) breast cancers; and luminal A (LumA) vs. luminal B (LumB) breast cancers. We used human brain, breast, kidney, lung, liver, and colorectal cancer types for the six-cancer multiclass classification. The cancer types which were merged into a single cancer type due to their similarity are colon adenocarcinoma (COAD) and rectum adenocarcinoma (READ); kidney renal clear cell carcinoma (KIRC) and kidney renal papillary cell carcinoma (KIRP); lung adenocarcinoma (LUAD) and lung squamous cell carcinoma (LUSC).

Whole Exome Sequencing (STV)

We retrieved GDC harmonized level 2 Variant Call Format (VCF) files annotated by VarScan2[2] and MuTect[3] GDC somatic annotation workflows (with the Variant Effect Predictor (VEP) v84[4]. VCF files were converted to Genomically Ordered Relational (GOR) database file format[5]. DeepCODE scores (described below) were calculated for all variants. Variants were initially filtered by VCF ‘Filter’ equal to ‘Pass’, VarScan2 p-value less than or equal to 0.05, and ‘Somatic’ status and subsequently filtered by VEP annotation ‘impact’ and deepCODE score and kept if the following conditions were met: (1) 'HIGH' VEP impact, (2) a deepCODE score greater than 0.51 and a 'MODERATE' VEP impact, or (3) 'MODERATE' VEP impact in the absence of a deepCODE score. Call copies for each variant was mapped to its given gene and the counts of all variants ascribed to a given gene were added together into a single count value (referred to as a somatic tumor variant, STV, herein). Variants for the matched breast cancer tumor and normal samples were detected from aligned reads of GDC harmonized level 1 BAM files using the Genome Analysis Toolkit (GATK) Haplotypecaller[6-8]. Joint genotyping was performed on gVCF files using GATK GenotypeGVCFs and hg38 as reference. VEP v85 annotations were obtained by mapping to chromosome position. Variant filtering and call-copy collapsing methods were carried out in the same manner as described above.

RNA-Seq (mRNA)

We retrieved GDC harmonized level 3 mRNA quantification data as un-normalized raw read counts from HT-Seq[9]. Raw mapping counts were combined into a count matrix with genes as rows and samples as columns and normalized using the trimmed mean of M-values (TMM)[10] method from the edgeR[11] R package. Lowly expressed genes were filtered out by requiring read counts to be greater than 1 per million reads for more than 10% of samples. We assessed possible batch effects in the normalized count data using batch information extracted from TCGA barcodes (i.e. the sample plate number) with the ComBat[12] function from the sva[13] R package. There were no detectible batch effects as assessed by Multi-Dimensional Scaling (MDS) either before or after batch correction.

miRNA-Seq (miRNA)

We retrieved GDC harmonized level 3 miRNA quantification data as raw read counts from the BCGSC miRNA profiling pipeline. We filtered miRNAs by retaining only experimentally validated gene targets from the miRBase reference (http://www.mirbase.org/). Raw mapping counts were combined into a count matrix with genes as rows and samples as columns and normalized using the trimmed mean of M-values TMM)[10] method from the edgeR[11] R package. Lowly expressed genes were filtered out by requiring read counts to be greater than 1 per million reads for more than 1% of samples.

Genotyping Arrays (CNV)

We retrieved GISTIC2 processed copy number variation (CNV) data from cBioportal[1,14,15]. GISTIC2 assigns an integer value for each gene ranging from -2 to +2, representing a deep loss, shallow loss, diploid, low-level gain, and high-level amplification accordingly. CNV data was compiled into a matrix with samples as rows and genes as columns and all NA values were removed. For the matched breast cancer tumor and normal samples, we retrieved GDC harmonized level-3 copy number data from Affymetrix SNP 6.0 arrays. The segment means were converted to linear copy numbers using Eq. 1 and mapped to gene symbols using ENSEMBL GRCh38 as reference[16].

$$LinearCopyNumber = 2 \times (2^{SegmentMean}) \qquad (1)$$

CNV segments with less than 5 probes and probe sets with frequent germline copy-number variation (using SNP6 array probe set file as reference) were discarded.

DNA Methylation Arrays (Methylation)

We retrieved GDC harmonized level 3 beta values derived from Illumina Infinium Human Methylation27 (HM27) and HumanMethylation450 (HM450) arrays. Probes were filtered based on the following criteria: (1) was present on both platforms, (2) was mapped to genes or their promoters, (3) was not present on chromosome X, Y, or MT, and (4) did not contain all NA values. We replaced remaining NA and zero beta values with the minimum beta value across all probes and all samples in each batch (defined by the samples TCGA plate barcode) as described in the REMP R package[17]. Beta values of 1 were replaced with the maximum beta value less than 1 across all probes and all samples in each batch. We converted beta values into M values using Eq. 2.

$$M(\beta) = \log_2\left(\frac{\beta}{1-\beta}\right) \qquad (2)$$

We corrected for batch effects within each cancer type using batch information extracted from TCGA barcodes (i.e. the sample plate number) with the ComBat[12] function from the sva[13] R package. We collapsed multiple probes mapped to the same gene by selecting the probe with the maximum standard deviation across all samples.

Genomic Data Integration

We concatenated the processed data from each of five genomic data types (mRNA, miRNA, STV, CNV, and Methylation) into a single data matrix, with samples represented in rows and genes (tagged by data type) as columns. For each comparison, samples were randomly split into 100 cuts of training (80%) and testing (20%) datasets stratified by cancer type and/or molecular subtype.

Normalization

For every cut of training dataset, each feature was scaled to zero mean and unit variance (z-score) and the mean and variance from the training datasets were used to standardize the test datasets.

Dimensionality Reduction

Principal Component Analysis (PCA)

Dimensionality reduction was performed using principal component analysis on each cut of the training data retaining the top 44 principal components as features for the binomial comparisons, and 13 principal components as features for the six-cancer multiclass classification. Each cut of the PC-level data was normalized as mentioned above. In order to avoid data leakage, PCA was performed on the training data, and the test data was then projected onto the PCs defined by the training data. These 100 data matrices with 80% training and 20% testing at the PC level were used for downstream modeling (see Figure 1 for an overview of the classification strategy, and Figure 2 for performance on the binomial comparisons and Figure S1 for performance on the six-cancer multiclass comparison).

EdgeR Analysis

To confirm gene-level classification performance, a simple dual dimensionality reduction and differential analysis approach was performed on a cut of TMM[10] normalized training data of the LumA vs. LumB comparison with edgeR[11], a robust negative binomial model, to determine differentially expressed mRNAs. To account for false discovery, the Benjamini-Hochberg procedure was used to adjust ordinal p-values. The top 44 differentially expressed mRNAs were then used for gene-level classification on the same 100 cuts of the data, though of course with mRNA features, instead of PCA features (see Figure S4).

Decreasing the Amount of Training Data

Based on previous results that showed a benefit for annealing approaches over classical machine learning approaches with smaller amounts of data[18-20], we incrementally decreased the amount of training data for the luminal A (LumA) vs. luminal B (LumB), and ER positive (ERpos) vs. ER negative (ERneg) binomial comparisons, as well as the six-cancer multiclass dataset. To do this, we selected one of the original training cuts that consisted of 80% of the entire dataset. From this one cut, we selected fractions of the data in increments of 5%, making sure that we had at least as many samples as PCs. For example, since the luminal A (LumA) vs. luminal B (LumB) breast cancers dataset had 250 samples and 44 PCs, we selected fractions of data in increments of 5% starting with 20% of the data (20% of 250 samples is 50 samples, which is greater than the number of PCs) up to 95% of the original training cut. In order to collect statistics, for percentage p of the training data we sampled p% of the original training data with all the gene-level features 50 times. For each of these cuts, we reperformed PCA to identify the top 44 PCs of the reduced sub-training set. We trained all classical and Ising models on the same sub-training sets and evaluated performance on the original test set consisting of 20% of the data. The results are presented in Figure 3. Formally, let the original training data set on the gene-level data before PCA be denoted by D_1^{train} which is 80% of the entire dataset (in the main text we repeated this step 100 times, i.e., we had a set of training instances $\{D_i^{train}\}_{i=1}^{100}$), and let the corresponding test data set be noted by D_1^{test}. Here, we selected one of the training cuts and generated 50 "sub"-training data sets for each p, which we denote by $\widetilde{D}_{p,j}^{train}$, where (for LumA vs. LumB) $p \in \{20,25,\ldots,95\}$ and $j \in \{1,\ldots,50\}$. Each $\widetilde{D}_{p,j}^{train}$ is obtained by performing PCA on a randomly selected p% of the 250 samples in D_1^{train} with PCA performed on the original 79,000+ gene-level features. For ERpos vs. ERneg, we set the smallest $p = 10\%$ (Figure S3a), and for the six-cancer class the smallest $p = 5\%$ (Figure S3b).

We also performed the same analysis on the top 44 genes based on their loading for PC1 for the LumA vs. LumB and ERpos vs. ERneg comparisons. Since we are no longer restricted to have as many as features as we were with PCA, we decreased the amount of training data to $p = 5\%$ for LumA vs. LumB and $p = 2\%$ for ERpos vs. ERneg.

Finally, to assess the degree of overfitting, we plotted the performance on the train data and the test data across all training fractions for all 9 classifiers (Figure S2 for the PCA-level features and Figure S8 for the gene-level features), with the difference between train and test being a measure of overfitting. We decided to plot both train and test, rather than just the difference, so that the absolute level of performance between algorithms would be readily apparent; for some fractions of training data, the difference between train and test on a conventional machine learning algorithm was very small, but final training balanced accuracy was around 50% (e.g., Ridge at 20% of the training data in Figure S2a).

Machine Learning

We used five machine learning approaches as conventional classification models. The relevant hyper-parameters for each method are mentioned in their respective sections. Hyper-parameters were chosen by using 10-fold cross-validation on the training data, with performance evaluated on the held-out test data.

Least Absolute Shrinkage and Selection Operator (LASSO), and Ridge Regression

LASSO[21] is an L_1-penalized linear regression model defined as:

$$\hat{\beta}\,(\lambda) = \min_{\beta}\left[-\log\left[L(y;\beta)\right] + \lambda||\beta||_1\right] \quad (3)$$

Ridge[22,23] is an L_2-penalized linear regression model defined as:

$$\hat{\beta}(\lambda) = \min_{\beta} \left[-\log \left[L(y;\beta) \right] + \lambda ||\beta||_2^2 \right] \tag{4}$$

where

$$L = \frac{1}{N} \sum_{i=1}^{N} \left(y_i - \beta_0 - \mathbf{x}_i \cdot \beta \right)^2$$

In both cases $\lambda > 0$ is the regularization parameter that controls model complexity, β are the regression coefficients, β_0 is the intercept term, y are the class labels, x_i is the ith training sample, and the goal of the training procedure is to determine $\hat{\beta}$, the optimal regression coefficients that minimize the quantities defined in Eqs. (3) and (4). The predicted label is given by $\hat{y} = \beta_0 + x_i \cdot \beta$, with some threshold introduced to binarize the label for classification problems. In LASSO, the constraint placed on the norm of β (the strength of which is given by λ) causes coefficients of uninformative features to shrink to zero. This leads to a simpler model that contains only a few non-zero β coefficients. We used the 'glmnet' function from the caret[24] R package to train all LASSO and Ridge models. For Ridge, λ plays a similar role in determining model complexity, except that coefficients for uninformative features do not necessarily shrink to zero.

For both LASSO and Ridge, we chose to implement the function over a custom tuning grid of 1000 values ranging from $\lambda = 0$ to $\lambda = 100$. λ was chosen via 10-fold cross-validation as the value that gave the minimum mean cross-validated error.

Support Vector Machines (SVMs)

Support vector machines (SVMs)[25,26] are a set of supervised learning models used for classification and regression analysis. The primal form of the optimization problem is:

$$\min_{\mathbf{w},b,a} L_p = \frac{1}{2} ||\mathbf{w}||_2^2 = \sum_{i=1}^{N} a_i y_i (\mathbf{x}_i \cdot \mathbf{w} + b) + \sum_{i=1}^{N} a_i \tag{5}$$

where L_p is the loss function in its primal form (p for primal), w are the weights to be determined in the optimization, $\boldsymbol{x_i}$ is the ith training sample, y_i is the label of the ith training sample, $a_i \geq 0$ are Lagrange multipliers, N is the number of training points, and b is the intercept term. Labels are predicted by thresholding $x_i \cdot w + b$.

The optimization problem in its dual form is defined as:

$$\max_{a} L_D(a) = \sum_{i=1}^{N} a_i - \frac{1}{2} \sum_{i,j=1}^{N} a_i a_j y_i y_j K(\mathbf{x}_i, \mathbf{x}_j) \tag{6}$$

where L_D is the Lagrangian dual of the primal problem, a_i are the Lagrange multipliers, y_i and x_i are the ith label and training sample, respectively, $K(\cdot,\cdot)$ is the kernel function. Maximization takes place subject to the constraints $\sum_i a_i y_i = 0$ and $a_i \geq C \geq 0, \forall i$. Here C is a hyper-parameter that controls the degree of misclassification of the model for nonlinear classifiers. The optimal value of w and b can found in terms of the a_i's, and the label of a new data point x can be found by thresholding the output $\sum_i a_i y_i K(x_i, x) + b$.

In most cases, many of the a_i's are zero and evaluating predictions can be faster using the dual form. We used the support vector machines with linear kernel ('svmLinear2') (i.e., $K(x_i, x_j) = x_i \cdot x_j$ the inner product of x_i and x_j) function from the caret[24] R package to train all SVM models. A 10-fold cross-validation was used to tune parameters resulting in best cross-validation accuracy for training the model, using the default tuning grid in *caret*.

Random Forest

Random Forest[27,28] is an ensemble learning method for classification and regression which builds a set (or forest) of decision trees. In random forest, n samples are chosen (typically two-thirds of all the training data) with replacement from the training data m times, giving m different decision trees. Each tree is

grown by considering ‘mtry’ of the total features, and the tree is split depending on which features gives the smallest Gini impurity. In the event of multiple training samples in a terminal node of a particular tree, the predicted label is given by the mode of all the training samples in a terminal node. The final prediction for a new sample x is determined by taking the majority vote over all the trees in the forest. We used the ‘rf’ function from the caret[24] R package to train all Random Forest models. A 10-fold cross-validation was used to tune parameters for training the model. A tune grid with 44 values from 1 to 44 for ‘mtry’, the number of random variables considered for a split each iteration during the construction of each tree, was used for the tuning model.

Naïve Bayes

Naïve Bayes[29,30] is a conditional probabilistic classifier based on applying Bayes' theorem which relies on strong independence assumptions, as defined by Eqs. 7 and 8:

$$P(y_i|\mathbf{x}_i) = \frac{P(y_i)P(\mathbf{x}_i|y_i)}{P(\mathbf{x}_i)} \tag{7}$$

$$P(y_i|x_{i,1},\ldots,x_{i,m}) = P(y_i)\prod_{k=1}^{m} P(x_{i,k}|y_i) \tag{8}$$

where $x_{i,k}$ is the kth feature of the ith training sample x_i, y_i is the given class label, and m is the number of features. We used the ‘nb’ function from the caret[24] R package to train all Naïve Bayes models.

Computational Frameworks and Resources

Data pre-processing and machine learning models were carried out using R (>= 3.4.4) or Python (3.6.8). Plots were generated using ggplot2 in R.

Methods for Gene-Level Analysis of LumA vs. LumB

Differential Gene Expression Analysis

To generate Figure 4, we performed differential expression analysis for 41 mRNA genes from top 44 most informative PC1 genes in LumA vs. LumB breast cancer comparison. The edgeR[11] package was used to determine differentially expressed mRNAs. The Benjamini-Hochberg was used to control for false discovery of 5%. Of the 41 mRNA genes, we found 40 genes were significantly differentially expressed with an FDR ≤ 0.05. We found 30 genes had higher expression in Luminal B and 11 genes had higher expression in Luminal A samples based on edgeR analysis. Moreover, there were a total 7,871/18,059 (44%) differently expressed mRNA genes for the Luminal A vs. Luminal B breast cancer comparison. Of these 7,871 genes, 4,345 (55%) were up regulated in Luminal B compared to 3,526 (45%) in Luminal A. To confirm similar performance on PCA derived gene-level classification results, a second edgeR analysis, independent of PCA dimensionality reduction, was also performed on the LumA vs. LumB comparison as described above.

Functional Enrichment Analysis (GOseq)

Functional enrichment analysis of the top 44 most informative genes by PC loading of PC1 from the training set of luminal A (LumA) vs. luminal B (LumB) breast cancers comparison was carried out with GOseq[31] analysis in an unrestricted manner. Briefly, GOseq analysis was performed on the top 44 gene list to identify enriched gene ontology (GO) terms allowing unannotated genes in the analysis. Select GOseq terms ordered by p-value are shown in Figure 4d. A complete list of functionally enriched GO terms is presented in Table S6.

Semantic Search Engine

The ‘*entrez search*’ function from the R package ‘*rentrez*’[32] was used to query the number of full-text publications for each of the top 44 most informative genes in Luminal A vs. Luminal B breast cancer comparison from the PubMed Central (PMC) database. Briefly, the R package ‘*rentrez*’ provides an

interface to the NCBI's '*EUtils*' API to search databases like GenBank [https://www.ncbi.nlm.nih.gov/genbank/] and PubMed [https://www.ncbi.nlm.nih.gov/pubmed/] for relationships between genes of interest and query terms, and to process the results from the retrieved hits. The search term was defined by combining the gene symbol and "*cancer*" or "*breast cancer*" fields, along with the Medical Subject Headings (MeSH) vocabulary terms as synonyms to expand each NLP search using Boolean operators *AND/OR* (see Table S7). Network diagrams were constructed using Circos scripts (http://circos.ca/). The red and blue outer bands represent 'mRNA' and 'methylation' datatypes, respectively. The inner blue band are genes with known functional annotation at the time of analysis. The purple colored ring indicates the total number of publications where each gene and cancer are both mentioned. This band is colored with five bins where white is the lowest and dark purple the highest. For example, there are many publications that mentions both "E2F1" and "cancer", and very few with "C12orf73" and "cancer". The thickness and color of the Circos plot ribbons indicate number of published full-text articles linking each gene to the cancer or breast cancer.

Hierarchical Clustering

We applied a "custom ward" linkage criteria in the hierarchical cluster[33] analysis of top 44 most informative genes, by PC loading, of PC1 from the training set of the luminal A (LumA) vs. luminal B (LumB) breast cancers comparison (Figure 4b). The genes are represented as rows, and samples as columns. The algorithm used an exact minimization procedure.

Quantum Annealing

Quantum annealing may be considered a special case of adiabatic quantum computation[34]. The adiabatic theorem of quantum mechanics, which underlies quantum annealing, implies that a physical system will remain in the ground state if a given perturbation acts slowly enough and if there is a gap between the ground state and the rest of the system's energy spectrum[35]. To use the adiabatic theorem to solve optimization problems, we specify an initial Hamiltonian, H_B, whose ground state is easy to find (typically a transverse field), and a problem Hamiltonian, H_P, that does not commute with H_B and whose ground state encodes the solution to the problem we are seeking to optimize[36]. We then interpolate from H_B to H_P by defining the total Hamiltonian $H(s) = A(s)H_B + B(s)H_P$, where s is the parameterized time ($0 \leq s = t/t_f \leq 1$, t is time, and t_f is the total annealing time), $A(s)$ and $B(s)$ are, respectively, decreasing and increasing smoothly and monotonically. The adiabatic theorem ensures that the ground state of the system at $s = 1$ will give the desired solution to the problem, provided the interpolation is sufficiently slow, i.e., t_f is large compared to the timescale set by the inverse of the smallest ground state gap of $H(s)$ and by $\frac{dH(s)}{ds}$ [37]. In quantum annealing, rather than run the computation a single time slowly enough such that the adiabatic theorem is obeyed, we allow the possibility of running the computation multiple times at a shorter annealing time, such that the overall computational time is minimized[38]. In addition, when quantum annealing is implemented in a physical device, temperature and other noise effects play an important role; thermal excitation and relaxation cannot be neglected and affect performance[39-41].

Additional technical details regarding the D-Wave quantum annealers

D-Wave processors currently employ a "Chimera" architecture with a limited graph connectivity (for a typical representation of a hardware graph, see Supplemental Figure S8). For nearly all problems of practical interest, the connectivity of the "logical problem" will differ from the Chimera architecture of D-Wave. This introduces the need to find a *minor embedding* of the hardware graph[42,43]. A minor embedding maps a logical problem qubit to a set of physical qubits such that for every coupling between pairs of logical qubits in the logical problem there exists at least one physical coupling between the corresponding sets of physical qubits. A minor embedding is found by performing a series of edge contractions, which effectively join vertices together, thereby allowing for a graph with fewer vertices but a higher degree of connectivity to be obtained[44]. For the results in this study, we used the "minorminer" package available on D-Wave's github [https://github.com/dwavesystems/minorminer].

In order to ensure that physical qubits are aligned and act as a single logical qubit (or "chain"), a strong coupling bias is introduced between physical qubits that comprise a logical qubit. Then, for a fixed embedding, the way the values of the couplings and local fields for a logical qubit are distributed among the physical qubits is known as "parameter setting". A built-in function provided by D-Wave[43] has been

used for parameter setting. By the embedding procedure and parameter setting, logical problems may be transformed into physical problems. Note that for one logical problem there may be many physical problems, depending both on the embedding and the parameter setting.

Ideally, once the strength of the coupling between logical qubits is determined, all solutions returned by D-Wave would correspond to valid logical solutions, i.e., all the physical qubits within a logical qubit would have the same spin (there would be no "broken chains"). However, due to the probabilistic nature of quantum annealing, as well as noise from different sources, there is often some percentage of solutions that have broken chains. To deal with broken chains D-Wave offers three options for "decoding" the solutions. The first is to discard all solutions with broken chains and collect an additional set of solutions ("discard"). Another option is to do a majority vote on the physical qubits that comprise a logical qubit, breaking ties with a random assignment ("majority vote decoding"). The last option is to go through the broken chains one by one and select the value for the spin that greedily minimizes the energy of the Hamiltonian of the logical problem ("energy minimization decoding"); i.e., it selects the spin that result in the greatest decrease in the energy of the Hamiltonian based on the current spin configuration. The likelihood of a solution having broken chains can be roughly adjusted by controlling a parameter J_c, the value of the strong coupling bias between physical qubits within a logical qubit; the larger the magnitude of J_c, the more likely will it be for the physical qubits within a logical qubit to have the same spin. The disadvantage of increasing the coupling bias too much is that it can wash out the details of the problem instances; thus, there is a tradeoff between getting solutions with many broken chains (which occurs when $|J_c|$ is too small) and getting solutions which may have lost the details of the original problem we are trying to solve (which occurs when $|J_c|$ is too large).

Based on these considerations, our strategy for collecting solutions was the following. First, we generated 20 embeddings based on the procedure mentioned above. The embedding with the smallest average number of physical qubits per logical qubit was used to obtain weights for all the training instances. For 2000Q, this embedding had 26.8 physical qubits per logical qubit on average, and 1747 qubits were used in total. The final embedding used for DW2X runs had 19.7 physical qubits per logical qubits on average, with a total of 887 qubits used. Then, for each training instance we queried the D-Wave chip for 1000 times with 10 spin-reversal transformations (or, gauges[45]) to mitigate parameter misspecifications from the machine. We then treated J_c as a hyper-parameter with values in the set $\{-0.5, -1, -3, -8, -16, -32\}$. All the parameters sent to the machine (both the ferromagnetic coupling J_c and the physical problem parameters h_i and J_{ij}) are normalized to fall between −1 and 1, per specifications of the machine. Finally, classical post-processing optionally may be performed on the broken chains. Energy-minimization and majority-vote decoding are quick and speed up collection of solutions; however, in principle, one could treat the post-processing approach as a hyper-parameter, but to avoid introducing too much classical post-processing and avoid spending too much time generating more solutions, we selected majority voting to post-process the solutions. All D-Wave anneals were run with an annealing time of 5μs. Note that we did not optimize the annealing time; doing so would introduce another hyperparameter and could improve results for D-Wave.

Derivation of the Ising Hamiltonian

Recall that we have written the probabilities for the first $K-1$ classes as:

$$Pr(y_i = k) = \frac{\exp \mathbf{w}_k^\intercal \mathbf{x}_i}{1 + \sum_{k=1}^{K-1} \exp \mathbf{w}_k^\intercal \mathbf{x}_i}, \tag{9}$$

with the probability of the *K*th class as:

$$Pr(y_i = K) = \frac{1}{1 + \sum_{k=1}^{K-1} \exp \mathbf{w}_k^\intercal \mathbf{x}_i}. \tag{10}$$

By defining the probabilities of our classes in this way, we can reduce the number of sets of weights we have to train from K to $K-1$. The goal of training is to maximize the probability given the classes in the dataset, or equivalently to minimize the negative log-likelihood. we can express the negative log-likelihood as follows:

$$\mathcal{L} = -\log \prod_i Pr(y_i) \tag{11}$$

$$= -\sum_i \log Pr(y_i) \tag{12}$$

For simplicity, we define $z_i^{(k)} = w_k^\intercal x_i$, i.e., the inner product between the weights corresponding to the kth and the ith feature-vector. To continue, we consider splitting the above sum into terms over the first $K-1$ classes and the Kth class:

$$\mathcal{L} = -\sum_i \log Pr(y_i)$$

$$= -\sum_{i:y_i\in 1\ldots K-1} \log Pr(y_i) - \sum_{i:y_i=K} \log Pr(y_i = K) \tag{13}$$

$$= -\sum_{i:y_i\in 1\ldots K-1} \left[z_i^{(y_i)} - \log\left(1 + \sum_{k=1}^{K-1} \exp z_i^{(k)}\right)\right] + \sum_{i:y_i=K} \log\left(1 + \sum_{k=1}^{K-1} \exp z_i^{(k)}\right) \tag{14}$$

$$= -\sum_{i:y_i\in 1\ldots K-1} z_i^{(y_i)} + \sum_i \log\left(1 + \sum_{k=1}^{K-1} \exp z_i^{(k)}\right), \tag{15}$$

We can now take the second-order Taylor approximation around 0 for the second summation, expanding in $z_i^{(k)}$ gives us the following:

$$\mathcal{L} \approx -\sum_{i:y_i\in 1\ldots K-1} z_i^{(y_i)} + \sum_i \left[\log K + \frac{1}{K}\sum_k z_i^{(k)} + \frac{1}{2}\frac{K-1}{K^2}\sum_k \left(z_i^{(k)}\right)^2 - \frac{1}{2K^2}\sum_{k,j\neq k} z_i^{(j)} z_i^{(k)}\right] \tag{16}$$

$$= -\sum_{i:y_i\in 1\ldots K-1} \mathbf{w}_{y_i}^\intercal \mathbf{x}_i + \sum_{i,k}\left[\frac{1}{K}\mathbf{w}_k^\intercal \mathbf{x}_i + \frac{K-1}{2K^2}\mathbf{w}_k^\intercal \mathbf{x}_i \mathbf{x}_i^\intercal \mathbf{w}_k\right] - \frac{1}{2K^2}\sum_{i,k,j\neq k} \mathbf{w}_j^\intercal \mathbf{x}_i \mathbf{x}_i^\intercal \mathbf{w}_k \tag{17}$$

$$= \sum_{k=1}^{K-1} \mathbf{w}_k^\intercal \left(\sum_{i:y_i=k} -\mathbf{x}_i + \frac{1}{K}\sum_i \mathbf{x}_i\right) + \sum_{k=1}^{K-1} \mathbf{w}_k^\intercal \left(\frac{K-1}{2K^2}\sum_i \mathbf{x}_i\mathbf{x}_i^\intercal\right)\mathbf{w}_k - \sum_{k,j\neq k} \mathbf{w}_j^\intercal \left(\frac{1}{2K^2}\sum_i \mathbf{x}_i\mathbf{x}_i^\intercal\right)\mathbf{w}_k \tag{18}$$

$$= \sum_{k=1}^{K-1} \mathbf{w}_k^\intercal(\mathbf{b}_k + \mathbf{h}') + \sum_{k=1}^{K-1} \mathbf{w}_k^\intercal \mathbf{J}' \mathbf{w}_k - \sum_{k=1}^{K-1}\sum_{j\neq k} \mathbf{w}_j^\intercal \mathbf{J}'' \mathbf{w}_k \tag{19}$$

where

$$\mathbf{b}_k = \sum_{i:y_i=k} -\mathbf{x}_i, \tag{20}$$

$$\mathbf{h} = \frac{1}{K}\sum_i \mathbf{x}_i, \tag{21}$$

$$\mathbf{J}' = \frac{K-1}{2K^2}\sum_i \mathbf{x}_i\mathbf{x}_i^\intercal, \tag{22}$$

$$\mathbf{J}'' = \frac{1}{2K^2}\sum_i \mathbf{x}_i\mathbf{x}_i^\intercal. \tag{23}$$

In examining the derivation, one may ask whether it is reasonable to take an expansion to second-order near $z_i^{(k)} = 0$. We offer two brief arguments in support of this. The first is that a second-order approximation has been used to great success in other algorithms, such as XGBoost, a gradient-boosted algorithm that has seen much success recently in a variety of machine learning tasks. To speed up calculations, XGBoost uses a second-order approximation to calculate the objective function in a general setting. It is important to note, however, that for XGBoost (and other gradient-based methods) the weights are updated iteratively, whereas here we are presumably using a quantum annealer to directly evaluate the loss function. A second argument is that we are looking for a set of self-consistent solutions. We take the second-order approximation around 0, and if the optimization works properly, we will get results for which the approximation is valid.

Perhaps a more serious concern is that this expansion is not formally within the radius of convergence of the natural logarithm. Given this concern, care should be given to make sure that the difference in the approximation does not differ too greatly. One simple way to check this is to see whether there is a clear correlation between the energies (the approximation) and the original function we are trying to optimize (the log-likelihood). As long as there is good correlation, the approximation is reasonable. The correlation between the negative log-likelihood and the energy for the five binomial datasets is shown in Supplemental Figure S6 (more on this in the subsection titled "Performance metrics versus energy", below). For binary classification, the negative log-likelihood is equivalent to the logistic loss, $l = ln(1 + exp(-y_i w \cdot x_i))$, if we use the label convention $y_i \in \{-1,1\}$, or the binary cross-entropy loss, $l_{CE} = -y_i \, ln \, \sigma \, (w \cdot x_i) - (1 - y_i) \, ln\big(1 - \sigma(w \cdot x_i)\big)$ where $\sigma(z) = 1/(1 + exp(-z))$, if we use the convention $y_i \in \{0,1\}$. We sometimes refer to the negative log-likelihood as the logistic loss.

Post-processing Spin Configurations

In this section we describe our classical post-processing procedure to make use of all the spin configurations returned by D-Wave, SA and Random. We used all three methods to generate S different spin configurations (which we refer to as "weights") and sorted them by their Ising energy. Weights were then averaged together and the averaged weights that gave the best performance for some training metric was selected. More formally, let $\{w_i\}_{i=1}^{S}$be the set of S weights returned by the various methods. We define $w_{tmp}^k = \frac{1}{k}\sum_{i=1}^{k} w_i$ as the kth trial weight, and $\bar{f}_k = \frac{1}{|D^{train}|}\sum_{j \in D^{train}} f\big(y_j, \hat{y}_j^k\big)$ where f is the performance metric, D^{train} is the training data set, and $\hat{y}_j^k$ is the predicted output of the kth trial weights w_{tmp}^k on the jth training sample. The metrics of training performance include AUC, the logistic loss, and the accuracy. For the AUC and logistic loss we can directly use the predicted output (for binary classification, the predicted probability of the jth sample to be of class 1 is $\hat{y}_k = \sigma\big(w \cdot x_j\big)$. For the accuracy, we assign labels based on whether the predicted output is greater than 0.5. Applying this averaging procedure for a small set of weights allows us to increase the performance without sacrificing some of the robustness associated with discrete weights. Unless otherwise specified, for all Figures in the Main text and here, we used $S = 20$ and set f = AUC as the performance metric.

Supplemental References

1 Cerami, E. *et al.* (2012). The cBio Cancer Genomics Portal: An Open Platform for Exploring Multidimensional Cancer Genomics Data. *Cancer Discovery* **2**, 401- 404.

2 Koboldt, D. C. *et al.* (2012). VarScan 2: somatic mutation and copy number alteration discovery in cancer by exome sequencing. *Genome Research* **22**, 568-576.

3 Cibulskis, K. *et al.* (2013). Sensitive detection of somatic point mutations in impure and heterogeneous cancer samples. *Nature Biotechnology* **31**, 213-219.

4 McLaren, W., Gil, L., Hunt, S. E., Riat, H. S., Ritchie, G. R., Thormann, A., Flicek, P. & Cunningham, F. (2016). The ensembl variant effect predictor. *Genome Biology* **17**, 122.

5 Guðbjartsson, H. *et al.* (2016). GORpipe: a query tool for working with sequence data based on a Genomic Ordered Relational (GOR) architecture. *Bioinformatics* **32**, 3081-3088.

6 DePristo, M. A. *et al.* (2011). A framework for variation discovery and genotyping using next-generation DNA sequencing data. *Nature Genetics* **43**, 491-498.

7 McKenna, A. *et al.* (2010). The Genome Analysis Toolkit: a MapReduce framework for analyzing next-generation DNA sequencing data. *Genome Research* **20**, 1297-1303.

8 Van der Auwera, G. A. *et al.* (2013). From FastQ data to high-confidence variant calls: the genome analysis toolkit best practices pipeline. *Current Protocols in Bioinformatics* **43**, 11.10. 11-11.10. 33.
9 Anders, S., Pyl, P. T. & Huber, W. (2015). HTSeq—a Python framework to work with high-throughput sequencing data. *Bioinformatics* **31**, 166-169.
10 Robinson, M. D. & Oshlack, A. (2010). A scaling normalization method for differential expression analysis of RNA-seq data. *Genome Biology* **11**, R25.
11 Robinson, M. D., McCarthy, D. J. & Smyth, G. K. (2010). edgeR: a Bioconductor package for differential expression analysis of digital gene expression data. *Bioinformatics* **26**, 139-140.
12 Johnson, W. E., Li, C. & Rabinovic, A. (2007). Adjusting batch effects in microarray expression data using empirical Bayes methods. *Biostatistics* **8**, 118-127.
13 Leek, J., Johnson, W., Parker, H., Jaffe, A. & Storey, J. (2013). SVA: Surrogate Variable Analysis. R Package Version 3.
14 Gao, J. *et al.* (2013). Integrative analysis of complex cancer genomics and clinical profiles using the cBioPortal. *Sci. Signal.* **6**, pl1.
15 Beroukhim, R. *et al.* (2007). Assessing the significance of chromosomal aberrations in cancer: methodology and application to glioma. *Proceedings of the National Academy of Sciences* **104**, 20007-20012.
16 Hubbard, T. *et al.* (2002). The Ensembl genome database project. *Nucleic Acids Research* **30**, 38-41.
17 Zheng, Y., Joyce, B. T., Liu, L., Zhang, Z., Kibbe, W. A., Zhang, W. & Hou, L. (2017). Prediction of genome-wide DNA methylation in repetitive elements. *Nucleic Acids Research* **45**, 8697-8711.
18 Li, R. Y., Di Felice, R., Rohs, R. & Lidar, D. A. (2018). Quantum annealing versus classical machine learning applied to a simplified computational biology problem. *npj Quantum Information* **4**, 14.
19 Mott, A., Job, J., Vlimant, J.-R., Lidar, D. & Spiropulu, M. (2017). Solving a Higgs optimization problem with quantum annealing for machine learning. *Nature* **550**, 375-379.
20 Willsch, D., Willsch, M., De Raedt, H. & Michielsen, K. (2019). Support vector machines on the D-Wave quantum annealer. *arXiv preprint arXiv:1906.06283*.
21 Tibshirani, R. (1996). Regression shrinkage and selection via the lasso. *Journal of the Royal Statistical Society: Series B (Methodological)* **58**, 267-288.
22 Hoerl, A. E. & Kennard, R. W. (1970). Ridge regression: Biased estimation for nonorthogonal problems. *Technometrics* **12**, 55-67.
23 Hoerl, A. E., Kannard, R. W. & Baldwin, K. F. (1975). Ridge regression: some simulations. *Communications in Statistics-Theory and Methods* **4**, 105-123.
24 Kuhn, M. (2008). Building predictive models in R using the caret package. *Journal of Statistical Software* **28**, 1-26.
25 Boser, B. E., Guyon, I. M. & Vapnik, V. N. (ACM, 1992).in *Proceedings of the Fifth Annual Workshop on Computational Learning Theory.* 144-152.
26 Cortes & Vapnik, V. (1995). Support-vector networks. *Machine Learning* **20**, 273-297.
27 Breiman, L. (2001). Random forests. *Machine Learning* **45**, 5-32.
28 Breiman, L., Friedman, J., Stone, C. J. & Olshen, R. A. (Chapman & Hall, 1993). *Classification and regression trees*.
29 Hastie, T., Tibshirani, R. & Friedman, J. H. (Springer, 2016). *The Elements of Statistical Learning: Data Mining, Inference, and Prediction*. 2 edn.
30 Ng, A. Generative Learning algorithms. (2008).
31 Young, M. D., Wakefield, M. J., Smyth, G. K. & Oshlack, A. (2010). Gene ontology analysis for RNA-seq: accounting for selection bias. *Genome Biology* **11**, R14.
32 Winter, D. J. rentrez: An R package for the NCBI eUtils API. Report No. 2167-9843, (PeerJ Preprints, 2017).
33 Ward Jr, J. H. (1963). Hierarchical grouping to optimize an objective function. *Journal of the American Statistical Association* **58**, 236-244.
34 Albash, T. & Lidar, D. A. (2018). Adiabatic quantum computation. *Reviews of Modern Physics* **90**, 015002.
35 Kato, T. (1950). On the adiabatic theorem of quantum mechanics. *Journal of the Physical Society of Japan* **5**, 435-439.

36 Farhi, E., Goldstone, J., Gutmann, S. & Sipser, M. (2000). Quantum computation by adiabatic evolution. *arXiv preprint arXiv:quant-ph/0001106*.
37 Jansen, S., Ruskai, M.-B. & Seiler, R. (2007). Bounds for the adiabatic approximation with applications to quantum computation. *Journal of Mathematical Physics* **48**, 102111.
38 Rønnow, T. F., Wang, Z., Job, J., Boixo, S., Isakov, S. V., Wecker, D., Martinis, J. M., Lidar, D. A. & Troyer, M. (2014). Defining and detecting quantum speedup. *Science* **345**, 420-424.
39 Childs, A. M., Farhi, E. & Preskill, J. (2001). Robustness of adiabatic quantum computation. *Physical Review A* **65**, 012322.
40 Amin, M. H., Averin, D. V. & Nesteroff, J. A. (2009). Decoherence in adiabatic quantum computation. *Physical Review A* **79**, 022107.
41 Albash, T. & Lidar, D. A. (2015). Decoherence in adiabatic quantum computation. *Physical Review A* **91**, 062320.
42 Choi, V. (2008). Minor-embedding in adiabatic quantum computation: I. The parameter setting problem. *Quantum Information Processing* **7**, 193-209.
43 Cai, J., Macready, W. G. & Roy, A. (2014). A practical heuristic for finding graph minors. *arXiv preprint arXiv:1406.2741*.
44 Robertson, N. & Seymour, P. D. (1984). Graph minors. III. Planar tree-width. *Journal of Combinatorial Theory, Series B* **36**, 49-64.
45 Boixo, S., Albash, T., Spedalieri, F. M., Chancellor, N. & Lidar, D. A. (2013). Experimental signature of programmable quantum annealing. *Nature Communications* **4**, 2067.
