# Supplementary material for: Quantum processor-inspired machine learning in the biomedical sciences
Source: Patterns (N Y). 2021 Apr 28;2(6):100246. doi: 10.1016/j.patter.2021.100246 (PMC8212142; doi:10.1016/j.patter.2021.100246)
Supplement: Document S2. Article plus supplemental information [file mmc4.pdf]

Article

Patterns

Quantum processor-inspired machine learning in the biomedical sciences

Highlights

- Ising-type algorithms perform better with small datasets
- There is less overfitting with Ising-type algorithms
- Ising-type algorithms are amenable for drug discovery
- Ising-type algorithms are useful for assessment of drug efficacy

Authors

Richard Y. Li, Sharvari Gujja, Sweta R. Bajaj, ..., Jeffrey R. Gulcher, Daniel A. Lidar, Thomas W. Chittenden

Correspondence

lidar@usc.edu (D.A.L.), tom.chittenden@genuitysci.com (T.W.C.)

In brief

Inspired by quantum process, Ising-type algorithms demonstrate better classification performance than standard machine learning approaches when training with small amounts of data. This suggests a potential application for rare diseases or other clinical data where the amount of training data may be limited.

Li et al., 2021, Patterns 2, 100246
June 11, 2021 © 2021 The Authors.
https://doi.org/10.1016/j.patter.2021.100246

CellPress

Patterns

CellPress
OPEN ACCESS

Article

Quantum processor-inspired machine learning in the biomedical sciences

Richard Y. Li,[1,2,3] Sharvari Gujja,[4,5] Sweta R. Bajaj,[4,5] Omar E. Gamel,[4] Nicholas Cilfone,[4] Jeffrey R. Gulcher,[6] Daniel A. Lidar,[1,3,7,8,*] and Thomas W. Chittenden[4,5,9,10,*]

[1]Department of Chemistry, University of Southern California, 920 Bloom Walk, Los Angeles, CA 90089, USA
[2]Computational Biology and Bioinformatics Program, Department of Biological Sciences, University of Southern California, Los Angeles, CA, USA
[3]Center for Quantum Information Science & Technology, University of Southern California, Los Angeles, Boston, CA, USA
[4]Computational Statistics and Bioinformatics Group, Genuity AI Research Institute, Genuity Science, 90 Canal Street, Suite 120, Boston, MA 02114, USA
[5]Complex Biological Systems Alliance, Medford, MA, USA
[6]Cancer Genetics Group, Genuity Science, Boston, MA, USA
[7]Department of Electrical Engineering, University of Southern California, Los Angeles, CA, USA
[8]Department of Physics and Astronomy, University of Southern California, Los Angeles, CA, USA
[9]Division of Genetics and Genomics, Boston Children's Hospital, Harvard Medical School, Boston, MA, USA
[10]Lead contact
*Correspondence: lidar@usc.edu (D.A.L.), tom.chittenden@genuitysci.com (T.W.C.)
https://doi.org/10.1016/j.patter.2021.100246

THE BIGGER PICTURE Advances in sequencing technology, leading to an ever-increasing volume and variety of data, present an opportunity to probe the molecular underpinnings of disease. Inspired in part by recent developments in physical quantum processors, we evaluated several Ising-type algorithms, which are relatively unused in the biomedical sciences, on actual human tumor data from The Cancer Genome Atlas. Our results show performance competitive with conventional machine-learning algorithms in classifying human cancer types and associated molecular subtypes when training with all available data; but perhaps more strikingly, the Ising-type algorithms demonstrate superior performance with smaller training datasets. This gain in performance suggests a tantalizing application for rare diseases or other clinical applications where the number of training samples may be quite small. In addition, the features extracted from the Ising-type algorithms have biological relevance.

1 2 3 4 5 **Proof-of-Concept:** Data science output has been formulated, implemented, and tested for one domain/problem

SUMMARY

Recent advances in high-throughput genomic technologies coupled with exponential increases in computer processing and memory have allowed us to interrogate the complex molecular underpinnings of human disease from a genome-wide perspective. While the deluge of genomic information is expected to increase, a bottleneck in conventional high-performance computing is rapidly approaching. Inspired by recent advances in physical quantum processors, we evaluated several unconventional machine-learning (ML) strategies on actual human tumor data, namely "Ising-type" methods, whose objective function is formulated identical to simulated annealing and quantum annealing. We show the efficacy of multiple Ising-type ML algorithms for classification of multi-omics human cancer data from The Cancer Genome Atlas, comparing these classifiers to a variety of standard ML methods. Our results indicate that Ising-type ML offers superior classification performance with smaller training datasets, thus providing compelling empirical evidence for the potential future application of unconventional computing approaches in the biomedical sciences.

INTRODUCTION

With the rapid expansion of high-throughput genomic technologies there exists a multitude of "omics" data, which allows researchers to now investigate the causal molecular drivers of complex human disease with a systems biology approach. Over the past 2 decades, numerous studies have shown the utility of statistical machine-learning (ML) strategies to classify

This is an open access article under the CC BY license (http://creativecommons.org/licenses/by/4.0/).

human malignancies, hypothesize unknown clinical subtypes, and make prognostic predictions based on omics datasets.[1,2] Moreover, integrated "multi-omics" approaches have proved effective in deriving meaningful biological insights into the etiological and prognostic complexity of human cancers.[3–6] While these studies highlight the potential of omics-based analytics to drive innovative new therapies based on unique molecular signatures, several well-documented issues, including correlation bias, feature dependency, and multicollinearity, still hamper statistical optimization for the analysis and robust classification of high-dimensional complex biological datasets.[7]

To address some of these statistical computing limitations, we present a class of unconventional "Ising-type" ML algorithms, inspired by quantum computing. As a rapidly emerging technology, quantum computing promises to enhance the performance of certain classes of statistical computing and ML tasks, such as classification, regression, generation, and resampling. In this nascent discipline, proposals for several quantum ML algorithms have been developed, including quantum principal-component analysis (PCA)[8] and quantum support vector machines[9] and Boltzmann machines.[10] These proposals have generated interest in the scientific community and in the general public for their potential to address computationally intractable tasks and to model more complicated data distributions. One of the unconventional ML approaches used in this study, quantum annealing with processors made by D-Wave Systems,[11–13] features more than 2,000 qubits, becoming large enough to solve real-world problems,[14] perform quantum simulation,[15] and compete with classical optimization algorithms.[16] While the computational role of quantum effects in these processors remains controversial and the subject of intensive study, quantum annealing is currently one of the few paradigms of quantum computing that are approaching a scale useful for practical applications.

Using high-dimensional multi-omics human cancer data from The Cancer Genome Atlas (TCGA), we framed a classification problem in such a way that it was amenable to solving with Ising-type approaches. The Ising-type methods must be formulated as a quadratic unconstrained binary optimization (QUBO) or, equivalently, an Ising Hamiltonian $H(w) = w^{\top}h + w^{\top}Jw$, where $\boldsymbol{w}$ is a vector of weights, and h and J represent a vector and a matrix, respectively. We compared Ising-type approaches to standard ML approaches for both binomial and multiclass experimental designs. Although previous studies have applied quantum annealing and other Ising models to model protein folding,[17] transcription factor DNA binding,[18] and classification of lung cancer data with microarray data,[19] our analysis is the first of integrated, genome-wide multi-omics human cancer data. In the course of our study, we found that the Ising models all perform similarly to each other. Our results further show that, in most cases, when using relatively large amounts of high-dimensional multi-omics training data, the Ising-type methods are comparable to standard supervised ML approaches. However, for smaller training datasets of equivalent dimensionality, Ising models statistically outperform established classification strategies. We also assessed the weights returned by the Ising models and found reasonable interpretability and generalizability of biological information. Overall, our results demonstrate the current utility and limitations of Ising models applied to the analysis of high-dimensional omics data and point to a general class of algorithms that may be useful when training data are limited.

RESULTS

We assessed the performance of annealing-based Ising ML algorithms on several TCGA datasets to identify comparative advantages for the Ising approaches. In this ML survey, we compared the performance of Ising models to that of the following commonly used ML algorithms: least absolute shrinkage and selection operator (LASSO),[20] ridge regression (Ridge),[21,22] random forest (RF),[23,24] naive Bayes (NB),[25,26] and a support vector machine (SVM).[27,28] TCGA data, including exome DNA variation, RNA sequencing (RNA-seq), DNA methylation, microRNA (miRNA), and copy number variations (CNVs), were retrieved, preprocessed, and normalized, resulting in an average of 70,504 gene features for five binomial and one multiclass six-cancer TCGA dataset comparison. We performed dimensionality reduction with PCA (see supplemental experimental procedures), retaining the top 44 principal components (PCs) for the binomial datasets and 13 PCs for the six-cancer dataset. The number of PCs was chosen based on the largest number of features that could be accommodated on existing quantum annealing hardware. An overview of our data analysis strategy is presented in Figure 1.

Quantum annealing was implemented on D-Wave physical quantum processors (see supplemental experimental procedures). As mentioned, D-Wave admits problems only when formulated as a QUBO problem or, equivalently, an Ising Hamiltonian, generically written as $H(\boldsymbol{w}) = \boldsymbol{w}^{\top}\boldsymbol{h} + \boldsymbol{w}^{\top}\boldsymbol{J}\boldsymbol{w}$, where $\boldsymbol{w}$ is a vector of weights, $\boldsymbol{J}$ is the matrix of interactions, and $\boldsymbol{h}$ is the local fields. The goal of the learning procedure is to find an optimal set of weights that minimizes the energy of the Ising Hamiltonian, i.e., find $\boldsymbol{w}^* = argmin_{\boldsymbol{w}} H(\boldsymbol{w})$. The global optimum of the Ising problem is in general difficult to determine.[29] Classification by using quantum annealing to solve Ising problems has been formulated before.[30] In the present work, we developed a novel approach that can be used to solve classification problems directly. Our strategy stems from multinomial regression, which reduces to logistic regression when there are two classes (see supplemental experimental procedures for the mapping to an Ising problem). We compared the performance with several other Ising models that use the same objective function as D-Wave, i.e., problems formulated as an Ising Hamiltonian: simulated annealing (SA), Random, and Field. SA[31] is a well-known heuristic optimization algorithm that uses Metropolis updates and a (fictitious) temperature schedule to optimize a target objective function. For Random, we randomly generated candidate weights, sorted them by their Ising energy, and selected the best performing weights. For Field, we disregarded $\boldsymbol{J}$, the coupling terms, and performed an optimization only over $\boldsymbol{h}$, the local fields (see supplemental experimental procedures for more details of all classical, quantum, and quantum-inspired algorithms). Both Random and Field were introduced and used as simple benchmarks against which we tested the SA and quantum annealing approaches.

Last, we compared the performance of the annealing-based models to a restricted Boltzmann machine (RBM), which is also based on an Ising model. Note, however, that the

CellPress
OPEN ACCESS

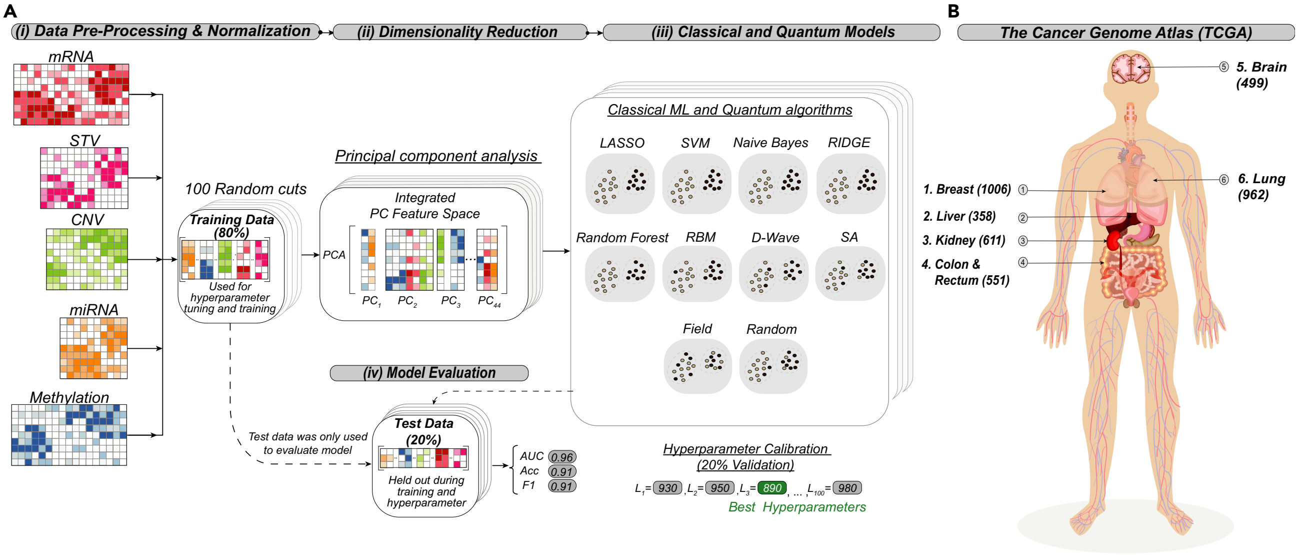

Figure 1. Overview of strategy and cancer types used in this study

(A) Overview of classification strategy. (i) Whole-exome sequencing, RNA-seq, miRNA-seq, DNA methylation array, and genotyping array (for CNVs) data were retrieved from The Cancer Genome Atlas for human cancer type and molecular subtype classification. Data were concatenated and transformed into a single scaled omics data matrix. The matrix was then repeatedly split into 100 unique training and independent test sets representing 80% and 20% of the total data, respectively. After the data were split, each training split was scaled to have zero mean and unit standard deviation. The same scaling was then applied to the corresponding test split. (ii) Principal-component analysis (PCA) was performed separately on each individual training set, and a subsequent matched test set was projected using training-set-specific PCA loadings. (iii) Several standard classical machine-learning (ML) algorithms were compared with quantum annealing and several classical algorithms that have the same objective function as quantum annealing. The standard classical ML methods assessed included least absolute shrinkage and selection operator (LASSO), ridge regression (RIDGE), random forest, naive Bayes, and support vector machine (SVM). Quantum annealing (D-Wave) was performed on D-Wave hardware by formulating the classification problem as an Ising problem (see experimental procedures). These classical Ising-type approaches include simulated annealing (SA), candidate solutions randomly generated and sorted according to the Ising energy (Random), and an approach that considers only local fields of the Ising problem (Field). Hyperparameters were tuned on the train data using a 10-fold cross-validation (see supplemental experimental procedures for a description of the ranges of hyperparameters used). (iv) After training, classification performance was validated with each corresponding test set (unseen during the tuning of hyperparameters and the training) for a variety of statistical metrics, including balanced accuracy, area under the ROC curve (AUC), and F1 score. Classification performance metrics were averaged for the 100 test sets for each model to provide statistics on the mean performance.

(B) The six human cancer types used for the multiclass classification models. Patient sample sizes are indicated in parentheses.

annealing-based Ising models described above are purely supervised learning approaches, i.e., formulated explicitly with a response variable to be predicted (in this case, the class of cancer). Boltzmann machines generally seek to explicitly model a data distribution over the inputs as a Boltzmann distribution by incrementally adjusting the $\boldsymbol{h}$'s and $\boldsymbol{J}$'s, whereas in our formulation the $\boldsymbol{h}$'s and $\boldsymbol{J}$'s are fixed given the input training data, and the mechanism of learning is to obtain solutions that minimize the Ising Hamiltonian, rather than to accurately reproduce a distribution. Accordingly, we will use "all Ising models" to refer to both the annealing-based Ising approaches and the RBMs, and "annealing-based" Ising approaches to refer exclusively to the annealing-based approaches.

Binomial and multinomial classification

In this section, we present classification results for five binomial TCGA cancer dataset comparisons: kidney renal clear cell carcinoma (KIRC) versus kidney renal papillary cell carcinoma (KIRP), lung adenocarcinoma (LUAD) versus lung squamous cell carcinoma (LUSC), breast invasive carcinoma (BRCA) versus matched normal breast tissue (normal), estrogen receptor positive (ERpos) versus estrogen receptor negative (ERneg) breast cancers, and luminal A (LumA) versus luminal B (LumB) breast cancers. We also present findings relative to a six-cancer multiclass classification strategy for human brain, breast, kidney, lung, liver, and colorectal cancer types (see Table S1 for the sample sizes of each dataset). We assessed the relative classification performance of the five standard ML models (LASSO, Ridge, RF, NB, and SVM), one quantum algorithm (D-Wave), three annealing-based Ising algorithms (SA, Random, and Field), and one Ising model (RBMs) for all binomial and multiclass TCGA comparisons.

Figure 2 presents comparisons of all 10 classifiers for the five binomial datasets. We used four statistical metrics to assess classification performance: accuracy, balanced accuracy, receiver operating characteristic (ROC) area under the curve (AUC), and F1 score. The four metrics were independently averaged over 100 unique training and test sets for each classifier (see supplemental experimental procedures). The mean ± SEM for each metric are presented on the y axis of each figure inset. See Table 1 for the values of the balanced accuracy and Tables S2–S4 for accuracy, AUC, and F1 score. Relative classification performance was determined by mean balanced accuracy and presented in ranked order on the x axis of each figure inset. Nonparametric Wilcoxon signed-rank tests were used to assess statistical significance among the 1010 classifiers relative to the

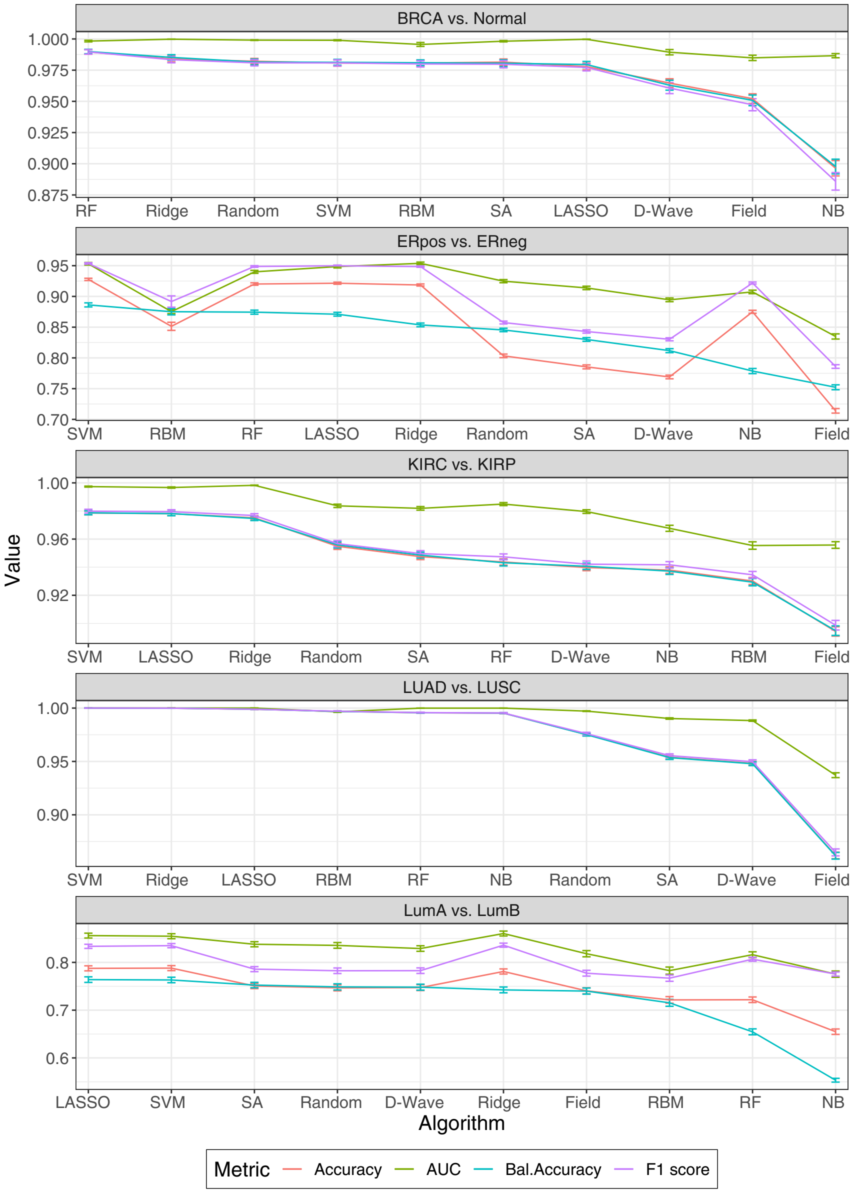

Figure 2. Comparison of classification algorithms for five TCGA cancer datasets

Human cancer datasets assessed: breast invasive carcinoma (BRCA) versus matched normal tissue (normal), estrogen receptor positive (ERpos) versus estrogen receptor negative (ERneg) breast cancers, kidney renal clear cell carcinoma (KIRC) versus kidney renal papillary cell carcinoma (KIRP), lung adenocarcinoma (LUAD) versus lung squamous cell carcinoma (LUSC), and luminal A (LumA) versus luminal B (LumB) breast cancers. To address class imbalance for each comparison, algorithm performance is ranked by mean balanced accuracy on the x axis. By and large, the other metrics indicate the same performance ranking. Classification performance metrics were averaged for the 100 unique training and test sets for each model (see experimental procedures). Performance metrics: accuracy (red), AUC (green), balanced accuracy (blue), and F1 score (purple). Data are presented as the mean ± SEM.

four performance metrics. Bonferroni correction was used to adjust for multiple testing error. For each comparison, we found that a standard ML approach outperformed both quantum and classical annealing across all four metrics of performance. However, for several comparisons, at least one of the Ising-type algorithms performed nearly as well as the best classical method. For example, while RF statistically outperformed (0.99 ± 0.002) all other methods for the BRCA versus normal comparison, Random, SVM, RBM, SA, and LASSO showed no statistical differences in performance (0.98 ± 0.002; 0.98 ± 0.002; 0.98 ± 0.002; 0.98 ± 0.003; 0.98 ± 0.002). Similarly, for the LumA versus LumB comparison, we found that LASSO performed best (0.76 ± 0.006); however, Random, D-Wave, SA, Ridge, and Field were nearly identical in terms of balanced accuracy (0.75 ± 0.006; 0.75 ± 0.006; 0.75 ± 0.006; 0.74 ± 0.006; 0.74 ± 0.006). For the three other comparisons (ERpos versus ERneg, KIRC versus KIRP, LUAD versus LUSC), the annealing-based Ising algorithms statistically underperformed versus the best standard ML algorithm in each comparison, although RBM performed well. While D-Wave performed similar to RF and NB in the KIRC versus KIRP comparison (0.94 ± 0.002 versus 0.94 ± 0.002; 0.94 ± 0.002; corrected p = 1), it was statistically inferior to SVM (0.94 ± 0.002 versus 0.98 ± 0.001; corrected p = 5.96 × 10^{-24}). Overall, Field was one of the poorest performing methods relative to the four metrics assessed; however, it performed relatively well on the LumA versus LumB dataset (0.74 ± 0.006). The quantum and classical Ising-type classification results indicate the utility of framing an overall classification strategy as an Ising problem.

Although the Ising-type algorithms generally underperformed versus the standard ML methods assessed for these comparisons, the Ising-type classifiers performed well on the LumA versus LumB comparison. Moreover, as with all the standard ML methods used in this work, the most informative feature for classification predicted by the annealing-based Ising models was the first PC, indicating that the Ising models also assigned the greatest weight to the features that account for the most variation within the data. This is consistent with previous results where D-Wave was able to extract a motif for protein-DNA binding that agreed with classical results.[18]

Finally, to determine the utility of Ising-type methods on a larger, multiclass classification experimental design, we evaluated classification performance of the standard and Ising-type ML algorithms on a six-cancer, multiclass TCGA dataset. The six TCGA cancer types included brain, breast, kidney, lung, liver, and colorectal cancers (see Table S1 for the sample size of this six-cancer dataset). With the exception of multiclass AUC

Table 1. Balanced accuracies for five binomial comparisons and the one six-class cancer dataset used in this study

Dataset	LASSO	Ridge	SVM	RF	NB	D-Wave	SA	Random	Field	RBM
BRCA versus normal	0.982 ± 0.002	0.984 ± 0.002	0.981 ± 0.002	0.990 ± 0.002	0.897 ± 0.006	0.974 ± 0.003	0.981 ± 0.003	0.982 ± 0.002	0.951 ± 0.004	0.981 ± 0.002
ERpos versus ERneg	0.871 ± 0.003	0.854 ± 0.003	0.886 ± 0.003	0.874 ± 0.003	0.779 ± 0.004	0.812 ± 0.003	0.830 ± 0.003	0.845 ± 0.003	0.752 ± 0.004	0.875 ± 0.005
KIRC versus KIRP	0.978 ± 0.002	0.975 ± 0.002	0.979 ± 0.001	0.944 ± 0.002	0.937 ± 0.002	0.947 ± 0.002	0.949 ± 0.002	0.956 ± 0.002	0.895 ± 0.003	0.929 ± 0.002
LUAD versus LUSC	0.9988 ± 0.0002	0.9999 ± 0.0001	1.0000 ± 0.0000	0.9957 ± 0.0004	0.9953 ± 0.0004	0.9478 ± 0.0017	0.9536 ± 0.0017	0.9751 ± 0.0013	0.8616 ± 0.0031	0.997 ± 0.0004
LumA versus LumB	0.764 ± 0.006	0.742 ± 0.006	0.763 ± 0.006	0.655 ± 0.006	0.553 ± 0.004	0.748 ± 0.006	0.752 ± 0.006	0.749 ± 0.006	0.740 ± 0.006	0.715 ± 0.007
Six cancer	0.9896 ± 0.0002	0.9845 ± 0.0003	0.9891 ± 0.0002	0.9863 ± 0.0003	0.9735 ± 0.0004	0.9122 ± 0.0012	0.9170 ± 0.0010	0.9083 ± 0.0015	0.8876 ± 0.0005	0.9534 ± 0.0013

Data are reported as the mean ± SEM.

(0.99 ± 0.0), performance metrics for standard ML approaches were superior to those of all Ising models for this larger, multiclass dataset (see also Figure S1). We, therefore, focused our efforts on further evaluating the efficacy of all the Ising models on the five binomial comparisons described above.

Performance dependence on training set size

Based on previous work indicating that quantum and classical Ising-type approaches are superior to standard ML classifiers on small training set sizes,[18,32,33] we systematically reduced the training set data for the LumA versus LumB human breast cancer comparison into 16 separate partition sizes to evaluate classifier performance (see supplemental information). We first divided the entire LumA versus LumB breast cancer dataset (311 breast tumor samples) into a training set representing 80% of the initial dataset (250 breast tumor samples) and a testing set equal to 20% of the initial dataset (61 breast tumor samples). From this, we randomly selected incrementally smaller, class-balanced data partitions from 95% to 20% of the original training set data. Due to the complexity and computational expense of this experimental design, we trained each of the 10 classifiers described above over only 50 unique training sets randomly drawn from the 250 breast tumor samples of the initial training data, for each training set partition. We then validated the performance of each classifier on the original, held-out test set of 61 breast tumor samples. As above, nonparametric Wilcoxon signed-rank tests were used to assess statistical significance among the 10 classifiers relative to the four performance metrics, and Bonferroni correction was used to adjust for multiple testing error. The results in Figure 3 are presented as the mean ± SEM for averaged balanced accuracies across the entire training set size spectrum; see Table 2 for the balanced accuracies of all the algorithms at some representative training fractions.

At 25%–40% of the original training data (63 to 100 breast tumor samples), the mean balanced accuracies of the five Ising models (D-Wave, SA, Random, Field, and RBM) were statistically superior to the mean balanced accuracies of the five standard ML algorithms (LASSO, NB, RF, Ridge, SVM). For example, at 25% of the initial training data D-Wave statistically outperformed SVM, the top standard ML method (0.74 ± 0.007 versus 0.70 ± 0.007; corrected $p = 1.94 \times 10^{-3}$), as did the other Ising-type methods. Classification performance for all standard ML methods (SVM, LASSO, NB, RF, Ridge) steadily decreased after a reduction to 50% of the original training data (125 breast tumor samples), whereas we found significantly less reduction in mean balanced accuracies for the five quantum and classical Ising models across the entire training set size spectrum. Furthermore, the Ising models showed a relatively minimal reduction in performance at 95% versus 20% of the original training data (0.76 ± 0.004 versus 0.73 ± 0.007; corrected $p = 0.092$ for SA's performance) compared with LASSO (0.75 ± 0.002 versus 0.60 ± 0.01; corrected $p = 3.32 \times 10^{-11}$). Moreover, all five standard ML methods were associated with a significantly higher degree of overfitting than the Ising model classification approaches, an issue that has also historically plagued the analysis of genomic data. Figure S2A indicates significantly less statistical shrinkage relative to test data for the Ising model algorithms across all fractions of training data for the LumA versus LumB comparison. As an

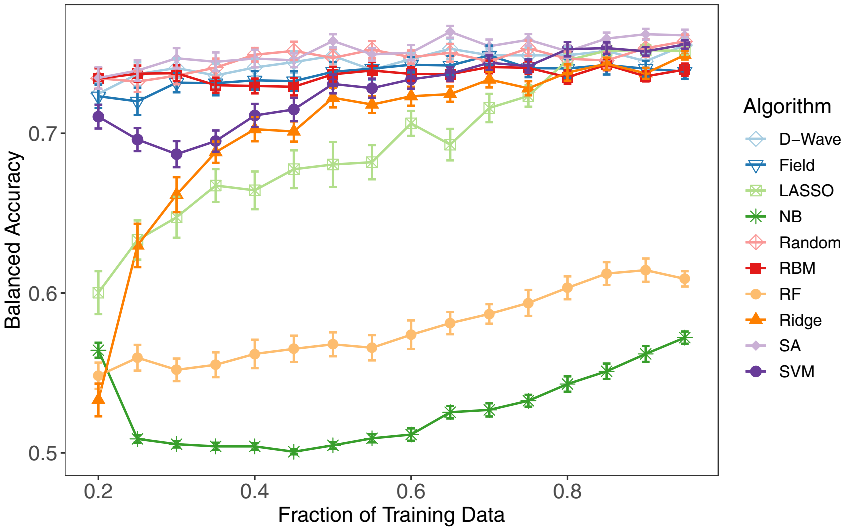

Figure 3. Test set balanced accuracy for LumA versus LumB binomial classification with incremental decreases from 95% to 20% of the original training set

The algorithms evaluated are indicated in the legend. Averaged balanced accuracies were calculated for 50 independent training sets at each designated fraction of the original training data. Data are presented as the mean ± SEM.

example, with 20% of the training data, although RBM and SVM perform fairly similarly in terms of the balanced accuracy on the test set (0.73 ± 0.007 versus 0.71 ± 0.007, $p = 0.016$), the overfitting, as measured as the difference between the training and the test balanced accuracy, is significantly higher for SVM than for RBM (0.29 ± 0.007 versus 0.17 ± 0.007, $p = 9.37 \times 10^{-14}$).

To assess the generality of this finding that Ising-type methods may perform better than standard ML approaches with a small amount of training data, we performed the same analysis on the ERpos versus ERneg breast cancer and the six-cancer, multiclass datasets. As both datasets were significantly larger than the LumA versus LumB comparison, we reduced each to a much smaller percentage of the initial training set size. Figure S3A presents mean balanced accuracies from 95% to 10% of the original training data (730 to 77 breast tumor samples) for the ERpos versus ERneg comparison. We found the same result in classification performance for all 10 classifiers; namely, a decrease in performance for the small training data for the standard ML methods, but little or no change for the Ising models. Unlike the LumA versus LumB comparison, the Ising models showed no statistical loss in performance from 95% to 10% of the original training data (0.84 ± 0.004 versus 0.84 ± 0.002; corrected $p = 1$ for SA); whereas RF dropped from 0.86 ± 0.002 to 0.81 ± 0.008 (corrected $p = 1.13 \times 10^{-5}$). Similar to the LumA versus LumB comparison, Figure S2B indicates that the Ising models generally have less overfitting across many of the training fractions; SVM had a higher degree of overfitting compared with SA (0.14 ± 0.006 versus 0.02 ± 0.005, $p = 8.48 \times 10^{-17}$).

Analysis of the six-cancer, multiclass dataset further confirmed the ERpos versus ERneg findings. While Figure S3B shows that the standard ML methods significantly outperformed the Ising-type methods, here again we found no statistical reduction in D-Wave performance (0.92 ± 0.001 versus 0.91 ± 0.002; corrected $p = 1$) from 95% (3,035 tumor samples) to 5% (163 tumor samples) of the initial training set size, although RBMs did better than the annealing-based Ising approaches. Comparatively, we again found a significant reduction in classification performance for LASSO (0.992 ± 0.0001 versus 0.978 ± 0.001; corrected $p = 9.89 \times 10^{-16}$) on this multiclass cancer dataset. In addition, SA also exhibited a significant performance drop relative to D-Wave at the low end of the training data fraction, although this feature is temperature dependent: by modifying SA's final temperature it can be made to perform as well as D-Wave. This is concordant with previous binomial quantum ML studies.[30,31]

In summary, all methods (with the exception of NB) converged to roughly the same balanced accuracy at a high training data fraction, but at a low fraction all Ising models performed better on three distinct datasets. These findings go beyond previous work[30,31] and further bolster the case for the utility of framing an overall classification strategy as an Ising problem. Moreover, robust classification of small, high-dimensional omics datasets with Ising models provides a potential new avenue to evaluate patient response in the early phase of clinical drug trials or in other genome-wide datasets with relatively small numbers of patients or animal models.

Gene-level classification

To assess the performance of the Ising-type methods at the gene level, we used the 44 most informative genes, by PCA loading of the first PC (PC1), from the original training set described in the previous sections for the LumA versus LumB breast cancer dataset. Results are presented in Figure 4A. The four metrics were independently averaged over 100 unique training and test sets for each of the 10 classifiers. Nonparametric Wilcoxon signed-rank tests were again used to assess statistical significance for the four metrics relative to the 10 classifiers. As above, Bonferroni correction was used to adjust for multiple testing error. Here we found a significant increase in mean balanced accuracies for all 10 classifiers at the gene level compared with PCA feature-based classification. For example, RF performed significantly better at the gene level compared with the PC level (0.83 ± 0.007 versus 0.65 ± 0.008; corrected $p = 3.02 \times 10^{-31}$). We also found that Random (0.81 ± 0.005), SA (0.80 ± 0.005), and D-Wave (0.80 ± 0.006) slightly outperformed three of the five standard ML approaches: SVM (0.79 ± 0.005), NB (0.79 ± 0.006), and LASSO (0.77 ± 0.005). To confirm the multi-omics PCA-derived gene-level classification findings, we performed a simple dual-dimensionality reduction and differential analysis approach on the LumA versus LumB comparison with edgeR.[34] Briefly, edgeR fits a negative binomial distribution to assess whole-transcriptome gene expression. In this second

Table 2. Balanced accuracies when incrementally decreasing the amount of training for the LumA versus LumB comparisons

Fraction	LASSO	Ridge	SVM	RF	NB	D-Wave	SA	Random	Field	RBM
0.25	0.633 ± 0.012	0.630 ± 0.013	0.696 ± 0.007	0.560 ± 0.008	0.508 ± 0.002	0.737 ± 0.007	0.739 ± 0.007	0.732 ± 0.007	0.720 ± 0.009	0.737 ± 0.005
0.55	0.682 ± 0.011	0.718 ± 0.005	0.728 ± 0.006	0.566 ± 0.008	0.509 ± 0.003	0.740 ± 0.006	0.749 ± 0.004	0.752 ± 0.005	0.740 ± 0.007	0.739 ± 0.005
0.95	0.752 ± 0.002	0.749 ± 0.003	0.756 ± 0.003	0.609 ± 0.005	0.572 ± 0.004	0.755 ± 0.004	0.761 ± 0.004	0.757 ± 0.004	0.739 ± 0.005	0.740 ± 0.004

The fraction represents the amount of training data used, and results are reported as the mean ± SEM.

analysis, the top 44 differentially expressed mRNAs were used for gene-level classification in the same manner as described above. Given that edgeR gene-level classification was comparable to PCA gene-level findings (Figure S4), we used the features from PC1 to take advantage of the enhanced molecular information content of our multi-omics approach.

Close inspection of the top 44 genes from PC1 used as molecular features for the LumA versus LumB comparison indicated that *RACGAP1* was the most informative feature, as averaged across 9 of the 10 classifiers (see Table S5; RBMs were not included because of the difficulty in assessing feature importance). This finding was further supported via an independent edgeR[34] analysis, which showed that *RACGAP1* was the strongest differentially expressed gene (false discovery rate [FDR] = 2.57×10^{-36}; logFC = -1.11) of the top 41 mRNA genes. Figure S5 presents a rank-ordered heatmap of the averaged state for each of the 44 genes (41 mRNA and 3 methylated genes) across the 100 unique training sets for the LumA versus LumB comparison. Conversely, *RACGAP1* was ranked only 22 of 44 by PC1 loading. These findings indicate the importance of combined dimensionality reduction/feature learning and classification of high-throughput biological data. From a biological perspective, *RACGAP1* is a putative oncogene, which promotes the growth of triple-negative/basal-like breast cancers. Experimental depletion of this gene inhibits cancer cell proliferation by the combined effects of cytokinesis failure, *CDKN1A/p21*-mediated *RB1* inhibition, and the onset of senescence.[35] Given the significant increased expression of *RACGAP1* in LumB tumors, the more aggressive breast cancer subtype, our gene-level classification results also support our previous findings indicating that Ising-type models are capable of robustly assigning the greatest weight to the most biologically relevant information in a given model. Figure 4B shows hierarchical clustering of the 44 most informative genes for the LumA versus LumB breast cancer comparison and indicates significant discrimination between LumA and LumB based on these 44 genes.

Finally, we used GOseq analysis[36] and a PubMed Central (PMC) comprehensive semantic search engine to determine the known biological relevance of the top 44 genes in the LumA versus LumB breast cancer comparison. Our GOseq analysis produced 244 functionally enriched gene ontology (GO) terms (see Table S6). Of these, Figure 4C presents nine statistically significant (Wallenius approximation; FDR $\leq$ 0.05) GO terms related to cancer: metabolic process, cell cycle, heterocycle metabolic process, regulation of the cell cycle, glucose 6-phosphate metabolic process, DNA integrity checkpoint, telomere organization, and morphogenesis of a branching epithelium. We then used a semantic search engine to query full-text records available in PMC database for published relationships between these 44 genes and the query terms *cancer* and *breast cancer* (see supplemental experimental procedures). Briefly, we used the entrez search function of the rentrez R package, which provides an NCBI EUtils application programming interface,[37] to retrieve results for each of the 44 genes from the PMC database. Search terms were defined by combining each gene symbol with either *cancer* or *breast cancer* fields, along with all related MeSH terms using the Boolean operators AND/OR.

We found that all but *C12orf73* have been previously indicated in breast cancer (Figure 4D). Of the remaining 43 genes, *PRR15L* and *MAGI2-AS3* are the only genes with no current functional

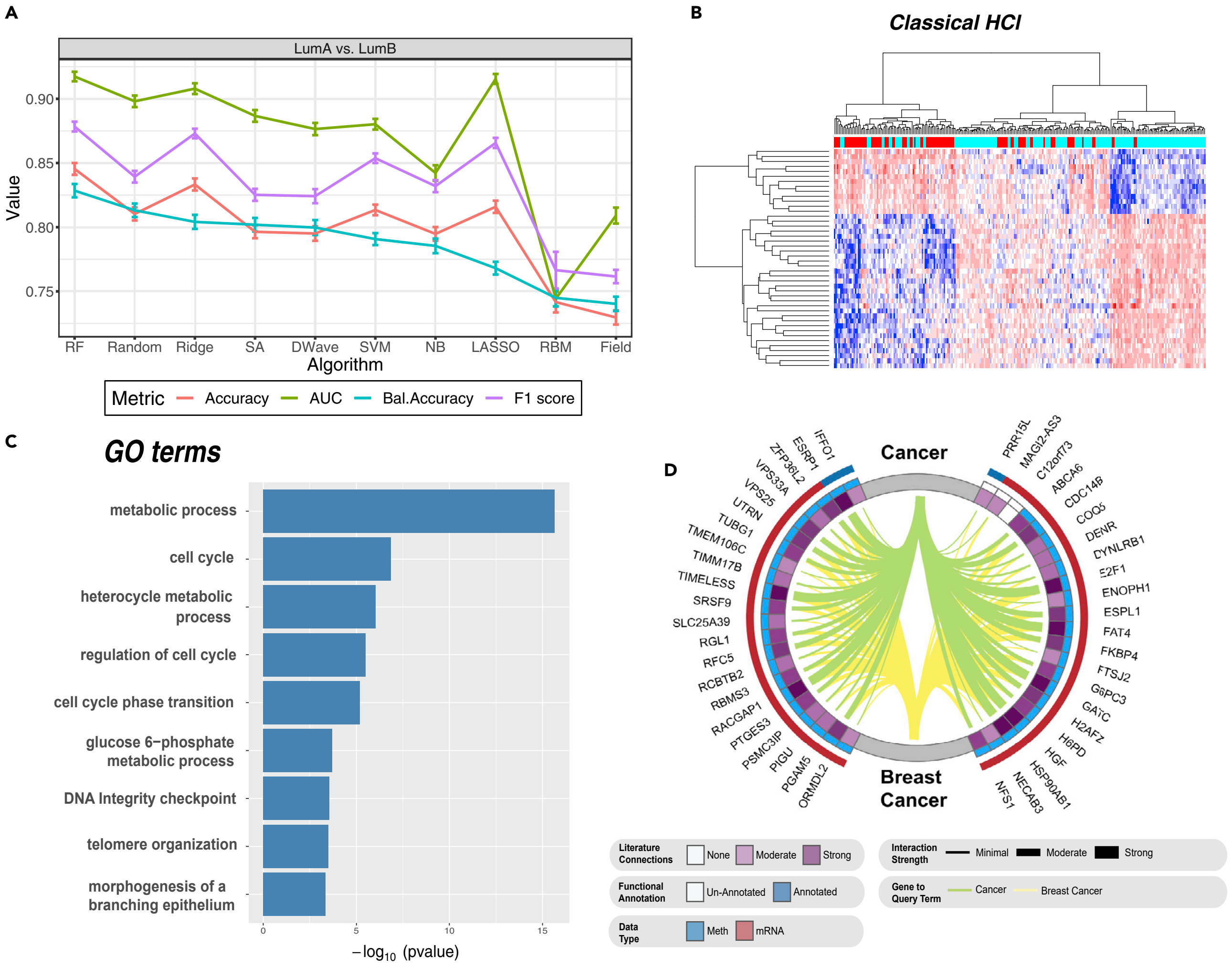

Figure 4. Classification, hierarchical clustering, functional enrichment, and natural language processing of the top 44 genes of PC1 for LumA versus LumB binomial comparison

(A) Gene-level classification of LumA versus LumB human breast cancers based on the top 44 genes of PC1. Data are presented as the mean ± SEM.

(B) Classical hierarchical clustering algorithm (see experimental procedures). Note: genes are presented in rows and samples in columns.

(C) GOseq functional enrichment analysis of the top 44 genes for PC1 shows enriched GO terms ordered by p value.

(D) Circos plot representing semantic search of full-text articles within the PubMed Central database identifying published associations of the top 44 genes for PC1 to the query terms *cancer* and *breast cancer*. The red and blue outer bands represent "mRNA" and "methylation" data types, respectively. The inner blue band represents genes with known functional annotation. The intensity of the inner purple ring indicates the total number of publications on cancer and breast cancer for the top 44 genes of PC1. This band has six colored bins, where white is the lowest and dark purple the highest number of publications at the time of analysis. The thickness and color of the Circos plot ribbons indicate the number of published gene-to-query term associations: green represents cancer and yellow designates breast cancer.

annotation; however, both *PRR15L* and *MAGI2-AS3* associate with a high averaged information ranking for the LumA versus LumB comparison (see Table S5). At the time of our semantic search of the PMC database, hepatocyte growth factor (*HGF*) and retinoblastoma-associated protein 1 (*E2F1*) were implicated in the greatest number of published breast cancer papers (6,356 and 5,925, respectively) among all of the 44 genes queried (see Table S7). *E2F1* yielded higher PC1 loading (4 versus 15) and averaged information (8.6 versus 33) rankings than *HGF*. *E2F1* is a well-studied transcription factor involved in cell proliferation, differentiation, and apoptosis. It is a member of the E2F protein family, which has been implicated in cell-cycle control and regulation of tumor suppressor proteins. Low *E2F1* gene expression is predictive of metastasis-free survival in breast cancer patients.[38] As with our *RACGAP1* finding, we determined significantly higher differential mRNA expression of *EF2F1* in LumB versus LumA breast cancers via edgeR analysis (FDR = 2.59×10^{-27}; logFC = −1.34). Taken together, our gene-level classification results support known breast cancer etiology.

DISCUSSION

We have presented the first successful demonstration of annealing-based Ising models applied to integrated genome-wide

multi-omics human cancer data. We have shown that classification with Ising models is comparable to standard ML strategies on multiple partitions of data of multiple large human cancer datasets. However, it is important to note that the benefit of using quantum annealing cannot be attributed solely to inherent quantum behavior, as SA and our Random control classifier performed similar to, if not better than, quantum annealing as implemented by a D-Wave device on two of the three fractional training dataset comparisons. By randomly generating bit strings and sorting them by their Ising energy, we achieved classification accuracies nearly equal to standard ML and, in some cases, better than both quantum and SA. The comparable performances of our random control strategy and D-Wave and SA are due to a distinction between the objective function for the Ising-type approaches, which is an approximation for the negative log likelihood, and the performance metrics presented (accuracy, balanced accuracy, F1 score, AUC). While we describe this discrepancy in more detail in Figure S6 and Note S1, we found the overall classification performance of the random classifier to be a direct indication of the utility of formulating a classification problem as an Ising Hamiltonian. In this study, the advantage of using an Ising problem became even more apparent by training classifiers on a relatively smaller amount of training data, as we witnessed with the LumA versus LumB and ERpos versus ERneg breast cancer comparisons. For example, Field, which is an almost trivial algorithm after formulating the Ising problem, performed extremely well from 95% to 20% of the original training data for this breast cancer comparison.

The relative advantage of annealing-based Ising approaches over standard ML approaches when trained with relatively small amounts of data may be attributed to the discrete weights returned for the Ising-type methods. On one hand, discrete weights rendered with Ising-type methods control for statistical shrinkage better than statistical optimization parameters of standard ML approaches. This generalizability issue has plagued the ML field for decades. On the other hand, binary weights limit the informativeness of the standard classifiers; with larger amounts of training data, the Ising-type methods slightly underperformed standard ML approaches. These findings point to the potential application of a new class of algorithms, as simple heuristic models with discrete weights may perform better in situations of limited training data, which is often the case in clinical trials and drug efficacy studies. The relative advantageous trend of enhanced classification performance for Ising-type methods on small amounts of training data is true even when using gene-level features; Figure S7 shows balanced accuracies for LumA versus LumB and ERpos versus ERneg breast cancer comparisons relative to the top 44 genes from PC1 on incrementally smaller amounts of training data. Interestingly, the gene-level RBM underperforms all other Ising-type methods at all but the smallest fraction of training data. Moreover, Figure S8 shows statistically enhanced control of overfitting for Ising-type methods, especially at low fractions, on both LumA versus LumB and ERpos versus ERneg comparisons.

Although RBMs are formulated quite different from the annealing-based Ising models, in that RBMs are unsupervised and iteratively update model parameters, whereas the annealing-based approaches explored here are supervised with fixed $\boldsymbol{h}$'s and $\boldsymbol{J}$'s, the overall trends in classification performance for all these Ising models are quite similar. When using very small amounts of training data, RBMs also seem to perform much better than the standard ML methods, although their performance is noticeably better on the PCs than on the gene-level features. This may be attributed, in part, to distinct underlying data distributions; different algorithms do better on certain types of data. For example, Field, which performed quite well for the LumA versus LumB comparison on the PC-level data, performed poorly on the gene-level data, while RF performed better at the gene-level than the PC-level data. Differences in data type notwithstanding, the reason RBMs are performing well with small amounts of data may also be the binary nature of the hidden units. Although there are no "weights" to be learned, as there are with the annealing-based approaches, the hidden units for the RBMs were binarized during training; it is possible that this intentional sacrifice of precision leads to less overfitting with relatively smaller amounts of data.

Inherent to all useful biological classifiers, we showed that all the Ising-type algorithms identified relevant molecular features in each cancer comparison. Like the standard ML approaches, these algorithms determined PC1 as the most informative feature for each dataset, from which we then proceeded to perform gene-level classification. Analysis of feature importance of the trained classifiers on the top 44 genes of PC1 for the LumA versus LumB comparison determined *RACGAP1*, a putative oncogene in breast cancer, to be associated with the highest averaged information ranking. This finding was supported via independent differential gene expression analysis, indicating that LumB tumors, a more aggressive molecular subtype of breast cancer, were associated with statistically significant, higher mRNA levels of *RACGAP1* than LumA tumors. Moreover, our semantic search of full-text records available in the PMC database found that 43 of these top 44 genes have been previously implicated in breast cancer. While our results support previously published findings, it is possible that more sophisticated dimensionality-reduction techniques, such as multi-omics factor analysis,[39] could be used to provide fresh insights into the mechanisms of disease. The effect of such techniques on the relative performance of both standard and Ising-type ML methods is worthy of further study.

While we achieved comparable classification performance on all binomial comparisons assessed in this study, it is important to note that our Ising-type approaches did not perform as well as standard ML on a large multiclass, six-cancer dataset. This observation is most likely related to the relatively larger training dataset used for this multiclass comparison, as the six-cancer dataset comprised approximately 12 times the amount of data relative to the LumA versus LumB dataset. As we showed by reducing the amount of training data for the LumA versus LumB, the ERpos versus ERneg, and the six-cancer multiclass comparisons, the Ising-type approaches performed well with relatively smaller amounts of data but did not statistically improve with incremental increases. Another explanation for the decreased performance of the Ising-type approaches may be related to the fact that the number of approximations used to formulate the classification problem as an Ising Hamiltonian depends on the number of classes (see supplemental experimental procedures, Equation 15). The approximation may be valid for binomial comparisons but could break down with

multiclass experimental designs. In contrast, the RBMs, which are not formulated based on the same approximations, perform significantly better than the annealing-based Ising models.

Although practical quantum computing architectures are still in development, the demonstration of classification performances of Ising-type approaches that are comparable to standard ML methods on high-dimensional, multi-omics human cancer datasets is encouraging. Our survey of ML classifiers has uncovered a class of algorithms that perform better than standard methods on limited biomedical data: Ising-type methods with discrete weights. This advantage for small experimental designs is particularly useful in medicine, where large datasets may be prohibitively expensive to obtain, or in the study of rare diseases. As technology improves and new algorithms are introduced, we are cautiously optimistic that these unconventional classification algorithms will afford unique insights and drive the discovery of novel approaches for solving complex biological problems.

EXPERIMENTAL PROCEDURES

Resource availability

Lead contact

Tom Chittenden may be contacted for additional information (email:tom.chittenden@genuitysci.com).

Materials availability

No new materials were generated in this study.

Data and code availability

The processed data that supports the findings of the study is available at Mendeley Data: https://doi.org/10.17632/thjjpv3df3. The code that supports the findings of this study is available via https://github.com/Genuity-Science/unconventionalML.

Methods

Dataset and dimensionality reduction

Genomic data from TCGA were retrieved, preprocessed, and normalized. An overview of our data pipeline is depicted in Figure 1. Briefly, we retrieved whole-exome sequencing, RNA-seq, miRNA sequencing, DNA methylation array, and genotyping array data for five human cancer binomial classifications (breast cancer versus normal, ERpos versus ERneg breast cancers, LumA versus LumB breast cancers, kidney renal clear cell versus papillary cell carcinoma, and LUAD versus squamous cell carcinoma), as well as a six-cancer multiclass classification, which included breast, colorectal, lung, kidney, brain, and liver cancer types. Data were retrieved from either the Genome Data Commons data portal (https://portal.gdc.cancer.gov/, data release 4.0) or cBioportal (http://www.cbioportal.org/).[40,41] All five data types (mRNA, miRNA, CNV, DNA methylation, and somatic tumor variants) were preprocessed independently (see supplemental experimental procedures) and then concatenated into a single data matrix.

We derived classification performance via 100 random, approximately class-balanced partitions of training (80%) and test/validation (20%) data. Each feature was standardized to zero mean and unit variance (Z score) based on the training data. The same training mean and standard deviation was then applied to the corresponding test data. Furthermore, given that the data comprised more than 79,000 molecular features, dimensionality reduction was conducted in order to make comparisons with existing quantum hardware. As such, we performed PCA on each random, balanced partition of the training data, retaining the top 44 PCs for the binomial datasets and 13 PCs for the six-cancer dataset. The test data were then projected onto the PCs defined by the corresponding training data. The number of PCs was chosen based on the largest number of features that could be accommodated on existing quantum annealing hardware (see the section below on formulating the classification problem as an Ising model). Hyperparameters were selected using cross-validation on the training data (see supplemental experimental procedures for more information about which hyperparameters were chosen).

Quantum annealing

We explored the use of quantum annealing with processors made by D-Wave Systems, Inc.[11,12] (see the supplemental experimental procedures for a brief review of quantum annealing). Results for the binomial datasets were obtained by running the D-Wave 2X (DW2X) processor installed at the Information Sciences Institute (ISI) of the University of Southern California, and results for the six-cancer dataset were run on the DW2000Q located in Burnaby, British Columbia, Canada. The problem Hamiltonians that are used for D-Wave can be described as Ising spin models with tunable parameters.[11] The Ising model assumes a graph $\mathcal{G} = (V, E)$ composed of a set of vertices, V, and edges, E. Each of the spins is a binary variable located at a unique vertex. The spins are represented by N superconducting flux qubits, and G is the so-called Chimera graph (see Figure S9). For the DW2X, N = 1,098, and for the DW2000Q, N = 2,038. The problem (or Ising) Hamiltonian for this system can be written as:

$$H_P = \sum_{i \in V} h_i \sigma_i^z + \sum_{(i,j) \in E} J_{i,j} \sigma_i^z \sigma_j^z, \qquad \text{(Equation 1)}$$

where the local fields $\{h_i\}$ and couplings $\{J_{ij}\}$ define a problem instance and are programmable on the DW2X to within a few percent Gaussian distributed error. The $\{\sigma_i^z\}$ represent both binary variables taking on values ±1 and the Pauli z matrices. Given a spin configuration $\{\sigma_i^z\}$, H_p is the total energy of the system. Problems submitted to D-Wave are automatically scaled so that all h_i and J_{ij} values lie between -1 and 1. The initial Hamiltonian $H_B = \sum \sigma_i^x$ is a transverse magnetic field where σ_i^x is the Pauli x matrix acting on qubit i. During an anneal, the magnitude of H_B is gradually reduced to zero, while the magnitude of H_p is slowly increased from zero. After each anneal D-Wave returns a set of spin values $\{\sigma_i^z = \pm 1\}$ that attempts to minimize the energy given by Equation 1 (a lower energy indicates better optimization). Note, however, that for our purposes we are not strictly using D-Wave as an optimizer. In the supplemental experimental procedures, we describe our procedure to make use of the fact that higher-energy solutions may still contain some meaningful information and use them to improve performance.

For the results in the main text, we set the annealing time at 5 μs and repeated the anneal 1,000 times, which returns 1,000 spin configurations. We selected the 20 spin configurations with the lowest Ising energy and ran some quick classical postprocessing to average the lowest Ising energy spin configurations if they improved the objective function on the training data. See the supplemental experimental procedures for a more detailed description of other hyperparameters and Figures S10–S12 and Note S2 for the effect of using a larger number of spin configurations.

Simulating annealing

Similar to quantum annealing, SA accepts problems formulated as an Ising problem, as defined in Equation 1, and returns binary variables. For this work we used the implementation of Isakov et al.[42] There are several important parameters that affect SA's overall performance: the number of sweeps, the type of schedule (linear or exponential in the inverse temperature), and the initial and final temperatures. For our purposes, we fixed the number of sweeps (which is analogous to the annealing time of quantum annealing) to 1,000 and selected a linear schedule with an inverse initial temperature of 0.01. We treated the final inverse temperature as a tunable hyperparameter with values in the set $\{0.03, 0.1, 0.3, 1, 3\}$ and repeated the anneal 1,000 times. Results in the main text are given for the final inverse temperature that yielded the best performance during cross-validation. We used the same classical postprocessing procedure that was used with D-Wave to combine 20 spin configurations with the lowest energy, not just the one that returned the lowest Ising energy.

Field

As another approach to explore the usefulness of the formulating the classification task as an Ising problem, and to check the role played by the couplings (the $\boldsymbol{J}$'s), we implemented a very simple algorithm that takes into account only the values of the local fields (the $\boldsymbol{h}$'s) in Equation 1. Once h has been determined based on the training data, we chose the weights to be the opposite sign of the fields, i.e., $\sigma_i^{\text{field}} = -h_i$. This amounts to a (trivial) analytical solution of the optimization of Equation 1 without any $\boldsymbol{J}$'s.

Random

As a sanity check, we generated random solutions to Equation 1. For each spin we picked a random number uniformly distributed in the interval $[0, 1)$. Values

below 0.5 were set to -1 and those above 0.5 were set to 1, thereby generating spin configurations the same as those returned by D-Wave and SA. We then sorted the spin configurations according to their Ising energy, given by Equation 1. As with D-Wave and SA, we generated 1,000 such random spin configurations and used the same classical postprocessing procedure to combine the 20 spin configurations with the lowest energy to a final set of weights.

RBM

RBMs are a class of bipartite unsupervised energy-based models that consist of a visible layer (the data one would like to model) and a hidden layer. Introducing a hidden layer allows one to model more complicated probability distributions. An RBM defines a probability distribution through an energy function as $Pr(x) = \exp[-H(x)]/Z$, where $Z = \sum_x [-H(x)]$. The energy function for RBMs can be written as an Ising Hamiltonian, although the goal of learning is to update the $\boldsymbol{h}$'s and $\boldsymbol{J}$'s such that the probability distribution most closely resembles the input data distribution; in contrast, the $\boldsymbol{h}$'s and $\boldsymbol{J}$'s for the models above are fixed, given the training data.

To use RBMs for classification, we used the approach described in Larochelle et al.,[43] where class labels were added to the visible layer and the RBMs learned to model a distribution over both the "data" units and the "label" units, to adapt the R deepnet package. After training, the probability of each class was calculated by setting the corresponding label unit to 1 and all other label units to 0. The RBMs were trained with contrastive divergence using a $k = 1$ and a batch size of 32.

Formulating a multiclass classification problem on a quantum annealer

We show how to arrive at a simple Ising formulation to model a multiclass classification problem with K unique class labels. Assume we have a dataset of N training examples, $D = \{(\boldsymbol{x}_i, y_i)\}_{i=1}^N$, where $\boldsymbol{x}_i$ is the ith data vector of M features and y_i is the corresponding class of the ith data vector (i.e., y_i can take one of the K class labels). A simple way to arrive at probabilities for a multiclass classification problem is to use the softmax function. We can define the probability of each class as:

$$Pr(y_i = k) = \frac{\exp \boldsymbol{w}_k^\top \boldsymbol{x}_i}{\sum_{k=1}^K \exp \boldsymbol{w}_k^\top \boldsymbol{x}_i}, \quad \text{(Equation 2)}$$

where $\boldsymbol{w}_k$ are the weights corresponding to the kth class that we would like to learn (in other words, we define a set of weights for each class). However, since we are generating a probability of each class, we can reduce the set of weights we have to train from K to $K-1$ and define the first $K-1$ probabilities as:

$$Pr(y_i = k) = \frac{\exp \boldsymbol{w}_k^\top \boldsymbol{x}_i}{1 + \sum_{k=1}^{K-1} \exp \boldsymbol{w}_k^\top \boldsymbol{x}_i}, \quad \text{(Equation 3)}$$

with the probability of the Kth class as:

$$Pr(y_i = K) = \frac{1}{1 + \sum_{k=1}^{K-1} \exp \boldsymbol{w}_k^\top \boldsymbol{x}_i}. \quad \text{(Equation 4)}$$

The goal of training is to find the weights that maximize the probability given the classes in the dataset or, equivalently, to minimize the negative log likelihood. Once the weights are found, inference is straightforward; probabilities for each class are generated and we assign the predicted label based on the class with the highest predicted probability. We can express the negative log likelihood as follows:

$$\mathcal{L} = -\log \prod_i Pr(y_i) \quad \text{(Equation 5)}$$

$$= -\sum_i \log Pr(y_i), \quad \text{(Equation 6)}$$

where the probability selected corresponds to the actual class of the label. If the actual class has the highest predicted probability for all data samples, the negative log likelihood will be minimized. In other words, the further away from 1 the predicted probability of the real class is, the greater the contribution to the negative log likelihood; if the algorithm were able to correctly assign a class to each training example with probability 1, the negative log likelihood would be 0.

Taking a second-order Taylor approximation around the argument of the exponential equal to 0, we eventually arrive at the following expression for the negative log likelihood (see supplemental experimental procedures for a more complete derivation and additional technical concerns):

$$\mathcal{L} \approx \sum_{k=1}^{K-1} \boldsymbol{w}_k^\top (\boldsymbol{b}_k + \boldsymbol{h}) + \sum_{k=1}^{K-1} \boldsymbol{w}_k^\top \boldsymbol{J}' \boldsymbol{w}_k - \sum_{k=1}^{K-1} \sum_{j \neq k} \boldsymbol{w}_j^\top \boldsymbol{J}'' \boldsymbol{w}_k, \quad \text{(Equation 7)}$$

where

$$\boldsymbol{b}_k = \sum_{i:y_i = k} -\boldsymbol{x}_i, \boldsymbol{h} = \frac{1}{K} \sum_i \boldsymbol{x}_i, \quad \text{(Equation 8)}$$

$$\boldsymbol{J}' = \frac{K-1}{2K^2} \sum_i \boldsymbol{x}_i \boldsymbol{x}_i^\top, \boldsymbol{J}'' = \frac{1}{2K^2} \sum_i \boldsymbol{x}_i \boldsymbol{x}_i^\top. \quad \text{(Equation 9)}$$

In general, this formulation requires arbitrary interweight couplings (i.e., $\boldsymbol{J}''$– couplings between $\boldsymbol{w}_k$ and $\boldsymbol{w}_j$, where $\boldsymbol{w}_k$ and $\boldsymbol{w}_j$ represent the vector of weights for classes k and j) and intraweight couplings (J'– couplings between $w_{k,n}$ and $w_{k,m}$, where n and m are the indices of the weights assigned to the nth and mth features for the vector of weights for the kth class). This imposes constraints on the number of classes and number of features that can be run on a particular hardware graph. For a dataset with M features and K classes, this approach requires $M \times (K-1)$ logical variables, and in general there must be. Due to restrictions on the number of qubits and the limited graph connectivity (see Figure S9), an *embedding*, by which edges in the graph are contracted to give a graph with fewer vertices but a higher degree, is generated (see supplemental experimental procedures for more details). For the D-Wave 2000Q, the largest complete graph that can be embedded[44] consists of 66 logical variables; i.e., $M \times (K-1)$ must be at most 66. For our purposes, we chose $K = 6$ cancer types, which limits the number of features we can use to 13. The largest complete graph that can be embedded onto the DW2X processor at ISI consists of 45 logical variables, so for the binomial datasets we chose a total of 44 features.

SUPPLEMENTAL INFORMATION

Supplemental information can be found online at https://doi.org/10.1016/j.patter.2021.100246.

ACKNOWLEDGMENTS

We would like to thank D-Wave for access to their 2000Q processor in Burnaby, British Columbia, Canada.

AUTHOR CONTRIBUTIONS

T.W.C. and D.A.L. conceived the project. R.Y.L., J.R.G., D.A.L., and T.W.C. conceived the experimental design and wrote the manuscript. R.Y.L., S.G., S.R.B., N.C., and O.E.G. processed, scaled, and analyzed all data.

DECLARATION OF INTERESTS

O.E.G., S.G., J.R.G., N.C., S.R.B., and T.W.C. were employed by Genuity Science during the research project. R.Y.L. was the recipient of a research grant from Genuity Science during the research project. The work of D.A.L. is based upon work (partially) supported by the Office of the Director of National Intelligence (ODNI), Intelligence Advanced Research Projects Activity (IARPA), via a US Army Research Office contract. The views and conclusions contained herein are those of the authors and should not be interpreted as necessarily representing the official policies or endorsements, either expressed or implied, of the ODNI, IARPA, or US government. The US government is authorized to reproduce and distribute reprints for governmental purposes notwithstanding any copyright annotation thereon. The authors declare no other competing interests.

INCLUSION AND DIVERSITY STATEMENT

One or more of the authors of this paper self-identifies as an underrepresented ethnic minority in science. One or more of the authors of this paper received

support from a program designed to increase minority representation in science. While citing references scientifically relevant for this work, we also actively worked to promote gender balance in our reference list. The author list of this paper includes contributors from the location where the research was conducted who participated in the data collection, design, analysis, and/or interpretation of the work.

Received: November 18, 2020
Revised: January 27, 2021
Accepted: March 31, 2021
Published: April 28, 2021

REFERENCES

1. Golub, T.R., Slonim, D.K., Tamayo, P., Huard, C., Gaasenbeek, M., Mesirov, J.P., Coller, H., Loh, M.L., Downing, J.R., Caligiuri, M.A., et al. (1999). Molecular classification of cancer: class discovery and class prediction by gene expression monitoring. Science *286*, 531–537.
2. Nevins, J.R., and Potti, A. (2007). Mining gene expression profiles: expression signatures as cancer phenotypes. Nat. Rev. Genet. *8*, 601–609.
3. Hoadley, K.A., Yau, C., Wolf, D.M., Cherniack, A.D., Tamborero, D., Ng, S., Leiserson, M.D.M., Niu, B., McLellan, M.D., Uzunangelov, V., et al. (2014). Multiplatform analysis of 12 cancer types reveals molecular classification within and across tissues of origin. Cell *158*, 929–944.
4. Hoadley, K.A., Yau, C., Hinoue, T., Wolf, D.M., Lazar, A.J., Drill, E., Shen, R., Taylor, A.M., Cherniack, A.D., Thorsson, V., et al. (2018). Cell-of-origin patterns dominate the molecular classification of 10,000 tumors from 33 types of cancer. Cell *173*, 291–e6.
5. Uhlen, M., Zhang, C., Lee, S., Sjöstedt, E., Fagerberg, L., Bidkhori, G., Benfeitas, R., Arif, M., Liu, Z., Edfors, F., et al. (2017). A pathology atlas of the human cancer transcriptome. Science *357*, eaan2507.
6. Robinson, D.R., Wu, Y.M., Lonigro, R.J., Vats, P., Cobain, E., Everett, J., Cao, X., Rabban, E., Kumar-Sinha, C., Raymond, V., et al. (2017). Integrative clinical genomics of metastatic cancer. Nature *548*, 297–303.
7. Toloşi, L., and Lengauer, T. (2011). Classification with correlated features: unreliability of feature ranking and solutions. Bioinformatics *27*, 1986–1994. https://doi.org/10.1093/bioinformatics/btr300.
8. Lloyd, S., Mohseni, M., and Rebentrost, P. (2014). Quantum principal component analysis. Nat. Phys. *10*, 631–633.
9. Rebentrost, P., Mohseni, M., and Lloyd, S. (2014). Quantum support vector machine for big data classification. Phys. Rev. Lett. *113*, 130503.
10. Biamonte, J., Wittek, P., Pancotti, N., Rebentrost, P., Wiebe, N., and Lloyd, S. (2017). Quantum machine learning. Nature *549*, 195–202.
11. Johnson, M.W., Amin, M.H., Gildert, S., Lanting, T., Hamze, F., Dickson, N., Harris, R., Berkley, A.J., Johansson, J., Bunyk, P., et al. (2011). Quantum annealing with manufactured spins. Nature *473*, 194–198.
12. Lanting, T., Przybysz, A.J., Smirnov, A.Y., Spedalieri, F.M., Amin, M.H., Berkley, A.J., Harris, R., Altomare, F., Boixo, S., Bunyk, P., et al. (2014). Entanglement in a quantum annealing processor. Phys. Rev. X *4*, 021041.
13. Harris, R., Johnson, M.W., Lanting, T., Berkley, A.J., Johnsson, J., Bunyk, P., Tolkacheva, E., Ladizinsky, E., Ladizinsky, N., Oh, T., et al. (2010). Experimental investigation of an eight-qubit unit cell in a superconducting optimization processor. Phys. Rev. B *82*, 024511.
14. Rieffel, E.G., Venturelli, D., O'Gorman, B., Do, M.B., Prystay, E.M., and Smelyanskiy, V.N. (2015). A case study in programming a quantum annealer for hard operational planning problems. Quan. Inf Proc. *14*, 1–36.
15. King, A.D., Carrasquilla, J., Raymond, J., Ozfidan, I., Andriyash, E., Berkley, A., Reis, M., Lanting, T., Harris, R., Altomare, F., et al. (2018). Observation of topological phenomena in a programmable lattice of 1,800 qubits. Nature *560*, 456.
16. Albash, T., and Lidar, D.A. (2018). Demonstration of a scaling advantage for a quantum annealer over simulated annealing. Phys. Rev. X *8*, 031016.
17. Perdomo-Ortiz, A., Dickson, N., Drew-Brook, M., Rose, G., and Aspuru-Guzik, A. (2012). Finding low-energy conformations of lattice protein models by quantum annealing. Sci. Rep. *2*, 571. https://doi.org/10.1038/srep00571.
18. Li, R.Y., Di Felice, R., Rohs, R., and Lidar, D.A. (2018). Quantum annealing versus classical machine learning applied to a simplified computational biology problem. Npj Quan. Inf *4*, 14.
19. Jain, S., Ziauddin, J., Leonchyk, P., Yenkanchi, S., and Geraci, J. (2020). Quantum and classical machine learning for the classification of non-small-cell lung cancer patients. SN Appl. Sci. *2*, 1088. https://doi.org/10.1007/s42452-020-2847-4.
20. Tibshirani, R. (1996). Regression shrinkage and selection via the lasso. J. R. Stat. Soc. Ser. B (Methodological) *58*, 267–288.
21. Hoerl, A.E., and Kennard, R.W. (1970). Ridge regression: biased estimation for nonorthogonal problems. Technometrics *12*, 55–67.
22. Hoerl, A.E., Kannard, R.W., and Baldwin, K.F. (1975). Ridge regression:-some simulations. Commun. Stat. *4*, 105–123.
23. Breiman, L. (2001). Random forests. Machine Learn. *45*, 5–32.
24. Breiman, L., Friedman, J., Stone, C.J., and Olshen, R.A.; Chapman & Hall (1993). Classification and Regression Trees.
25. Hastie, T., Tibshirani, R., Friedman, J.H., and Springer. (2016). The Elements of Statistical Learning: Data Mining, Inference, and Prediction, 2 edn.
26. Ng, A. (2008). Generative Learning Algorithms.
27. Boser, B.E., Guyon, I.M. & Vapnik, V.N. (ACM, 1992).in Proceedings of the Fifth Annual Workshop on Computational Learning Theory. 144-152.
28. Cortes, C., and Vapnik, V. (1995). Support-vector networks. Mach Learn *20*, 273–297.
29. Barahona, F. (1982). On the computational complexity of Ising spin glass models. J. Phys. A Math. Gen. *15*, 3241–3253.
30. Pudenz, K.L., and Lidar, D.A. (2013). Quantum adiabatic machine learning. Quan. Inf. Proc. *12*, 2027–2070.
31. Kirkpatrick, S., Gelatt, C.D., and Vecchi, M.P. (1983). Optimization by simulated annealing. Science *220*, 671–680.
32. Mott, A., Job, J., Vlimant, J.R., Lidar, D., and Spiropulu, M. (2017). Solving a Higgs optimization problem with quantum annealing for machine learning. Nature *550*, 375–379.
33. Willsch, D., Willsch, M., De Raedt, H., and Michielsen, K. (2019). Support vector machines on the D-Wave quantum annealer. arXiv. https://doi.org/10.1016/j.cpc.2019.107006.
34. Robinson, M.D., McCarthy, D.J., and Smyth, G.K. (2010). edgeR: a Bioconductor package for differential expression analysis of digital gene expression data. Bioinformatics *26*, 139–140.
35. Lawson, C.D., Fan, C., Mitin, N., Baker, N.M., George, S.D., Graham, D.M., Perou, C.M., Burridge, K., Der, C.J., and Rossman, K.L. (2016). Rho GTPase transcriptome analysis reveals oncogenic roles for Rho GTPase-activating proteins in basal-like breast cancers. Cancer Res. *76*, 3826–3837.
36. Young, M.D., Wakefield, M.J., Smyth, G.K., and Oshlack, A. (2010). Gene ontology analysis for RNA-seq: accounting for selection bias. Genome Biol. *11*, R14.
37. Winter, D.J. (2017). Rentrez: An R Package for the NCBI eUtils API. Report No. 2167-9843 (PeerJ Preprints).
38. Vuaroqueaux, V., Urban, P., Labuhn, M., Delorenzi, M., Wirapati, P., Benz, C.C., Flury, R., Dieterich, H., Spyratos, F., Eppenberger, U., and Eppenberger-Castori, S. (2007). Low E2F1 transcript levels are a strong determinant of favorable breast cancer outcome. Breast Cancer Res. *9*, R33.
39. Argelaguet, R., Velten, B., Arnol, D., Dietrich, S., Zenz, T., Marioni, J.C., Buettner, F., Huber, W., and Stegle, O. (2018). Multi-Omics Factor Analysis—a framework for unsupervised integration of multi-omics data sets. Mol. Syst. Biol. *14*, e8124. https://doi.org/10.15252/msb.20178124.

40. Cerami, E., Gao, J., Dogrusoz, U., Gross, B.E., Sumer, S.O., Aksoy, B.A., Jacobsen, A., Byrne, C.J., Heuer, M.L., Larsson, E., et al. (2012). The cBio cancer genomics portal: an open platform for exploring multidimensional cancer genomics data. Cancer Discov. *2*, 401–404.

41. Gao, J., Aksoy, B.A., Dogrusoz, U., Dresdner, G., Gross, B., Sumer, S.O., Sun, Y., Jacobsen, A., Sinha, R., Larsson, E., et al. (2013). Integrative analysis of complex cancer genomics and clinical profiles using the cBioPortal. Sci. Signal. *6*, pl1.

42. Isakov, S.V., Zintchenko, I.N., Rønnow, T.F., and Troyer, M. (2015). Optimised simulated annealing for Ising spin glasses. Comput. Phys. Commun. *192*, 265–271.

43. Larochelle, H., Mandel, M., Pascanu, R., and Bengio, Y. (2012). Learning algorithms for the classification restricted Boltzmann machine. J. Machine Learn. Res. *13*, 643–669.

44. Choi, V. (2008). Minor-embedding in adiabatic quantum computation: I. The parameter setting problem. Quan. Inf. Proc. *7*, 193–209.

Patterns, Volume *2*

Supplemental information

Quantum processor-inspired machine learning in the biomedical sciences

Richard Y. Li, Sharvari Gujja, Sweta R. Bajaj, Omar E. Gamel, Nicholas Cilfone, Jeffrey R. Gulcher, Daniel A. Lidar, and Thomas W. Chittenden

Supplemental Items

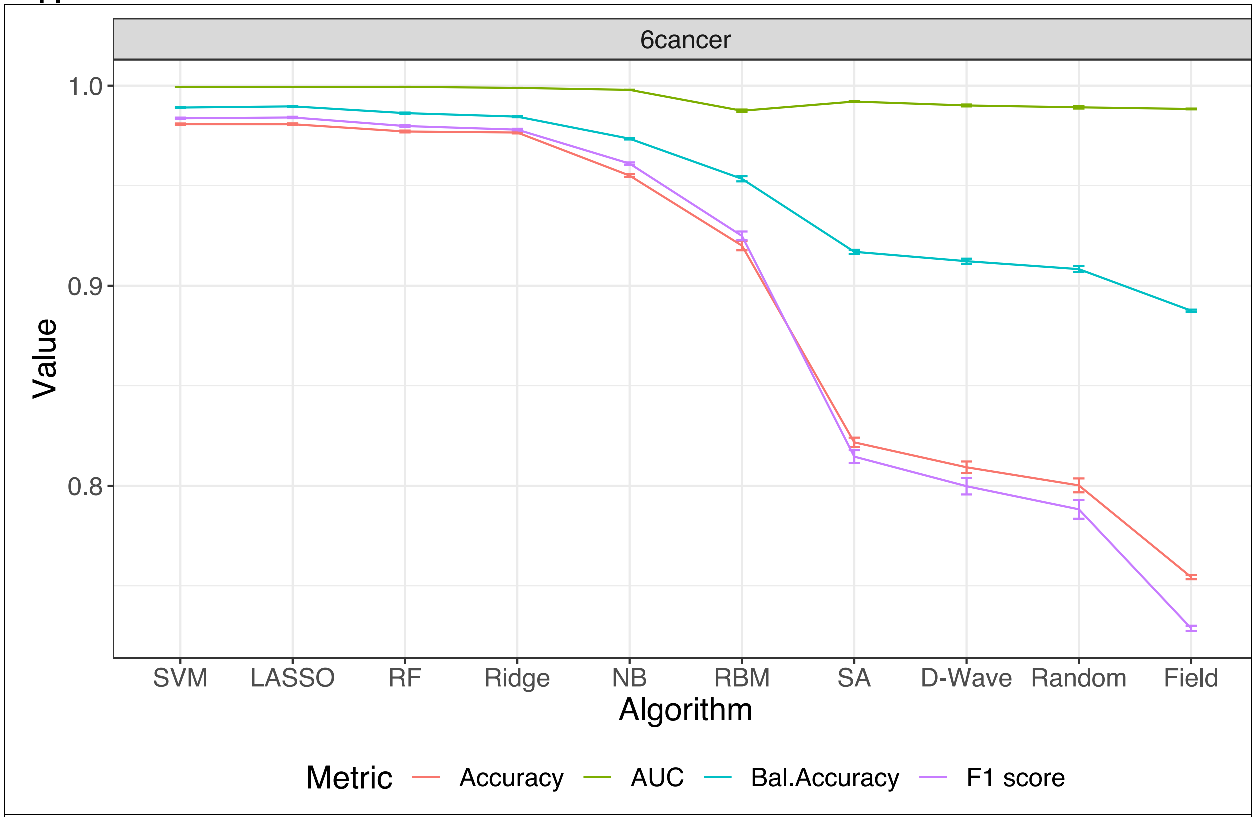

Figure S1: Related to Figure 1. Assessment of algorithm performance for multi-class classification of six TCGA human cancer types: breast, colorectal, lung, kidney, brain, and liver. Classification performance metrics were averaged for the 100 unique test sets for each model (see methods). Performance metrics: Accuracy (red), AUC (green), Balanced Accuracy (blue), F1 score (purple). Data are presented as mean ± SEM.

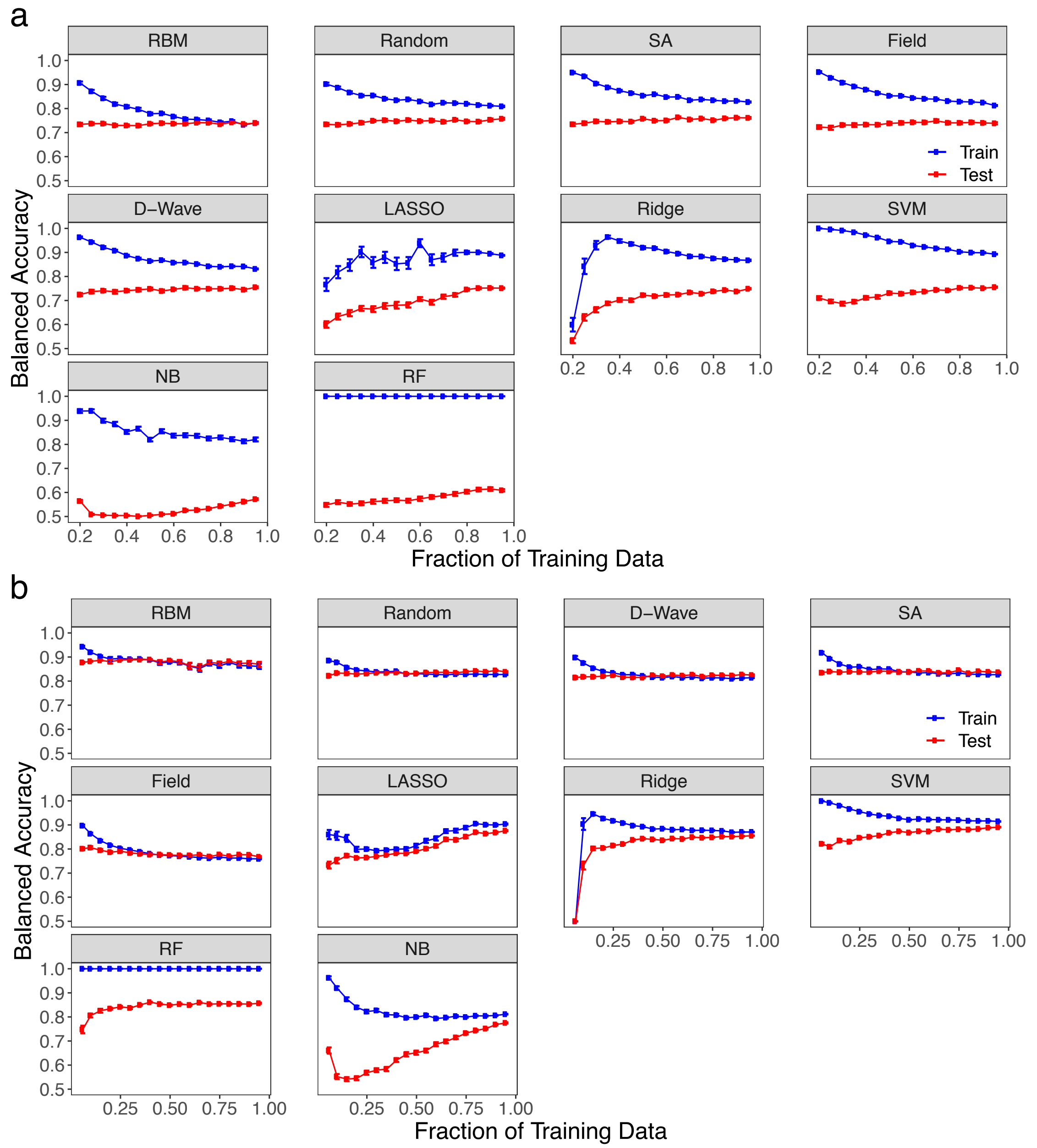

Figure S2: Related to Figure 3. (a) Train and test balanced accuracies for the LumA vs. LumB binomial classification dataset. Order of the panels (left to right, top to bottom) is based on the average difference between performance on the train and the test across the range of data fractions shown for all nine classifiers assessed. (b) Train and test balanced accuracies for the ERpos vs. ERneg binomial classification dataset. Ordering of panels is the same as is used for (a).

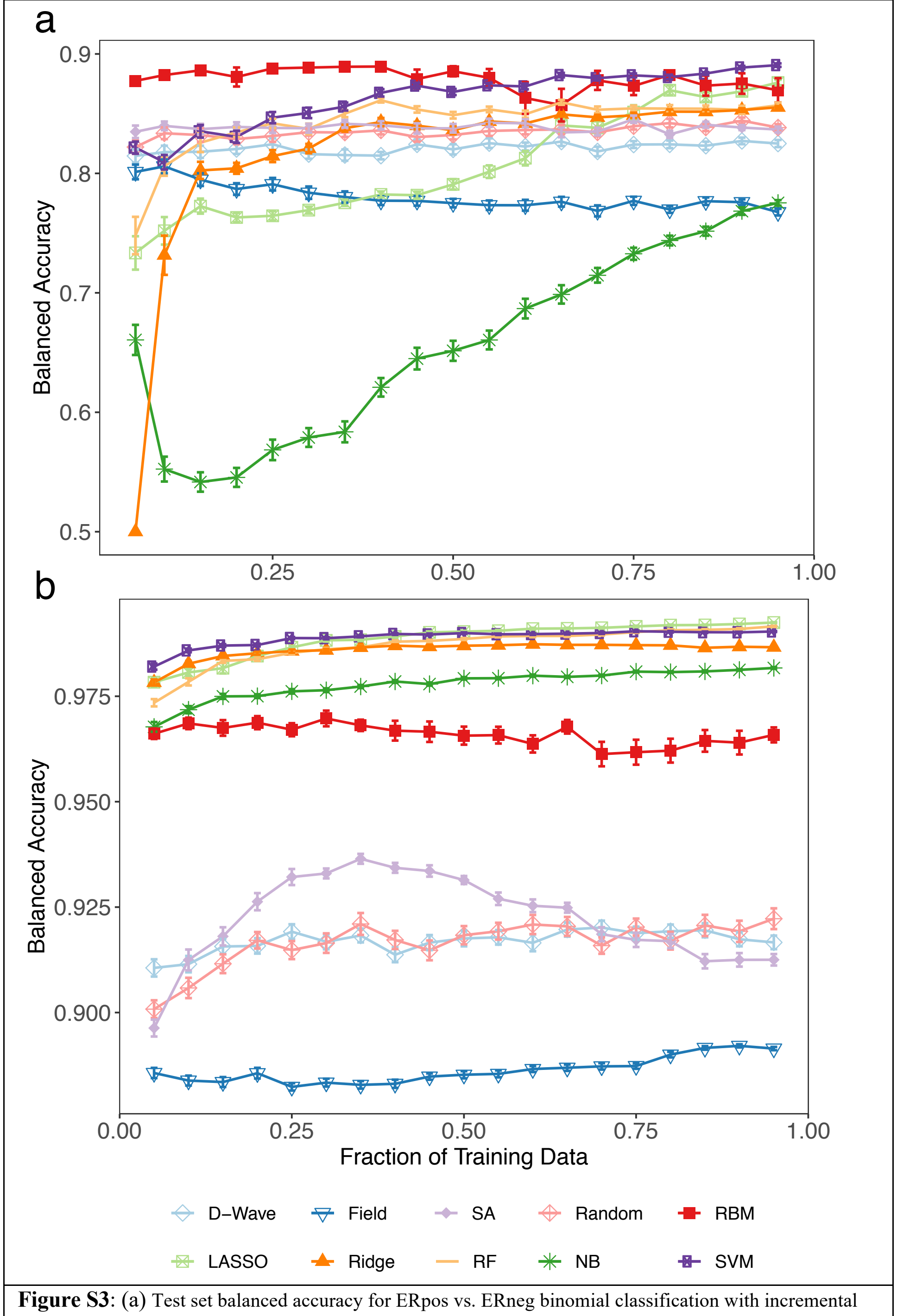

Figure S3: (a) Test set balanced accuracy for ERpos vs. ERneg binomial classification with incremental decreases from 95% to 10% of original training set. (b) Test set balanced accuracy for the six-cancer classification with incremental decreases from 95% to 5% of original training set. The six TCGA human cancer types assessed: breast, colorectal, lung, kidney, brain, and liver. Averaged balanced accuracies were calculated for 50 independent training sets at each designated fraction of original training data. Data are presented as mean ± SEM.

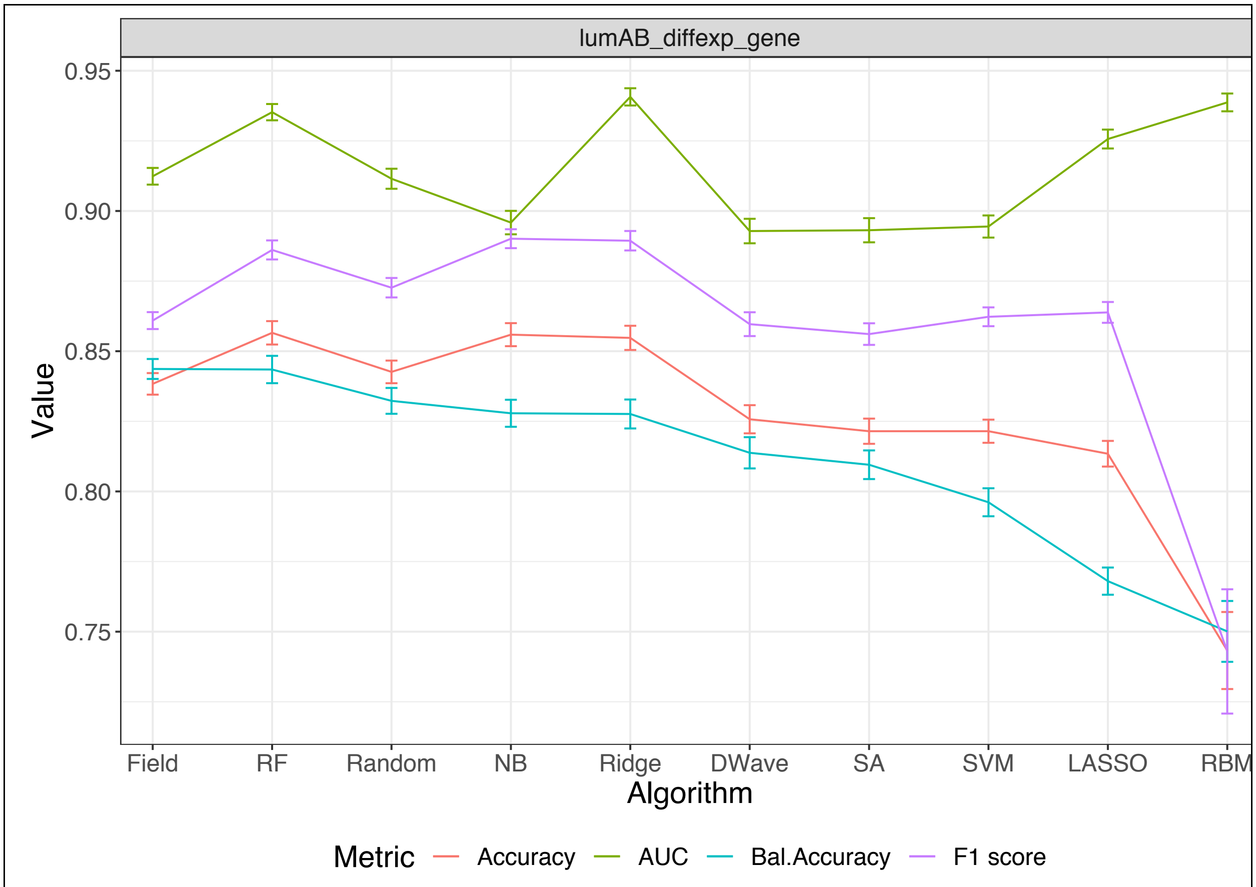

Figure S4: Related to Figure 4. Assessment of algorithm performance for Luminal A versus Luminal B breast cancers based on most differentially expressed genes. Classification performance metrics were averaged for the 100 unique test sets for each model (see methods). Performance metrics: Accuracy (red), AUC (green), Balanced Accuracy (blue), F1 score (purple). Data are presented as mean ± SEM.

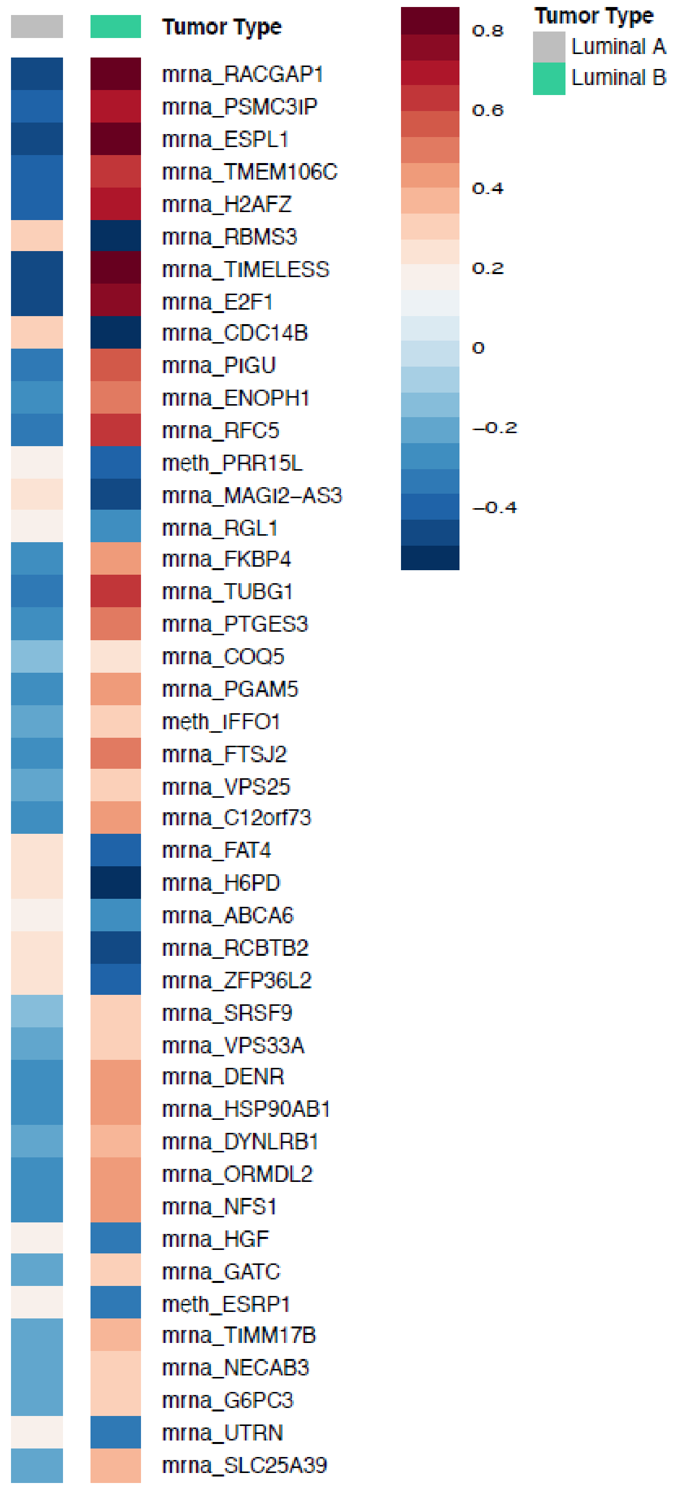

Figure S5: Related to Figure 4. Rank-ordered **h**eatmap of the averaged state for each of the top 44 genes for the LumA vs. LumB comparison.

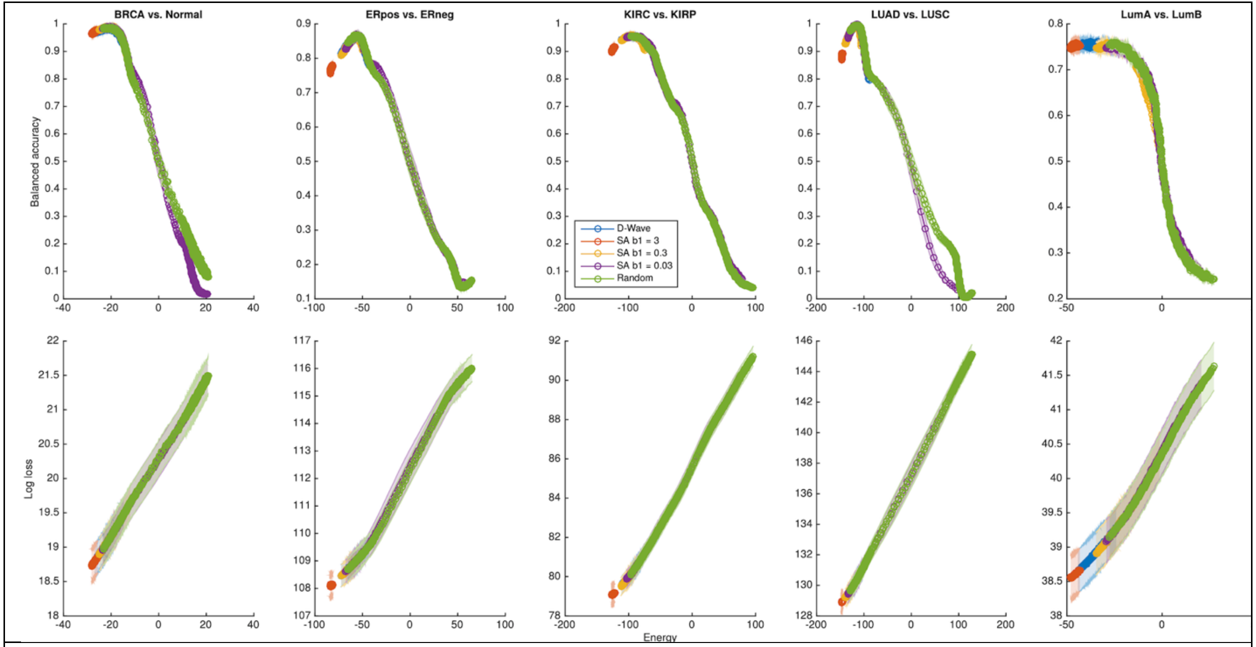

Figure S6: Evaluation of the performance of D-Wave, SA at various final inverse temperatures (b1 in the Legend), and Random, when using a sliding window of energies. Average balanced accuracy (top) and the average log loss (bottom) across the 100 cuts on the test dataset versus the average Ising energy of the post-processed weights. The shaded region represents 2 standard deviations.

Note S1: Related to Figure S6.
As a way to gain some insight into the machine learning performance with respect to Ising energy, we considered the effect of using higher-energy excited-state solutions; i.e., the solutions that have a higher energy according to the Ising Hamiltonian defined in Eq. (19). To do so, we first generated 1000 sets of weights for each method (D-Wave, SA, and Random). The weights were then sorted according to their Ising energy. Next, we used a sliding window of energies; i.e., we applied our post-processing averaging procedure to 20 solutions at a time starting from the 20 set of weights with the lowest Ising energy, then applying the averaging procedure to the 20 set of weights with the next lowest Ising energy and so on. This procedure was repeated for all 100 cuts of the data for each dataset, and the results for the balanced accuracy and negative log-likelihood are presented in Supplemental Figure S6.

The top row of the Supplemental Figure S6 shows a maximum in the balanced accuracy versus the Ising energy for most of the datasets. This maximum indicates the presence of a mismatch between the optimized objective function (the Ising energy) and the performance metric (the balanced accuracy); i.e., some solutions that perform worse in terms of the Ising energy perform better in terms of the balanced accuracy. This is in part due to the nature of the logistic loss, which is somewhat sensitive to outliers. To calculate the balanced accuracy, we must first threshold the predicted probabilities, assigning classes based on whether the probability for that class is greater than 0.5. Further, we note that by tuning the final temperature of SA, we are able to control the weights found to lie within a particular energy range; higher final temperature (smaller b1) shift the energies to the right. Note that while it is possible to bring SA to find higher energy solutions by increasing the final temperature, there is no single parameter we can use to decrease the temperature of the weights found by the random method, other than increasing the number of weights we randomly generate (for D-Wave we can essentially control the temperature by scaling the h's and J's). It seems reasonable to expect that with a growing number of features, Random will become less and less likely to find solutions low enough in energy to be useful; i.e., we may expect the solutions to lie to the right of the maximum. D-Wave and SA are generally to the left of the maximum and we can effectively "raise the temperature" such that they find solutions that are near the maximum.

The bottom row of Supplemental Figure S6 shows the averaged negative log-likelihood across the 100 cuts of the data versus the average Ising energy. Though not always linear, there is a clear correlation between the Ising energy and the log-loss. This indicates that the approximations we used to generate the Ising problem from the log-loss, though perhaps not perfect, are good enough that we see excellent correlations between the two. Had the approximation not been valid, we would expect to see very poor correlation, or no correlation at all between the two.

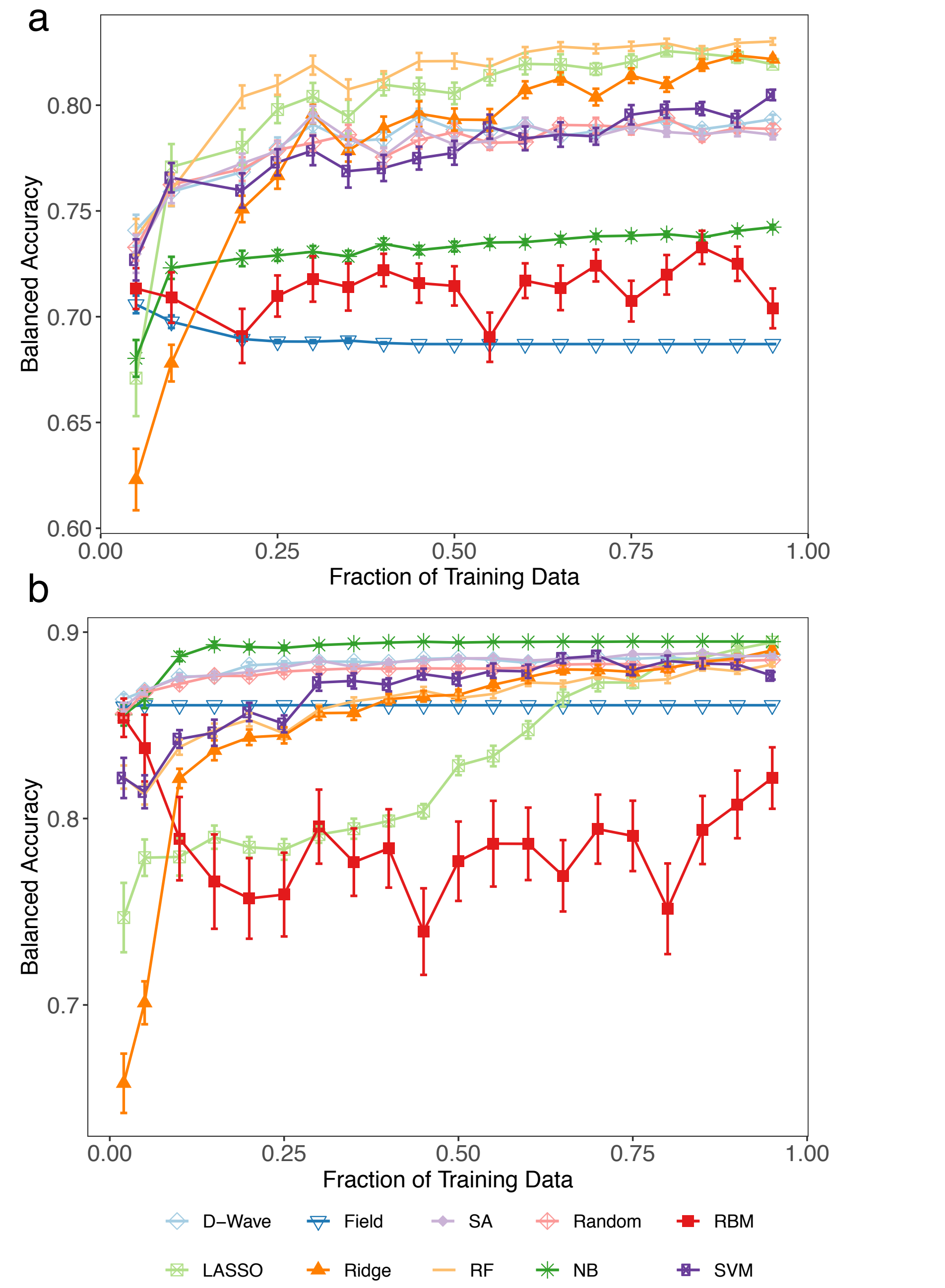

Figure S7: (a) Test set balanced accuracy for LumA vs. LumB binomial classification on top 44 genes from PC1 with incremental decreases from 95% to 5% of original training set. (b) Test set balanced accuracy for ERpos vs. ERneg binomial classification on top 44 genes from PC1 with incremental decreases from 95% to 2% of original training set. Averaged balanced accuracies were calculated for 50 independent training sets at each designated fraction of original training data. Data are presented as mean ± SEM.

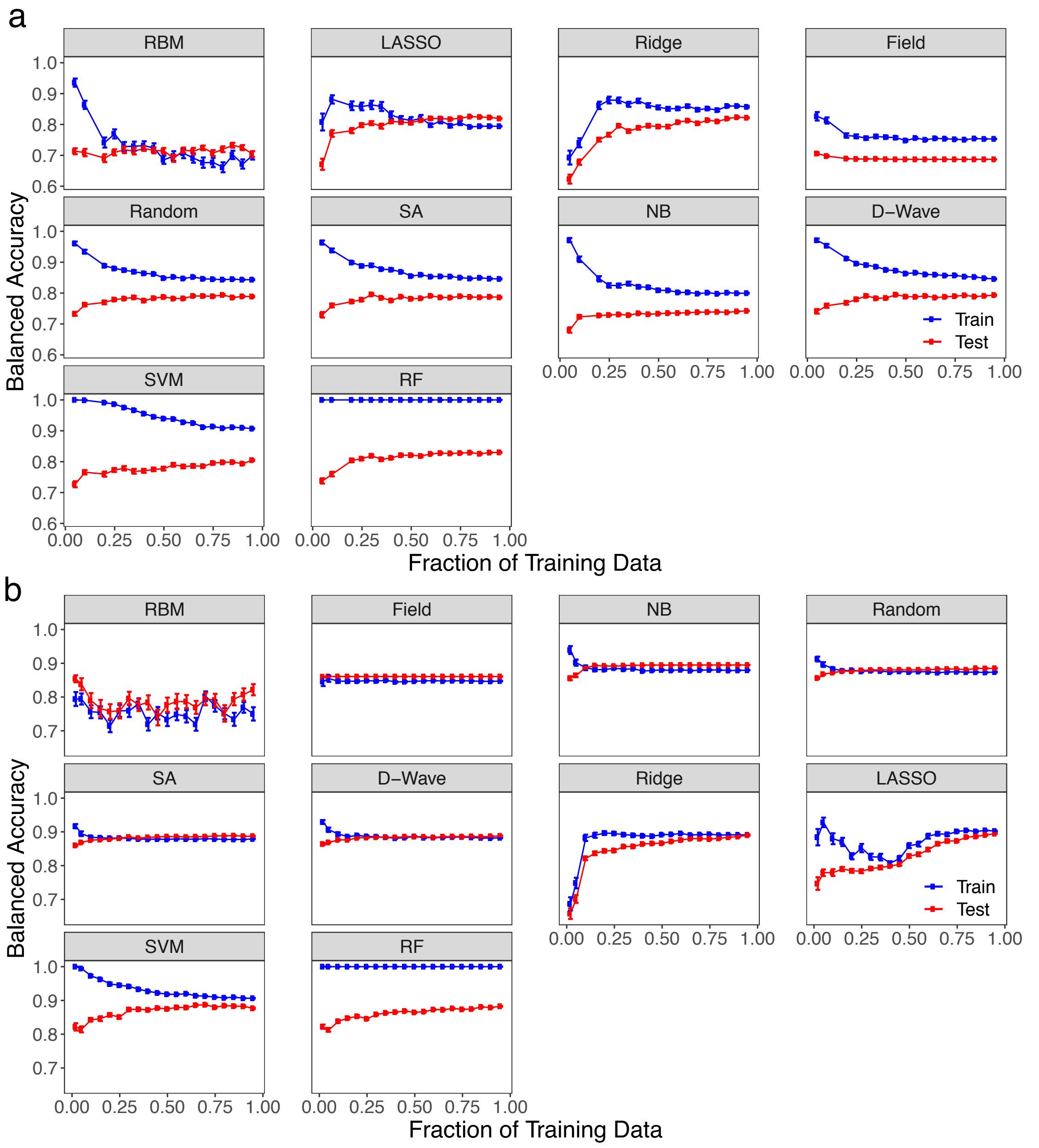

Figure S8: (a) Train and test balanced accuracies for the LumA vs. LumB binomial classification dataset with genes from PC1. Order of the panels (left to right, top to bottom) is based on the average difference between performance on the train and the test across the range of data fractions shown for all nine classifiers assessed. (b) Train and test balanced accuracies for the ERpos vs. ERneg binomial classification dataset with genes from PC1. Ordering of panels is the same as is used for (a).

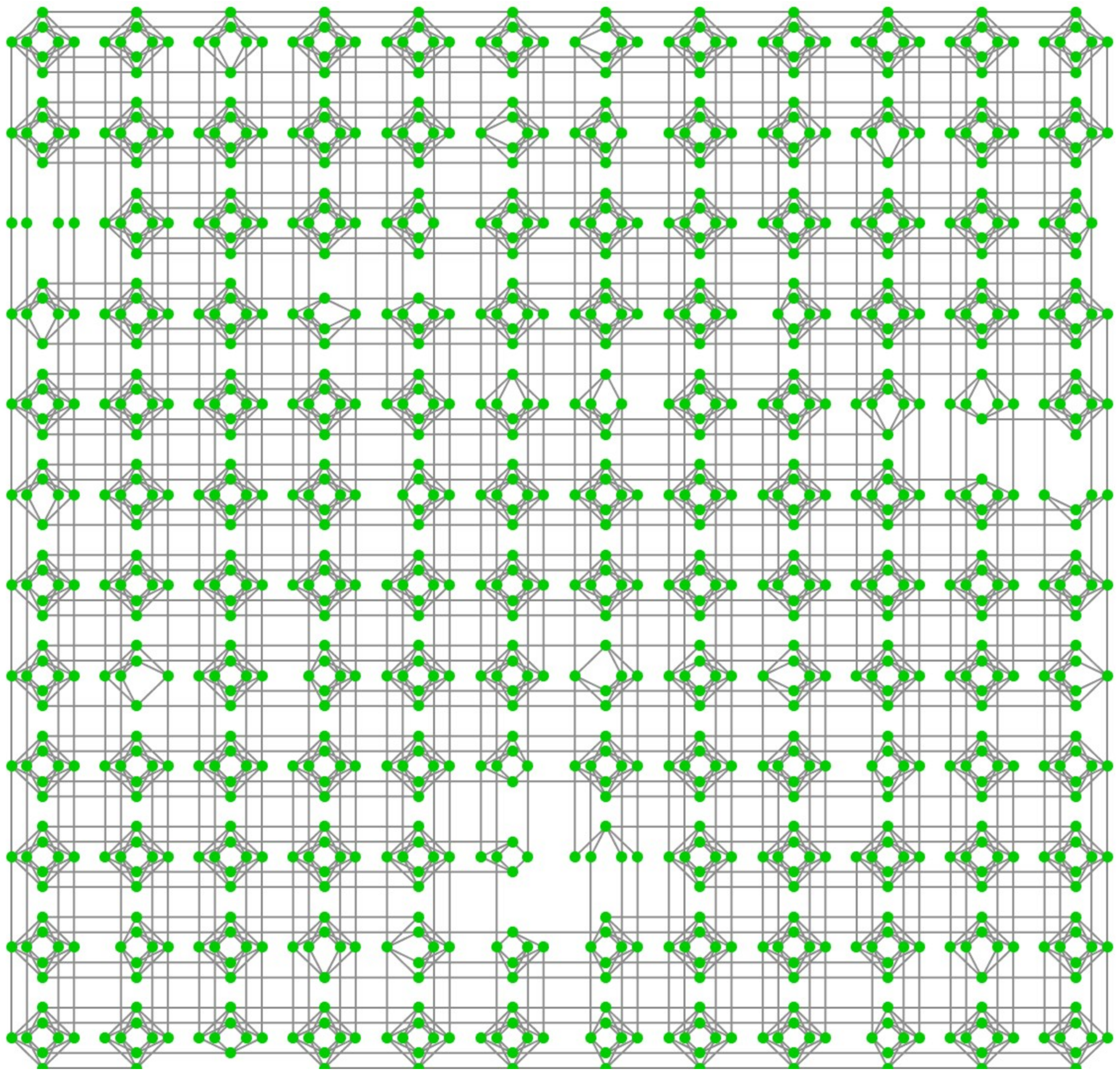

Figure S9: Schematic representation of the "Chimera" hardware graph of the DW2X housed at the Information Sciences Institute at USC, used in this work. Green circles represent active qubits, inactive qubits are omitted, lines represent couplings between qubits. Each qubit can be coupled to a maximum of six other qubits.

Note S2: Related to Figures S10-S12

Because D-Wave, SA, and Random are all probabilistic, returning a distribution of solutions, finding weights with low Ising energy is somewhat dependent on the total number of solutions that are found. We note that the ability of the respective methods in finding low energy solutions differs; as seen in Supplemental Figure S6, with a 1000 solutions, Random does not find solutions that are as low in energy as D-Wave and SA; we might expect the need to randomly generate on the order of 2^{44} (recall that we used 44 PCs for the binomial datasets) solutions in order for Random to find the solutions that match the Ising energy of the solutions returned by D-Wave and SA.

In addition to the total number of solutions, performance is also dependent on the number of solutions we include in our post-processing procedure. Our post-processing procedure is designed to monotonically improve performance on the training datasets, and thus we might expect including more solutions to improve performance. We did not consider using all solutions for several reasons: first, doing so increases the amount of time needed to generate final candidate solutions; second, by including many of

the solutions, we begin to somewhat lose the discrete nature of the weights and therefore some of the robustness associated with them; finally, monotonically improving performance on the training dataset may lead to overfitting, and therefore using a smaller number of solutions is somewhat analogous to an early-stopping regularization scheme.

In the main text and in Supplemental Figure S6, we selected 20 best performing solutions to include in our post-processing procedure. The choice of 1000 and 20 are somewhat arbitrary, so to systematically determine the effect of this number of solutions, we vary the total number of solutions from 1 to 1000 and the number of best performing solutions from 1 to 1000 for each of the five binomial datasets. As before, we used the same 100 cuts of the data described in the main text for each of the datasets. The average test balanced accuracy, average test logistic loss, and the average training negative Ising energy are shown in Supplemental Figures S10, S11, and S12, respectively. For each performance metric (i.e., balanced accuracy, logistic loss, and Ising energy), we chose the final inverse temperature for SA and the J_c for D-Wave (see Section on additional technical details of D-Wave) that gave the best performance of that measure.

Supplemental Figures S10-S12 all show that when using a very small number of total solutions, Random does worse than D-Wave and SA on all metrics. However, with as few as 50 total solutions and 5 best performing solutions, Random performs nearly the same as D-Wave and SA in terms of the balanced accuracy. Further increasing the total number of solutions and the number of solutions used in the post-processing procedure improves the balanced accuracy for D-Wave, SA, and Random. For estrogen receptor positive (ERpos) vs. estrogen receptor negative (ERneg) and lung adenocarcinoma (LUAD) vs. lung squamous cell carcinoma (LUSC), using all 1000 out of 1000 solutions gives the best performance, however for the other datasets, using a smaller number (around 20 or 50) of the total solutions gives nearly equal performance as using all 1000. For Random, using all 1000 solutions for the luminal A (LumA) vs. luminal B (LumB) breast cancers dataset is worse than using 50 solutions, indicating that for this dataset early stopping may help improve performance in terms of the balanced accuracy. Supplemental Figures S11 and S12 show that Random does not find solutions that are as low in Ising energy or with as low of a logistic loss as D-Wave and SA, confirming what was shown in Supplemental Figure S6.

These additional results show that even by exploring only a small subset of the total search space (1000 out of 2^{44} possible solutions), Random is able to give very good machine learning performance. Because the logistic loss is somewhat sensitive to outliers, finding “good” solutions with low (but not the lowest) Ising energy seem to give the best machine learning performance in terms of the balanced accuracy.

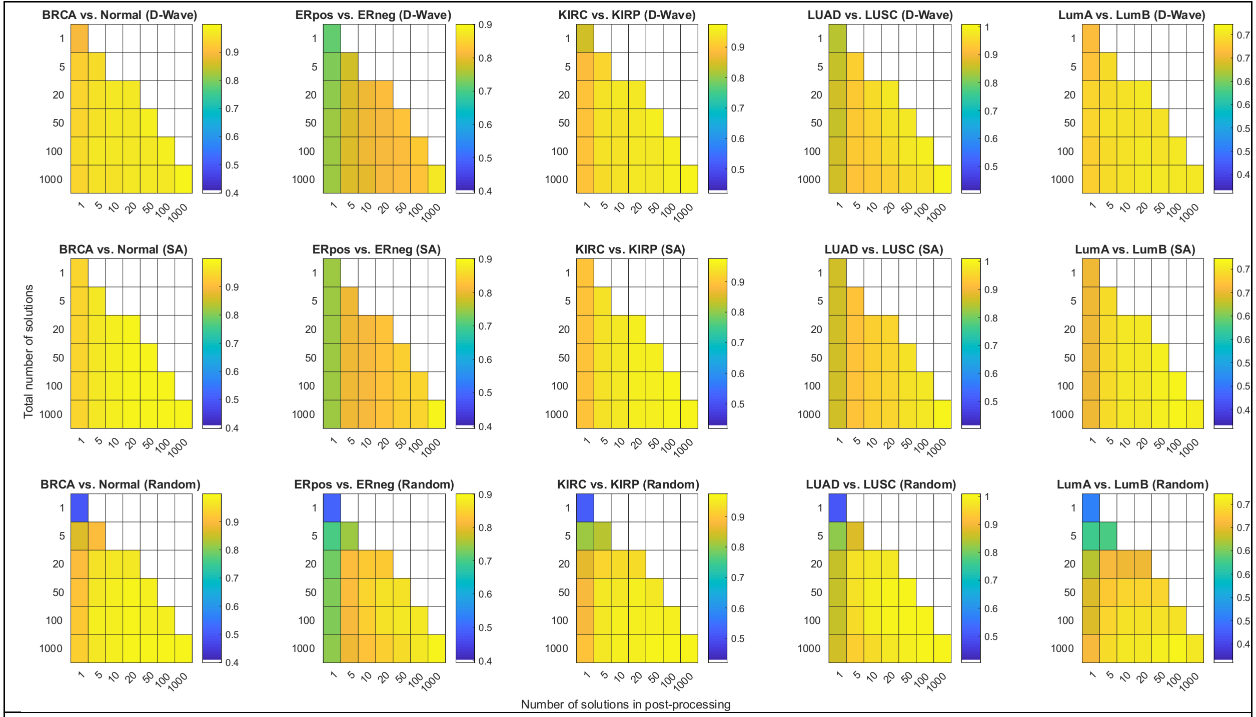

Figure S10: Heatmaps showing the effect of changing the total number of solutions (y-axis) and the number of solutions used in the post-processing procedure (x-axis) on the average balanced accuracy on the test datasets for the five binomial datasets. SA was run with a final inverse temperature of b1 = 0.03 and D-Wave was run with a J_c of 8.0. A higher balanced accuracy indicates better performance.

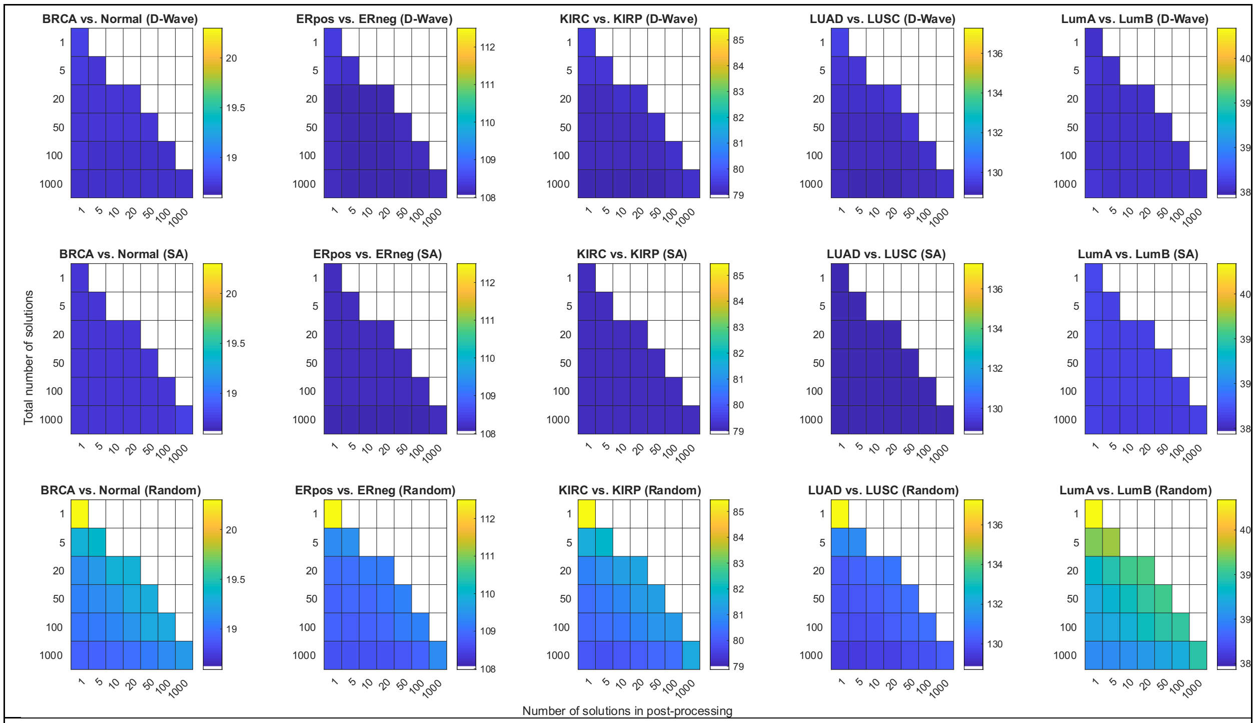

Figure S11: Heatmaps showing the effect of changing the total number of solutions (y-axis) and the number of solutions used in the post-processing procedure (x-axis) on the average logistic loss on the test datasets for the five binomial datasets. SA was run with a final inverse temperature of b1 = 3 and D-Wave was run with a J_c of 1.0. A lower logistic loss indicates better performance.

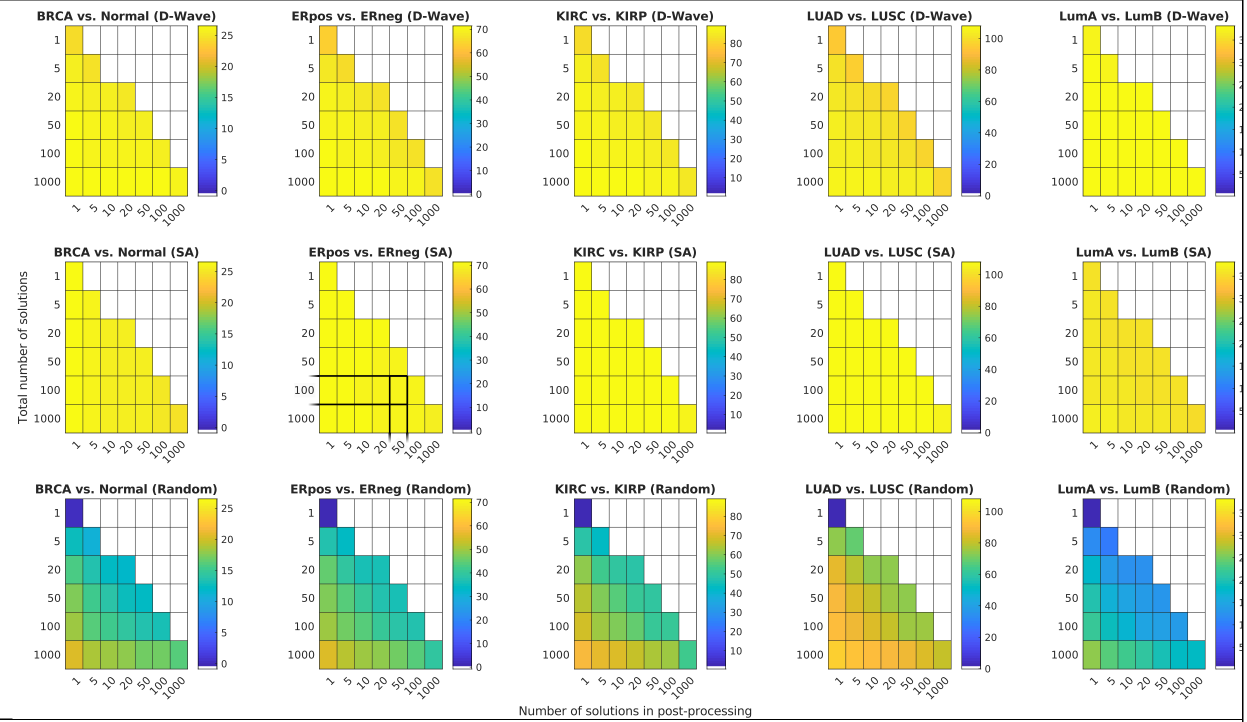

Figure S12: Heatmaps showing the effect of changing the total number of solutions (y-axis) and the number of solutions used in the post-processing procedure (x-axis) on the average negative Ising energy on the training datasets for the five binomial datasets. SA was run with a final inverse temperature of b1 = 3 and D-Wave was run with a J_c of 1.0. A lower Ising energy (higher negative Ising energy) indicates better performance.

Dataset	Training Set	Test Set	Ratio of classes
KIRC vs. KIRP	490	121	53:47
LUAD vs. LUSC	770	192	52:48
BRCA vs. Normal	118	28	50:50
ERpos vs. ERneg	768	191	77:23
LumA vs. LumB	250	61	64:36
Six Cancer Types (brca, coad, kidn, lgg, lihc, lung)	3192	795	25:14:15:13:9:24

Table S1: Datasets with the number of human tumor samples in the training and test sets. Kidney Renal Clear Cell Carcinoma (KIRC) vs. Kidney Renal Papillary Cell Carcinoma (KIRP); Lung Adenocarcinoma (LUAD) vs. Lung Squamous Cell Carcinoma (LUSC); Breast Invasive Carcinoma (BRCA) vs. matched normal tissue (normal); estrogen receptor positive (ERpos) vs. estrogen receptor negative (ERneg) breast cancers; and luminal A (LumA) vs. luminal B (LumB) breast cancers. Six human cancer types: breast, colorectal, lung, kidney, brain, and liver.

Dataset	LASSO	Ridge	SVM	RF	NB	DW	SA	Rand	Field	RBM
BRCA vs Normal	0.981 ± 0.002	0.982 ± 0.002	0.980 ± 0.003	0.989 ± 0.002	0.895 ± 0.006	0.974 ± 0.003	0.981 ± 0.003	0.982 ± 0.002	0.952 ± 0.004	0.981 ± 0.002
ER pos vs ER neg	0.921 ± 0.002	0.919 ± 0.002	0.928 ± 0.002	0.920 ± 0.002	0.875 ± 0.002	0.769 ± 0.003	0.785 ± 0.003	0.803 ± 0.003	0.714 ± 0.004	0.851 ± 0.007
KIRC vs KIRP	0.978 ± 0.001	0.976 ± 0.001	0.979 ± 0.001	0.945 ± 0.002	0.938 ± 0.002	0.946 ± 0.002	0.948 ± 0.002	0.955 ± 0.002	0.894 ± 0.003	0.930 ± 0.002
LUAD vs LUSC	0.9989 ± 0.0002	0.9999 ± 0.0001	1.0000 ± 0.0000	0.9956 ± 0.0003	0.9952 ± 0.0004	0.9482 ± 0.0017	0.9539 ± 0.0017	0.9752 ± 0.0013	0.8619 ± 0.0031	0.9969 ± 0.0003
Lum A vs Lum B	0.788 ± 0.005	0.781 ± 0.005	0.788 ± 0.005	0.722 ± 0.006	0.655 ± 0.006	0.747 ± 0.006	0.750 ± 0.006	0.747 ± 0.006	0.740 ± 0.006	0.721 ± 0.007
6 cancer	0.9807 ± 0.0004	0.9766 ± 0.0004	0.9807 ± 0.0004	0.9771 ± 0.0005	0.9550 ± 0.0007	0.8092 ± 0.0029	0.8217 ± 0.0024	0.8002 ± 0.0035	0.7543 ± 0.0011	0.9200 ± 0.0023

Table S2: Accuracies for five binary classification datasets and the one six-class cancer dataset used in this study. Data reported as mean ± SEM

Dataset	LASSO	Ridge	SVM	RF	NB	DW	SA	Rand	Field	RBM
BRCA vs Normal	0.9998 ± 0.0001	0.9998 ± 0.0001	0.9991 ± 0.0003	0.9977 ± 0.0007	0.9869 ± 0.0016	0.9951 ± 0.0014	0.9982 ± 0.0005	0.9991 ± 0.0003	0.9848 ± 0.0021	0.9956 ± 0.0015
ER pos vs ER neg	0.949 ± 0.002	0.954 ± 0.002	0.953 ± 0.002	0.940 ± 0.002	0.908 ± 0.003	0.894 ± 0.003	0.914 ± 0.003	0.925 ± 0.003	0.835 ± 0.004	0.875 ± 0.003
KIRC vs KIRP	0.9967 ± 0.0004	0.9983 ± 0.0002	0.9974 ± 0.0004	0.9851 ± 0.0011	0.9682 ± 0.0022	0.9787 ± 0.0015	0.9819 ± 0.0013	0.9837 ± 0.0012	0.9558 ± 0.0024	0.9554 ± 0.0026
LUAD vs LUSC	1.0000 ± 0.0000	1.0000 ± 0.0000	1.0000 ± 0.0000	0.9999 ± 0.0000	0.9999 ± 0.0000	0.9883 ± 0.0006	0.9902 ± 0.0006	0.9972 ± 0.0003	0.9371 ± 0.0023	0.9965 ± 0.0004
Lum A vs Lum B	0.856 ± 0.005	0.860 ± 0.005	0.855 ± 0.005	0.816 ± 0.006	0.775 ± 0.007	0.829 ± 0.006	0.838 ± 0.005	0.836 ± 0.006	0.820 ± 0.007	0.783 ± 0.007
6 cancer	0.9994 ± 0.0000	0.9989 ± 0.0001	0.9993 ± 0.0000	0.9994 ± 0.0000	0.9979 ± 0.0000	0.9901 ± 0.0005	0.9920 ± 0.0002	0.9892 ± 0.0006	0.9883 ± 0.0002	0.9875 ± 0.0006

Table S3: AUC for five binary classification datasets and the one six-class cancer dataset used in this study. Data reported as mean ± SEM.

Dataset	LASSO	Ridge	SVM	RF	NB	DW	SA	Rand	Field	RBM
BRCA vs Normal	0.981 ± 0.002	0.982 ± 0.003	0.980 ± 0.002	0.989 ± 0.002	0.885 ± 0.007	0.973 ± 0.003	0.979 ± 0.003	0.981 ± 0.002	0.947 ± 0.005	0.980 ± 0.002
ER pos vs ER neg	0.950 ± 0.001	0.948 ± 0.001	0.954 ± 0.001	0.949 ± 0.001	0.922 ± 0.002	0.830 ± 0.002	0.843 ± 0.003	0.857 ± 0.002	0.786 ± 0.003	0.892 ± 0.009
KIRC vs KIRP	0.979 ± 0.001	0.977 ± 0.001	0.980 ± 0.001	0.948 ± 0.002	0.942 ± 0.002	0.948 ± 0.002	0.950 ± 0.002	0.957 ± 0.002	0.899 ± 0.003	0.935 ± 0.002
LUAD vs LUSC	0.9988 ± 0.0002	0.9999 ± 0.0001	1.0000 ± 0.0000	0.9958 ± 0.0004	0.9954 ± 0.0004	0.9498 ± 0.0017	0.9554 ± 0.0017	0.9760 ± 0.0013	0.8648 ± 0.0032	0.9970 ± 0.0003
Lum A vs Lum B	0.834 ± 0.004	0.836 ± 0.004	0.835 ± 0.005	0.807 ± 0.004	0.776 ± 0.004	0.783 ± 0.006	0.786 ± 0.005	0.783 ± 0.006	0.777 ± 0.006	0.767 ± 0.007
6 cancer	0.9841 ± 0.0003	0.9780 ± 0.0004	0.9837 ± 0.0004	0.9798 ± 0.0004	0.9610 ± 0.0006	0.7998 ± 0.0041	0.8146 ± 0.0032	0.7882 ± 0.0047	0.7287 ± 0.0013	0.9249 ± 0.0021

Table S4: F1 score for five binary classification datasets and the one six-class cancer dataset used in this study. Data reported as mean ± SEM.

			PMC
**Gene Name**	**Functionally Annotated**	**Cancer Hits**	**Breast Cancer Hits**
HGF	1	15967	6356
E2F1	1	13733	5925
GATC	1	1808	212
TIMELESS	1	969	263
HSP90AB1	1	864	299
ESRP1	1	520	322
FAT4	1	437	153
RACGAP1	1	412	221
CDC14B	1	376	118
PGAM5	1	348	89
FKBP4	1	305	127

RFC5	1	303	111
ESPL1	1	260	112
UTRN	1	257	76
ZFP36L2	1	257	65
H2AFZ	1	225	84
VPS25	1	214	36
TUBG1	1	189	81
PTGES3	1	187	50
SRSF9	1	161	59
G6PC3	1	154	37
H6PD	1	149	59
NFS1	1	148	40
RBMS3	1	136	55
RGL1	1	134	47
ABCA6	1	105	47
DENR	1	87	30
VPS33A	1	84	10
PSMC3IP	1	66	28
COQ5	1	61	13
DYNLRB1	1	57	19
PIGU	1	56	24
RCBTB2	1	54	19
TIMM17B	1	51	12
SLC25A39	1	42	11
FTSJ2	1	34	14
MAGI2-AS3	0	25	17
ORMDL2	1	25	10
ENOPH1	1	23	11
NECAB3	1	23	7
TMEM106C	1	23	6
PRR15L	0	21	10
IFFO1	1	17	5
C12orf73	0	0	0

Table S7: Output of semantic search of PubMed Central to assess biological relevance of top 44 genes from PC1 for Luminal A versus Luminal comparison.

Supplemental Experimental Procedures

In this section we provide more details for the methods used in the main text.

Data Sources and Preprocessing

We first describe the sources of the data, then how each data type was preprocessed and scaled, before presenting our dimensionality reduction approaches.

The Cancer Genome Atlas (TCGA) Data

Whole Exome Sequencing, RNA-Seq, miRNA-Seq, DNA Methylation Array, and Genotyping Array data were retrieved from the Genome Data Commons (GDC) data portal (https://portal.gdc.cancer.gov/ - Data Release 4.0) or cBioportal (http://www.cbioportal.org/)[1].

Cancer types with samples having all five data types (messenger-RNA, micro-RNA, copy number variation, single nucleotide polymorphism, and DNA methylation) were chosen for further analysis (Figure 5 and Table S1). The cancer types for the five binomial comparisons were kidney renal clear cell carcinoma (KIRC) vs. kidney renal papillary cell carcinoma (KIRP); lung adenocarcinoma (LUAD) vs. lung squamous cell carcinoma (LUSC); breast invasive carcinoma (BRCA) vs. matched normal breast tissue (normal); estrogen receptor positive (ERpos) vs. estrogen receptor negative (ERneg) breast cancers; and luminal A (LumA) vs. luminal B (LumB) breast cancers. We used human brain, breast, kidney, lung, liver, and colorectal cancer types for the six-cancer multiclass classification. The cancer types which were merged into a single cancer type due to their similarity are colon adenocarcinoma (COAD) and rectum adenocarcinoma (READ); kidney renal clear cell carcinoma (KIRC) and kidney renal papillary cell carcinoma (KIRP); lung adenocarcinoma (LUAD) and lung squamous cell carcinoma (LUSC).

Whole Exome Sequencing (STV)

We retrieved GDC harmonized level 2 Variant Call Format (VCF) files annotated by VarScan2[2] and MuTect[3] GDC somatic annotation workflows (with the Variant Effect Predictor (VEP) v84[4]. VCF files were converted to Genomically Ordered Relational (GOR) database file format[5]. DeepCODE scores (described below) were calculated for all variants. Variants were initially filtered by VCF 'Filter' equal to 'Pass', VarScan2 p-value less than or equal to 0.05, and 'Somatic' status and subsequently filtered by VEP annotation 'impact' and deepCODE score and kept if the following conditions were met: (1) 'HIGH' VEP impact, (2) a deepCODE score greater than 0.51 and a 'MODERATE' VEP impact, or (3) 'MODERATE' VEP impact in the absence of a deepCODE score. Call copies for each variant was mapped to its given gene and the counts of all variants ascribed to a given gene were added together into a single count value (referred to as a somatic tumor variant, STV, herein). Variants for the matched breast cancer tumor and normal samples were detected from aligned reads of GDC harmonized level 1 BAM files using the Genome Analysis Toolkit (GATK) Haplotypecaller[6-8]. Joint genotyping was performed on gVCF files using GATK GenotypeGVCFs and hg38 as reference. VEP v85 annotations were obtained by mapping to chromosome position. Variant filtering and call-copy collapsing methods were carried out in the same manner as described above.

RNA-Seq (mRNA)

We retrieved GDC harmonized level 3 mRNA quantification data as un-normalized raw read counts from HT-Seq[9]. Raw mapping counts were combined into a count matrix with genes as rows and samples as columns and normalized using the trimmed mean of M-values (TMM)[10] method from the edgeR[11] R package. Lowly expressed genes were filtered out by requiring read counts to be greater than 1 per million reads for more than 10% of samples. We assessed possible batch effects in the normalized count data using batch information extracted from TCGA barcodes (i.e. the sample plate number) with the ComBat[12] function from the sva[13] R package. There were no detectible batch effects as assessed by Multi-Dimensional Scaling (MDS) either before or after batch correction.

miRNA-Seq (miRNA)

We retrieved GDC harmonized level 3 miRNA quantification data as raw read counts from the BCGSC miRNA profiling pipeline. We filtered miRNAs by retaining only experimentally validated gene targets from the miRBase reference (http://www.mirbase.org/). Raw mapping counts were combined into a count matrix with genes as rows and samples as columns and normalized using the trimmed mean of M-values TMM)[10] method from the edgeR[11] R package. Lowly expressed genes were filtered out by requiring read counts to be greater than 1 per million reads for more than 1% of samples.

Genotyping Arrays (CNV)

We retrieved GISTIC2 processed copy number variation (CNV) data from cBioportal[1,14,15]. GISTIC2 assigns an integer value for each gene ranging from -2 to +2, representing a deep loss, shallow loss, diploid, low-level gain, and high-level amplification accordingly. CNV data was compiled into a matrix with samples as rows and genes as columns and all NA values were removed. For the matched breast cancer tumor and normal samples, we retrieved GDC harmonized level-3 copy number data from Affymetrix SNP 6.0 arrays. The segment means were converted to linear copy numbers using Eq. 1 and mapped to gene symbols using ENSEMBL GRCh38 as reference[16].

$$LinearCopyNumber = 2 \times (2^{SegmentMean}) \quad (1)$$

CNV segments with less than 5 probes and probe sets with frequent germline copy-number variation (using SNP6 array probe set file as reference) were discarded.

DNA Methylation Arrays (Methylation)

We retrieved GDC harmonized level 3 beta values derived from Illumina Infinium Human Methylation27 (HM27) and HumanMethylation450 (HM450) arrays. Probes were filtered based on the following criteria: (1) was present on both platforms, (2) was mapped to genes or their promoters, (3) was not present on chromosome X, Y, or MT, and (4) did not contain all NA values. We replaced remaining NA and zero beta values with the minimum beta value across all probes and all samples in each batch (defined by the samples TCGA plate barcode) as described in the REMP R package[17]. Beta values of 1 were replaced with the maximum beta value less than 1 across all probes and all samples in each batch. We converted beta values into M values using Eq. 2.

$$M(\beta) = \log_2 \left(\frac{\beta}{1-\beta} \right) \quad (2)$$

We corrected for batch effects within each cancer type using batch information extracted from TCGA barcodes (i.e. the sample plate number) with the ComBat[12] function from the sva[13] R package. We collapsed multiple probes mapped to the same gene by selecting the probe with the maximum standard deviation across all samples.

Genomic Data Integration

We concatenated the processed data from each of five genomic data types (mRNA, miRNA, STV, CNV, and Methylation) into a single data matrix, with samples represented in rows and genes (tagged by data type) as columns. For each comparison, samples were randomly split into 100 cuts of training (80%) and testing (20%) datasets stratified by cancer type and/or molecular subtype.

Normalization

For every cut of training dataset, each feature was scaled to zero mean and unit variance (z-score) and the mean and variance from the training datasets were used to standardize the test datasets.

Dimensionality Reduction

Principal Component Analysis (PCA)

Dimensionality reduction was performed using principal component analysis on each cut of the training data retaining the top 44 principal components as features for the binomial comparisons, and 13 principal components as features for the six-cancer multiclass classification. Each cut of the PC-level data was normalized as mentioned above. In order to avoid data leakage, PCA was performed on the training data, and the test data was then projected onto the PCs defined by the training data. These 100 data matrices with 80% training and 20% testing at the PC level were used for downstream modeling (see Figure 1 for an overview of the classification strategy, and Figure 2 for performance on the binomial comparisons and Figure S1 for performance on the six-cancer multiclass comparison).

EdgeR Analysis

To confirm gene-level classification performance, a simple dual dimensionality reduction and differential analysis approach was performed on a cut of TMM[10] normalized training data of the LumA vs. LumB comparison with edgeR[11], a robust negative binomial model, to determine differentially expressed mRNAs. To account for false discovery, the Benjamini-Hochberg procedure was used to adjust ordinal p-values. The top 44 differentially expressed mRNAs were then used for gene-level classification on the same 100 cuts of the data, though of course with mRNA features, instead of PCA features (see Figure S4).

Decreasing the Amount of Training Data

Based on previous results that showed a benefit for annealing approaches over classical machine learning approaches with smaller amounts of data[18-20], we incrementally decreased the amount of training data for the luminal A (LumA) vs. luminal B (LumB), and ER positive (ERpos) vs. ER negative (ERneg) binomial comparisons, as well as the six-cancer multiclass dataset. To do this, we selected one of the original training cuts that consisted of 80% of the entire dataset. From this one cut, we selected fractions of the data in increments of 5%, making sure that we had at least as many samples as PCs. For example, since the luminal A (LumA) vs. luminal B (LumB) breast cancers dataset had 250 samples and 44 PCs, we selected fractions of data in increments of 5% starting with 20% of the data (20% of 250 samples is 50 samples, which is greater than the number of PCs) up to 95% of the original training cut. In order to collect statistics, for percentage p of the training data we sampled p% of the original training data with all the gene-level features 50 times. For each of these cuts, we reperformed PCA to identify the top 44 PCs of the reduced sub-training set. We trained all classical and Ising models on the same sub-training sets and evaluated performance on the original test set consisting of 20% of the data. The results are presented in Figure 3. Formally, let the original training data set on the gene-level data before PCA be denoted by D_1^{train} which is 80% of the entire dataset (in the main text we repeated this step 100 times, i.e., we had a set of training instances $\{D_i^{train}\}_{i=1}^{100}$), and let the corresponding test data set be noted by D_1^{test}. Here, we selected one of the training cuts and generated 50 “sub”-training data sets for each p, which we denote by $\widetilde{D}_{p,j}^{train}$, where (for LumA vs. LumB) $p \in \{20, 25, \ldots, 95\}$ and $j \in \{1, \ldots, 50\}$. Each $\widetilde{D}_{p,j}^{train}$ is obtained by performing PCA on a randomly selected p% of the 250 samples in D_1^{train} with PCA performed on the original 79,000+ gene-level features. For ERpos vs. ERneg, we set the smallest $p = 10\%$ (Figure S3a), and for the six-cancer class the smallest $p = 5\%$ (Figure S3b).

We also performed the same analysis on the top 44 genes based on their loading for PC1 for the LumA vs. LumB and ERpos vs. ERneg comparisons. Since we are no longer restricted to have as many as features as we were with PCA, we decreased the amount of training data to $p = 5\%$ for LumA vs. LumB and $p = 2\%$ for ERpos vs. ERneg.

Finally, to assess the degree of overfitting, we plotted the performance on the train data and the test data across all training fractions for all 9 classifiers (Figure S2 for the PCA-level features and Figure S8 for the gene-level features), with the difference between train and test being a measure of overfitting. We decided to plot both train and test, rather than just the difference, so that the absolute level of performance between algorithms would be readily apparent; for some fractions of training data, the difference between train and test on a conventional machine learning algorithm was very small, but final training balanced accuracy was around 50% (e.g., Ridge at 20% of the training data in Figure S2a).

Machine Learning

We used five machine learning approaches as conventional classification models. The relevant hyper-parameters for each method are mentioned in their respective sections. Hyper-parameters were chosen by using 10-fold cross-validation on the training data, with performance evaluated on the held-out test data.

Least Absolute Shrinkage and Selection Operator (LASSO), and Ridge Regression

LASSO[21] is an L_1-penalized linear regression model defined as:

$$\hat{\beta}(\lambda) = \min_{\beta} \left[-\log \left[L(y; \beta) \right] + \lambda ||\beta||_1 \right] \quad (3)$$

Ridge[22,23] is an L_2-penalized linear regression model defined as:

$$\hat{\beta}(\lambda) = \min_{\beta}\left[-\log\left[L(y;\beta)\right] + \lambda||\beta||_2^2\right] \tag{4}$$

where

$$L = \frac{1}{N}\sum_{i=1}^{N}\left(y_i - \beta_0 - \mathbf{x}_i \cdot \beta\right)^2$$

In both cases $\lambda > 0$ is the regularization parameter that controls model complexity, β are the regression coefficients, β_0 is the intercept term, y are the class labels, x_i is the ith training sample, and the goal of the training procedure is to determine $\hat{\beta}$, the optimal regression coefficients that minimize the quantities defined in Eqs. (3) and (4). The predicted label is given by $\hat{y} = \beta_0 + x_i \cdot \beta$, with some threshold introduced to binarize the label for classification problems. In LASSO, the constraint placed on the norm of β (the strength of which is given by λ) causes coefficients of uninformative features to shrink to zero. This leads to a simpler model that contains only a few non-zero β coefficients. We used the 'glmnet' function from the caret[24] R package to train all LASSO and Ridge models. For Ridge, λ plays a similar role in determining model complexity, except that coefficients for uninformative features do not necessarily shrink to zero.

For both LASSO and Ridge, we chose to implement the function over a custom tuning grid of 1000 values ranging from $\lambda = 0$ to $\lambda = 100$. λ was chosen via 10-fold cross-validation as the value that gave the minimum mean cross-validated error.

Support Vector Machines (SVMs)

Support vector machines (SVMs)[25,26] are a set of supervised learning models used for classification and regression analysis. The primal form of the optimization problem is:

$$\min_{\mathbf{w},b,a} L_p = \frac{1}{2}||\mathbf{w}||_2^2 = \sum_{i=1}^{N} a_i y_i(\mathbf{x}_i \cdot \mathbf{w} + b) + \sum_{i=1}^{N} a_i \tag{5}$$

where L_p is the loss function in its primal form (p for primal), w are the weights to be determined in the optimization, $\boldsymbol{x_i}$ is the ith training sample, y_i is the label of the ith training sample, $a_i \geq 0$ are Lagrange multipliers, N is the number of training points, and b is the intercept term. Labels are predicted by thresholding $x_i \cdot w + b$.

The optimization problem in its dual form is defined as:

$$\max_{a} L_D(a) = \sum_{i=1}^{N} a_i - \frac{1}{2}\sum_{i,j=1}^{N} a_i a_j y_i y_j K\left(\mathbf{x}_i, \mathbf{x}_j\right) \tag{6}$$

where L_D is the Lagrangian dual of the primal problem, a_i are the Lagrange multipliers, y_i and x_i are the ith label and training sample, respectively, $K(\cdot,\cdot)$ is the kernel function. Maximization takes place subject to the constraints $\sum_i a_i y_i = 0$ and $a_i \geq C \geq 0, \forall i$. Here C is a hyper-parameter that controls the degree of misclassification of the model for nonlinear classifiers. The optimal value of w and b can found in terms of the a_i's, and the label of a new data point x can be found by thresholding the output $\sum_i a_i y_i K(x_i, x) + b$.

In most cases, many of the a_i's are zero and evaluating predictions can be faster using the dual form. We used the support vector machines with linear kernel ('svmLinear2') (i.e., $K(x_i, x_j) = x_i \cdot x_j$ the inner product of x_i and x_j) function from the caret[24] R package to train all SVM models. A 10-fold cross-validation was used to tune parameters resulting in best cross-validation accuracy for training the model, using the default tuning grid in *caret*.

Random Forest

Random Forest[27,28] is an ensemble learning method for classification and regression which builds a set (or forest) of decision trees. In random forest, n samples are chosen (typically two-thirds of all the training data) with replacement from the training data m times, giving m different decision trees. Each tree is

grown by considering 'mtry' of the total features, and the tree is split depending on which features gives the smallest Gini impurity. In the event of multiple training samples in a terminal node of a particular tree, the predicted label is given by the mode of all the training samples in a terminal node. The final prediction for a new sample x is determined by taking the majority vote over all the trees in the forest. We used the 'rf' function from the caret[24] R package to train all Random Forest models. A 10-fold cross-validation was used to tune parameters for training the model. A tune grid with 44 values from 1 to 44 for 'mtry', the number of random variables considered for a split each iteration during the construction of each tree, was used for the tuning model.

Naïve Bayes

Naïve Bayes[29,30] is a conditional probabilistic classifier based on applying Bayes' theorem which relies on strong independence assumptions, as defined by Eqs. 7 and 8:

$$P(y_i|\mathbf{x}_i) = \frac{P(y_i)P(\mathbf{x}_i|y_i)}{P(\mathbf{x}_i)} \tag{7}$$

$$P(y_i|x_{i,1}, \ldots, x_{i,m}) = P(y_i)\prod_{k=1}^{m} P(x_{i,k}|y_i) \tag{8}$$

where $x_{i,k}$ is the kth feature of the ith training sample x_i, y_i is the given class label, and m is the number of features. We used the 'nb' function from the caret[24] R package to train all Naïve Bayes models.

Computational Frameworks and Resources

Data pre-processing and machine learning models were carried out using R (>= 3.4.4) or Python (3.6.8). Plots were generated using ggplot2 in R.

Methods for Gene-Level Analysis of LumA vs. LumB

Differential Gene Expression Analysis

To generate Figure 4, we performed differential expression analysis for 41 mRNA genes from top 44 most informative PC1 genes in LumA vs. LumB breast cancer comparison. The edgeR[11] package was used to determine differentially expressed mRNAs. The Benjamini-Hochberg was used to control for false discovery of 5%. Of the 41 mRNA genes, we found 40 genes were significantly differentially expressed with an FDR ≤ 0.05. We found 30 genes had higher expression in Luminal B and 11 genes had higher expression in Luminal A samples based on edgeR analysis. Moreover, there were a total 7,871/18,059 (44%) differently expressed mRNA genes for the Luminal A vs. Luminal B breast cancer comparison. Of these 7,871 genes, 4,345 (55%) were up regulated in Luminal B compared to 3,526 (45%) in Luminal A. To confirm similar performance on PCA derived gene-level classification results, a second edgeR analysis, independent of PCA dimensionality reduction, was also performed on the LumA vs. LumB comparison as described above.

Functional Enrichment Analysis (GOseq)

Functional enrichment analysis of the top 44 most informative genes by PC loading of PC1 from the training set of luminal A (LumA) vs. luminal B (LumB) breast cancers comparison was carried out with GOseq[31] analysis in an unrestricted manner. Briefly, GOseq analysis was performed on the top 44 gene list to identify enriched gene ontology (GO) terms allowing unannotated genes in the analysis. Select GOseq terms ordered by p-value are shown in Figure 4d. A complete list of functionally enriched GO terms is presented in Table S6.

Semantic Search Engine

The '*entrez search*' function from the R package '*rentrez*'[32] was used to query the number of full-text publications for each of the top 44 most informative genes in Luminal A vs. Luminal B breast cancer comparison from the PubMed Central (PMC) database. Briefly, the R package '*rentrez*' provides an

interface to the NCBI's '*EUtils*' API to search databases like GenBank [https://www.ncbi.nlm.nih.gov/genbank/] and PubMed [https://www.ncbi.nlm.nih.gov/pubmed/] for relationships between genes of interest and query terms, and to process the results from the retrieved hits. The search term was defined by combining the gene symbol and "*cancer*" or "*breast cancer*" fields, along with the Medical Subject Headings (MeSH) vocabulary terms as synonyms to expand each NLP search using Boolean operators *AND/OR* (see Table S7). Network diagrams were constructed using Circos scripts (http://circos.ca/). The red and blue outer bands represent 'mRNA' and 'methylation' datatypes, respectively. The inner blue band are genes with known functional annotation at the time of analysis. The purple colored ring indicates the total number of publications where each gene and cancer are both mentioned. This band is colored with five bins where white is the lowest and dark purple the highest. For example, there are many publications that mentions both "E2F1" and "cancer", and very few with "C12orf73" and "cancer". The thickness and color of the Circos plot ribbons indicate number of published full-text articles linking each gene to the cancer or breast cancer.

Hierarchical Clustering
We applied a "custom ward" linkage criteria in the hierarchical cluster[33] analysis of top 44 most informative genes, by PC loading, of PC1 from the training set of the luminal A (LumA) vs. luminal B (LumB) breast cancers comparison (Figure 4b). The genes are represented as rows, and samples as columns. The algorithm used an exact minimization procedure.

Quantum Annealing
Quantum annealing may be considered a special case of adiabatic quantum computation[34]. The adiabatic theorem of quantum mechanics, which underlies quantum annealing, implies that a physical system will remain in the ground state if a given perturbation acts slowly enough and if there is a gap between the ground state and the rest of the system's energy spectrum[35]. To use the adiabatic theorem to solve optimization problems, we specify an initial Hamiltonian, H_B, whose ground state is easy to find (typically a transverse field), and a problem Hamiltonian, H_P, that does not commute with H_B and whose ground state encodes the solution to the problem we are seeking to optimize[36]. We then interpolate from H_B to H_P by defining the total Hamiltonian $H(s) = A(s)H_B + B(s)H_P$, where s is the parameterized time ($0 \leq s = t/t_f \leq 1$, t is time, and t_f is the total annealing time), $A(s)$ and $B(s)$ are, respectively, decreasing and increasing smoothly and monotonically. The adiabatic theorem ensures that the ground state of the system at $s = 1$ will give the desired solution to the problem, provided the interpolation is sufficiently slow, i.e., t_f is large compared to the timescale set by the inverse of the smallest ground state gap of $H(s)$ and by $\frac{dH(s)}{ds}$ [37]. In quantum annealing, rather than run the computation a single time slowly enough such that the adiabatic theorem is obeyed, we allow the possibility of running the computation multiple times at a shorter annealing time, such that the overall computational time is minimized[38]. In addition, when quantum annealing is implemented in a physical device, temperature and other noise effects play an important role; thermal excitation and relaxation cannot be neglected and affect performance[39-41].

Additional technical details regarding the D-Wave quantum annealers
D-Wave processors currently employ a "Chimera" architecture with a limited graph connectivity (for a typical representation of a hardware graph, see Supplemental Figure S8). For nearly all problems of practical interest, the connectivity of the "logical problem" will differ from the Chimera architecture of D-Wave. This introduces the need to find a *minor embedding* of the hardware graph[42,43]. A minor embedding maps a logical problem qubit to a set of physical qubits such that for every coupling between pairs of logical qubits in the logical problem there exists at least one physical coupling between the corresponding sets of physical qubits. A minor embedding is found by performing a series of edge contractions, which effectively join vertices together, thereby allowing for a graph with fewer vertices but a higher degree of connectivity to be obtained[44]. For the results in this study, we used the "minorminer" package available on D-Wave's github [https://github.com/dwavesystems/minorminer].

In order to ensure that physical qubits are aligned and act as a single logical qubit (or "chain"), a strong coupling bias is introduced between physical qubits that comprise a logical qubit. Then, for a fixed embedding, the way the values of the couplings and local fields for a logical qubit are distributed among the physical qubits is known as "parameter setting". A built-in function provided by D-Wave[43] has been

used for parameter setting. By the embedding procedure and parameter setting, logical problems may be transformed into physical problems. Note that for one logical problem there may be many physical problems, depending both on the embedding and the parameter setting.

Ideally, once the strength of the coupling between logical qubits is determined, all solutions returned by D-Wave would correspond to valid logical solutions, i.e., all the physical qubits within a logical qubit would have the same spin (there would be no "broken chains"). However, due to the probabilistic nature of quantum annealing, as well as noise from different sources, there is often some percentage of solutions that have broken chains. To deal with broken chains D-Wave offers three options for "decoding" the solutions. The first is to discard all solutions with broken chains and collect an additional set of solutions ("discard"). Another option is to do a majority vote on the physical qubits that comprise a logical qubit, breaking ties with a random assignment ("majority vote decoding"). The last option is to go through the broken chains one by one and select the value for the spin that greedily minimizes the energy of the Hamiltonian of the logical problem ("energy minimization decoding"); i.e., it selects the spin that result in the greatest decrease in the energy of the Hamiltonian based on the current spin configuration. The likelihood of a solution having broken chains can be roughly adjusted by controlling a parameter J_c, the value of the strong coupling bias between physical qubits within a logical qubit; the larger the magnitude of J_c, the more likely will it be for the physical qubits within a logical qubit to have the same spin. The disadvantage of increasing the coupling bias too much is that it can wash out the details of the problem instances; thus, there is a tradeoff between getting solutions with many broken chains (which occurs when $|J_c|$ is too small) and getting solutions which may have lost the details of the original problem we are trying to solve (which occurs when $|J_c|$ is too large).

Based on these considerations, our strategy for collecting solutions was the following. First, we generated 20 embeddings based on the procedure mentioned above. The embedding with the smallest average number of physical qubits per logical qubit was used to obtain weights for all the training instances. For 2000Q, this embedding had 26.8 physical qubits per logical qubit on average, and 1747 qubits were used in total. The final embedding used for DW2X runs had 19.7 physical qubits per logical qubits on average, with a total of 887 qubits used. Then, for each training instance we queried the D-Wave chip for 1000 times with 10 spin-reversal transformations (or, gauges[45]) to mitigate parameter misspecifications from the machine. We then treated J_c as a hyper-parameter with values in the set $\{-0.5, -1, -3, -8, -16, -32\}$. All the parameters sent to the machine (both the ferromagnetic coupling J_c and the physical problem parameters h_i and J_{ij}) are normalized to fall between −1 and 1, per specifications of the machine. Finally, classical post-processing optionally may be performed on the broken chains. Energy-minimization and majority-vote decoding are quick and speed up collection of solutions; however, in principle, one could treat the post-processing approach as a hyper-parameter, but to avoid introducing too much classical post-processing and avoid spending too much time generating more solutions, we selected majority voting to post-process the solutions. All D-Wave anneals were run with an annealing time of 5μs. Note that we did not optimize the annealing time; doing so would introduce another hyperparameter and could improve results for D-Wave.

Derivation of the Ising Hamiltonian

Recall that we have written the probabilities for the first $K-1$ classes as:

$$Pr(y_i = k) = \frac{\exp \mathbf{w}_k^\intercal \mathbf{x}_i}{1 + \sum_{k=1}^{K-1} \exp \mathbf{w}_k^\intercal \mathbf{x}_i}, \tag{9}$$

with the probability of the Kth class as:

$$Pr(y_i = K) = \frac{1}{1 + \sum_{k=1}^{K-1} \exp \mathbf{w}_k^\intercal \mathbf{x}_i}. \tag{10}$$

By defining the probabilities of our classes in this way, we can reduce the number of sets of weights we have to train from K to $K-1$. The goal of training is to maximize the probability given the classes in the dataset, or equivalently to minimize the negative log-likelihood. we can express the negative log-likelihood as follows:

$$\mathcal{L} = -\log \prod_i Pr(y_i) \tag{11}$$

$$= -\sum_i \log Pr(y_i) \tag{12}$$

For simplicity, we define $z_i^{(k)} = w_k^{\intercal} x_i$, i.e., the inner product between the weights corresponding to the kth and the ith feature-vector. To continue, we consider splitting the above sum into terms over the first $K - 1$ classes and the Kth class:

$$\begin{aligned}
\mathcal{L} &= -\sum_i \log Pr(y_i) \\
&= -\sum_{i:y_i \in 1\ldots K-1} \log Pr(y_i) - \sum_{i:y_i=K} \log Pr(y_i = K) && (13) \\
&= -\sum_{i:y_i \in 1\ldots K-1} \left[z_i^{(y_i)} - \log\left(1 + \sum_{k=1}^{K-1} \exp z_i^{(k)}\right)\right] + \sum_{i:y_i=K} \log\left(1 + \sum_{k=1}^{K-1} \exp z_i^{(k)}\right) && (14) \\
&= -\sum_{i:y_i \in 1\ldots K-1} z_i^{(y_i)} + \sum_i \log\left(1 + \sum_{k=1}^{K-1} \exp z_i^{(k)}\right), && (15)
\end{aligned}$$

We can now take the second-order Taylor approximation around 0 for the second summation, expanding in $z_i^{(k)}$ gives us the following:

$$\begin{aligned}
\mathcal{L} &\approx -\sum_{i:y_i \in 1\ldots K-1} z_i^{(y_i)} + \sum_i \left[\log K + \frac{1}{K}\sum_k z_i^{(k)} + \frac{1}{2}\frac{K-1}{K^2}\sum_k \left(z_i^{(k)}\right)^2 - \frac{1}{2K^2}\sum_{k,j\neq k} z_i^{(j)} z_i^{(k)}\right] && (16) \\
&= -\sum_{i:y_i \in 1\ldots K-1} \mathbf{w}_{y_i}^{\intercal}\mathbf{x}_i + \sum_{i,k}\left[\frac{1}{K}\mathbf{w}_k^{\intercal}\mathbf{x}_i + \frac{K-1}{2K^2}\mathbf{w}_k^{\intercal}\mathbf{x}_i\mathbf{x}_i^{\intercal}\mathbf{w}_k\right] - \frac{1}{2K^2}\sum_{i,k,j\neq k}\mathbf{w}_j^{\intercal}\mathbf{x}_i\mathbf{x}_i^{\intercal}\mathbf{w}_k && (17) \\
&= \sum_{k=1}^{K-1}\mathbf{w}_k^{\intercal}\left(\sum_{i:y_i=k} -\mathbf{x}_i + \frac{1}{K}\sum_i \mathbf{x}_i\right) + \sum_{k=1}^{K-1}\mathbf{w}_k^{\intercal}\left(\frac{K-1}{2K^2}\sum_i \mathbf{x}_i\mathbf{x}_i^{\intercal}\right)\mathbf{w}_k - \sum_{k,j\neq k}\mathbf{w}_j^{\intercal}\left(\frac{1}{2K^2}\sum_i \mathbf{x}_i\mathbf{x}_i^{\intercal}\right)\mathbf{w}_k && (18) \\
&= \sum_{k=1}^{K-1}\mathbf{w}_k^{\intercal}(\mathbf{b}_k + \mathbf{h}') + \sum_{k=1}^{K-1}\mathbf{w}_k^{\intercal}\mathbf{J}'\mathbf{w}_k - \sum_{k=1}^{K-1}\sum_{j\neq k}\mathbf{w}_j^{\intercal}\mathbf{J}''\mathbf{w}_k && (19)
\end{aligned}$$

where

$$\mathbf{b}_k = \sum_{i:y_i=k} -\mathbf{x}_i, \tag{20}$$

$$\mathbf{h} = \frac{1}{K}\sum_i \mathbf{x}_i, \tag{21}$$

$$\mathbf{J}' = \frac{K-1}{2K^2}\sum_i \mathbf{x}_i\mathbf{x}_i^{\intercal}, \tag{22}$$

$$\mathbf{J}'' = \frac{1}{2K^2}\sum_i \mathbf{x}_i\mathbf{x}_i^{\intercal}. \tag{23}$$

In examining the derivation, one may ask whether it is reasonable to take an expansion to second-order near $z_i^{(k)} = 0$. We offer two brief arguments in support of this. The first is that a second-order approximation has been used to great success in other algorithms, such as XGBoost, a gradient-boosted algorithm that has seen much success recently in a variety of machine learning tasks. To speed up calculations, XGBoost uses a second-order approximation to calculate the objective function in a general setting. It is important to note, however, that for XGBoost (and other gradient-based methods) the weights are updated iteratively, whereas here we are presumably using a quantum annealer to directly evaluate the loss function. A second argument is that we are looking for a set of self-consistent solutions. We take the second-order approximation around 0, and if the optimization works properly, we will get results for which the approximation is valid.

Perhaps a more serious concern is that this expansion is not formally within the radius of convergence of the natural logarithm. Given this concern, care should be given to make sure that the difference in the approximation does not differ too greatly. One simple way to check this is to see whether there is a clear correlation between the energies (the approximation) and the original function we are trying to optimize (the log-likelihood). As long as there is good correlation, the approximation is reasonable. The correlation between the negative log-likelihood and the energy for the five binomial datasets is shown in Supplemental Figure S6 (more on this in the subsection titled "Performance metrics versus energy", below). For binary classification, the negative log-likelihood is equivalent to the logistic loss, $l = ln(1 + exp(-y_i w \cdot x_i))$, if we use the label convention $y_i \in \{-1,1\}$, or the binary cross-entropy loss, $l_{CE} = -y_i \, ln \, \sigma \, (w \cdot x_i) - (1 - y_i) \, ln\big(1 - \sigma(w \cdot x_i)\big)$ where $\sigma(z) = 1/(1 + exp(-z))$, if we use the convention $y_i \in \{0,1\}$. We sometimes refer to the negative log-likelihood as the logistic loss.

Post-processing Spin Configurations

In this section we describe our classical post-processing procedure to make use of all the spin configurations returned by D-Wave, SA and Random. We used all three methods to generate S different spin configurations (which we refer to as "weights") and sorted them by their Ising energy. Weights were then averaged together and the averaged weights that gave the best performance for some training metric was selected. More formally, let $\{w_i\}_{i=1}^{S}$be the set of S weights returned by the various methods. We define $w_{tmp}^k = \frac{1}{k}\sum_{i=1}^{k} w_i$ as the kth trial weight, and $\bar{f}_k = \frac{1}{|D^{train}|}\sum_{j \in D^{train}} f\big(y_j, \hat{y}_j^k\big)$ where f is the performance metric, D^{train} is the training data set, and $\hat{y}_j^k$ is the predicted output of the kth trial weights w_{tmp}^k on the jth training sample. The metrics of training performance include AUC, the logistic loss, and the accuracy. For the AUC and logistic loss we can directly use the predicted output (for binary classification, the predicted probability of the jth sample to be of class 1 is $\hat{y}_k = \sigma\big(w \cdot x_j\big)$. For the accuracy, we assign labels based on whether the predicted output is greater than 0.5. Applying this averaging procedure for a small set of weights allows us to increase the performance without sacrificing some of the robustness associated with discrete weights. Unless otherwise specified, for all Figures in the Main text and here, we used $S = 20$ and set f = AUC as the performance metric.

Supplemental References

1 Cerami, E. *et al.* (2012). The cBio Cancer Genomics Portal: An Open Platform for Exploring Multidimensional Cancer Genomics Data. *Cancer Discovery* **2**, 401- 404.

2 Koboldt, D. C. *et al.* (2012). VarScan 2: somatic mutation and copy number alteration discovery in cancer by exome sequencing. *Genome Research* **22**, 568-576.

3 Cibulskis, K. *et al.* (2013). Sensitive detection of somatic point mutations in impure and heterogeneous cancer samples. *Nature Biotechnology* **31**, 213-219.

4 McLaren, W., Gil, L., Hunt, S. E., Riat, H. S., Ritchie, G. R., Thormann, A., Flicek, P. & Cunningham, F. (2016). The ensembl variant effect predictor. *Genome Biology* **17**, 122.

5 Guðbjartsson, H. *et al.* (2016). GORpipe: a query tool for working with sequence data based on a Genomic Ordered Relational (GOR) architecture. *Bioinformatics* **32**, 3081-3088.

6 DePristo, M. A. *et al.* (2011). A framework for variation discovery and genotyping using next-generation DNA sequencing data. *Nature Genetics* **43**, 491-498.

7 McKenna, A. *et al.* (2010). The Genome Analysis Toolkit: a MapReduce framework for analyzing next-generation DNA sequencing data. *Genome Research* **20**, 1297-1303.

8 Van der Auwera, G. A. *et al.* (2013). From FastQ data to high-confidence variant calls: the genome analysis toolkit best practices pipeline. *Current Protocols in Bioinformatics* **43**, 11.10. 11-11.10. 33.

9 Anders, S., Pyl, P. T. & Huber, W. (2015). HTSeq—a Python framework to work with high-throughput sequencing data. *Bioinformatics* **31**, 166-169.

10 Robinson, M. D. & Oshlack, A. (2010). A scaling normalization method for differential expression analysis of RNA-seq data. *Genome Biology* **11**, R25.

11 Robinson, M. D., McCarthy, D. J. & Smyth, G. K. (2010). edgeR: a Bioconductor package for differential expression analysis of digital gene expression data. *Bioinformatics* **26**, 139-140.

12 Johnson, W. E., Li, C. & Rabinovic, A. (2007). Adjusting batch effects in microarray expression data using empirical Bayes methods. *Biostatistics* **8**, 118-127.

13 Leek, J., Johnson, W., Parker, H., Jaffe, A. & Storey, J. (2013). SVA: Surrogate Variable Analysis. R Package Version 3.

14 Gao, J. *et al.* (2013). Integrative analysis of complex cancer genomics and clinical profiles using the cBioPortal. *Sci. Signal.* **6**, pl1.

15 Beroukhim, R. *et al.* (2007). Assessing the significance of chromosomal aberrations in cancer: methodology and application to glioma. *Proceedings of the National Academy of Sciences* **104**, 20007-20012.

16 Hubbard, T. *et al.* (2002). The Ensembl genome database project. *Nucleic Acids Research* **30**, 38-41.

17 Zheng, Y., Joyce, B. T., Liu, L., Zhang, Z., Kibbe, W. A., Zhang, W. & Hou, L. (2017). Prediction of genome-wide DNA methylation in repetitive elements. *Nucleic Acids Research* **45**, 8697-8711.

18 Li, R. Y., Di Felice, R., Rohs, R. & Lidar, D. A. (2018). Quantum annealing versus classical machine learning applied to a simplified computational biology problem. *npj Quantum Information* **4**, 14.

19 Mott, A., Job, J., Vlimant, J.-R., Lidar, D. & Spiropulu, M. (2017). Solving a Higgs optimization problem with quantum annealing for machine learning. *Nature* **550**, 375-379.

20 Willsch, D., Willsch, M., De Raedt, H. & Michielsen, K. (2019). Support vector machines on the D-Wave quantum annealer. *arXiv preprint arXiv:1906.06283*.

21 Tibshirani, R. (1996). Regression shrinkage and selection via the lasso. *Journal of the Royal Statistical Society: Series B (Methodological)* **58**, 267-288.

22 Hoerl, A. E. & Kennard, R. W. (1970). Ridge regression: Biased estimation for nonorthogonal problems. *Technometrics* **12**, 55-67.

23 Hoerl, A. E., Kannard, R. W. & Baldwin, K. F. (1975). Ridge regression: some simulations. *Communications in Statistics-Theory and Methods* **4**, 105-123.

24 Kuhn, M. (2008). Building predictive models in R using the caret package. *Journal of Statistical Software* **28**, 1-26.

25 Boser, B. E., Guyon, I. M. & Vapnik, V. N. (ACM, 1992).in *Proceedings of the Fifth Annual Workshop on Computational Learning Theory.* 144-152.

26 Cortes & Vapnik, V. (1995). Support-vector networks. *Machine Learning* **20**, 273-297.

27 Breiman, L. (2001). Random forests. *Machine Learning* **45**, 5-32.

28 Breiman, L., Friedman, J., Stone, C. J. & Olshen, R. A. (Chapman & Hall, 1993). *Classification and regression trees*.

29 Hastie, T., Tibshirani, R. & Friedman, J. H. (Springer, 2016). *The Elements of Statistical Learning: Data Mining, Inference, and Prediction*. 2 edn.

30 Ng, A. Generative Learning algorithms. (2008).

31 Young, M. D., Wakefield, M. J., Smyth, G. K. & Oshlack, A. (2010). Gene ontology analysis for RNA-seq: accounting for selection bias. *Genome Biology* **11**, R14.

32 Winter, D. J. rentrez: An R package for the NCBI eUtils API. Report No. 2167-9843, (PeerJ Preprints, 2017).

33 Ward Jr, J. H. (1963). Hierarchical grouping to optimize an objective function. *Journal of the American Statistical Association* **58**, 236-244.

34 Albash, T. & Lidar, D. A. (2018). Adiabatic quantum computation. *Reviews of Modern Physics* **90**, 015002.

35 Kato, T. (1950). On the adiabatic theorem of quantum mechanics. *Journal of the Physical Society of Japan* **5**, 435-439.

36 Farhi, E., Goldstone, J., Gutmann, S. & Sipser, M. (2000). Quantum computation by adiabatic evolution. *arXiv preprint arXiv:quant-ph/0001106*.
37 Jansen, S., Ruskai, M.-B. & Seiler, R. (2007). Bounds for the adiabatic approximation with applications to quantum computation. *Journal of Mathematical Physics* **48**, 102111.
38 Rønnow, T. F., Wang, Z., Job, J., Boixo, S., Isakov, S. V., Wecker, D., Martinis, J. M., Lidar, D. A. & Troyer, M. (2014). Defining and detecting quantum speedup. *Science* **345**, 420-424.
39 Childs, A. M., Farhi, E. & Preskill, J. (2001). Robustness of adiabatic quantum computation. *Physical Review A* **65**, 012322.
40 Amin, M. H., Averin, D. V. & Nesteroff, J. A. (2009). Decoherence in adiabatic quantum computation. *Physical Review A* **79**, 022107.
41 Albash, T. & Lidar, D. A. (2015). Decoherence in adiabatic quantum computation. *Physical Review A* **91**, 062320.
42 Choi, V. (2008). Minor-embedding in adiabatic quantum computation: I. The parameter setting problem. *Quantum Information Processing* **7**, 193-209.
43 Cai, J., Macready, W. G. & Roy, A. (2014). A practical heuristic for finding graph minors. *arXiv preprint arXiv:1406.2741*.
44 Robertson, N. & Seymour, P. D. (1984). Graph minors. III. Planar tree-width. *Journal of Combinatorial Theory, Series B* **36**, 49-64.
45 Boixo, S., Albash, T., Spedalieri, F. M., Chancellor, N. & Lidar, D. A. (2013). Experimental signature of programmable quantum annealing. *Nature Communications* **4**, 2067.
